# First-in-human high dose AAV9 intrathecal gene therapy for paediatric CLN7 disease: a phase 1, open-label, single ascending dose, non-randomised clinical trial

**DOI:** 10.1016/j.ebiom.2025.106044

**Published:** 2025-11-27

**Authors:** Benjamin M. Greenberg, Berge Minassian, Souad Messahel, Veronica Bordes Edgar, Andrea Lowden, Hamza Dahshi, Emily R. Nettesheim, Hoang H. Nguyen, Samuel Hughes, Alagar R. Muthukumar, Kalayarasan Srinivasan, Susan Iannaccone, Ganapathy Varadarajan, Steven J. Gray, Saima N. Kayani

**Affiliations:** a Department of Neurology, University of Texas Southwestern Medical Center, Dallas, TX, USA; b Department of Paediatrics, University of Texas Southwestern Medical Center, Dallas, TX, USA; c Elpida Therapeutics, Encina, CA, USA; d Peter O'Donnell Brain Institute, University of Texas Southwestern, Dallas, TX, USA; e Children's Health Dallas, Dallas, TX, USA; f Department of Ophthalmology, University of Texas Southwestern Medical Center, Dallas, TX, USA; g Department of Pathology, University of Texas Southwestern Medical Center, Dallas, TX, USA; h Department of Molecular Biology, University of Texas Southwestern Medical Center, Dallas, TX, USA; i McDermott Center for Human Growth and Development, University of Texas Southwestern Medical Center, Dallas, TX, USA; j St. Jude Children's Research Hospital, Center for Experimental Neurotherapeutics, Memphis, TN, USA; k Case Western Reserve University, Cleveland, OH, USA

**Keywords:** Neuronal ceroid lipofuscinoses type 7 (CLN7), Gene replacement therapy, AAV9, Intrathecal gene therapy

## Abstract

**Background:**

Neuronal Ceroid Lipofuscinoses type 7 (CLN7) is a paediatric lysosomal storage disease caused by mutations of the MFSD8 gene. Affected children have normal early development, but then suffer from progressive cognitive, motor, verbal, and visual decline. Ataxia and myoclonic epilepsy are predominant features of the condition, and there are no effective therapies. Death usually occurs by approximately age 11 years. While adeno-associated virus serotype 9 (AAV9) based gene therapy holds promise for treating monogenetic neurologic disorders, the impact of this intervention is limited by the maximum safe tolerable dose and the host immune response to the capsid and gene product. This study sought to confirm the safety of high dose intrathecal AAV-based gene therapy under a comprehensive immunosuppression regimen.

**Methods:**

This was a two-year open label, dose escalation, phase 1 first-in-human study of AAV9-based intrathecal gene therapy for CLN7. 4 participants (1 low dose, 3 high dose) were followed at regular intervals with blood work, CSF analysis, EEG, MRI, and measures of neurologic and neuropsychological function.

**Findings:**

This study provided evidence of safety for high dose intrathecal AAV9 based gene therapy in CLN7 disease under a specific immunosuppression regimen. Additionally, this study provides preliminary evidence of efficacy for this gene therapy.

**Interpretation:**

High dose intrathecal AAV based gene therapy can be pursued with adequate immunosuppression and monitoring for immune responses to the gene product. Additional long-term monitoring of the immune system during tapering of immunosuppression is needed to identify potential reactions to the gene product.

**Funding:**

This study was funded by The Batten's Hope Foundation, Mila's Miracle Foundation, Children's Health Dallas and Philanthropic Gifts to UT Southwestern. In addition, Emily R. Nettesheim received funding from NIH training grant 5T32GM131945-03 and Hamza Dahshi was supported in part by NIH award T32 GM152319.

**Research in context**

**Evidence before this study**

Neuronal Ceroid Lipofuscinoses type 7 (CLN7) is considered to be a uniformly fatal paediatric neurodegenerative disorder with progressive loss of cognitive, motor, verbal and vision capabilities. This lysosomal storage disease results from mutations of the MFSD8 gene and there are no available therapies. A systemic search was conducted with keywords: CLN7/MFSD8/gene replacement therapy/AAV9 gene therapy. The search included databases like PubMed/Medline, OVID and preprint servers like bioRxiv and medRxiv, and revealed that no peer-reviewed human safety and efficacy data through present or past therapeutic trials involving gene replacement therapy for CLN has been published to date. In addition, searches on clinicaltrials.gov and family advocacy groups e.g., rare disease news and batten disease news revealed no ongoing clinical trials for CLN7.

Adeno-associated virus serotype 9 (AAV9) based gene therapy holds promise as a way to correct monogenetic conditions, but prior preclinical studies and clinical trials have raised concerns about the maximum safe dose in the setting of potential immune responses to the viral capsid and the gene product, along with drug class-related effects, such as dorsal root ganglia (DRG) toxicity. Developing protocols that allow for higher doses of viral based gene therapy is needed to increase the success of these potentially curative interventions.

**Added value of this study**

This study examined a combination immunotherapy protocol to prevent immune responses to AAV9 based gene therapy for CLN7. Participants in this study tolerated the highest dose of intrathecal AAV gene therapy reported to date, showed some evidence of a clinical benefit, and confirmed the need for immunologic monitoring.

**Implications of all the available evidence**

AAV9 gene therapy for CLN7 disease can be delivered at high doses intrathecally, but careful monitoring is required. Additionally, future studies should consider earlier intervention in the course of this disorder to improve the potential magnitude of treatment benefits to participants.

## Introduction

CLN7 disease is an autosomal recessive condition caused by biallelic pathogenic variants in the MFSD8 gene which encodes for a lysosomal membrane protein.^1^^,^^2^ Mutations lead to intracellular accumulation of storage material i.e., lipofuscin, a complex mixture of proteins and lipids.^3^ Mutations in MFSD8 lead to impaired autophagy, due to accumulation of undegraded substrates, and disrupts normal lysosomal function with further accumulation of cellular waste products. This in conjunction with mitochondrial dysfunction and neuroinflammation ultimately contributes to neuronal dysfunction and degeneration, although the precise mechanism of neurodegeneration is not completely elucidated.^4^

A review of 35 papers (2004–2024) describing 131 patients with CLN7 revealed insights into disease onset and presentation, with our analysis focusing on 76 patients diagnosed before age six to align with our late-infantile-onset clinical trial. Initial years of normal development precede cognitive and physical regression in early childhood, with the average age of disease onset for the late infantile group being 3.8 years.^1^^,^^5^, ^6^, ^7^, ^8^, ^9^ The first symptoms involve general developmental delay and cognitive decline. All patients exhibit language decline, with patients ultimately progressing to non-verbal status. Brain MRI reveals cerebellar atrophy, cerebral atrophy and increased white matter signal intensity, while EEGs are abnormal. Typically, onset of myoclonic seizures is described around 4.5 years of age.^1^^,^^2^^,^^5^^,^^9^, ^10^, ^11^, ^12^, ^13^, ^14^ Increase in the frequency and severity of the seizures typically occurs about 2 years after initial onset of seizures (12 patients) and become uncontrollable with medications.^2^^,^^5^^,^^10^^,^^11^^,^^15^ Vision loss, similar to retinitis pigmentosa, is reported at an average age of 4.9 years, progressing to blindness. Motor function is also severely affected, with signs of ataxia appearing around at an average age of 3.6 years, leading to progressive loss of mobility and eventual full bedridden status by approximately 10.4 years. This loss of mobility typically comes towards the end of the lifespan, which our review found to be around 11.5 years of age (19 patients).^1^^,^^2^^,^^5^, ^6^, ^7^, ^8^, ^9^, ^10^, ^11^, ^12^, ^13^^,^^15^, ^16^, ^17^, ^18^, ^19^, ^20^ There are no known effective therapies for CLN7.

AAV9 gene therapy can enhance CNS delivery through direct CSF administration via intraventricular, cisternal, or intrathecal routes, but are limited to single doses due to immune responses.^21^ Preclinical studies in rodents demonstrated effective central nervous system (CNS) biodistribution, safety and efficacy of an intrathecally-delivered AAV9/CLN7 vector^22^, and other high dose AAV9-based studies have demonstrated similar CNS biodistribution in large animals.^23^^,^^24^ Establishing a safe, high dose of AAV9 intrathecal gene therapy and optimising immunosuppression regimens are crucial for maximising clinical benefits and minimising immune-related adverse effects.

This phase 1, first in human study of delivering a healthy copy of the MSFD8 gene via high doses of the AAV9 vectors in paediatric patients with CLN7 was completed at a single centre. The primary outcome of this study was to determine the safety of this high dose of intrathecal therapy in the setting of a combination immunosuppressive regimen. The safety and efficacy results of this study are presented here.

## Methods

### Ethics

This study was conducted in accordance with the Declaration of Helsinki and Good Clinical Practice guidelines. Ethical approval was granted by the central Institutional Review Board (IRB), ADVARRA, under protocol number Pr00050219. The trial was conducted at the University of Texas Southwestern Medical Center and Children's Health Dallas, under an investigator-initiated Investigational New Drug (IND) application. The study is registered at ClinicalTrials.gov (NCT04737460) and was officially opened for enrolment on May 4th, 2021.

Prior to participation, written informed consent was obtained from all participants or their legally authorised representatives, as appropriate. All procedures involving human participants were carried out in full compliance with applicable national and institutional ethical standards.

### Study design

The trial was designed as a phase I, open label, non-randomised single ascending intrathecal dose trial which would enrol up to 4 participants.

### AAV9/CLN7 vector

The self-complementary AAV9 vector design is as described, using the minimal JeT promoter to drive expression of the MFSD8 gene.^22^ The vector was produced by Vigene Biosciences and formulated in phosphate buffered saline with 5% D-sorbitol and 0.001% pluronic F68. The Certificate of Analysis is provided as supplemental information.

The study design was developed based on the GAN protocol, which served as a framework for evaluating this similar gene therapy approach, and consistent in methodology and safety monitoring.^25^ Suitable candidates were identified through clinical referrals from their treating neurologists and the investigators pre-screened candidates on a first come first serve basis. Each participant had confirmed diagnosis of CLN7 disease by genetic testing with either homozygous or bi-allelic pathogenic variants in MFSD8 gene. Participants were required to have active symptoms consistent with CLN7 disease but have not progressed to end stage disease status. The first participant received a total intrathecal dose of 5 x 10^14^ vg of AAV9/CLN7 therapy. Following DSMB review of 60-day post-infusion safety data, clearance was granted for subsequent participants to receive the high-dose intrathecal therapy of 1 x 10^15^ vg per participant. The dose was picked based upon the pre-clinical studies that provided strong translational support for high-dose IT administration in human trials to achieve widespread CNS and PNS distribution, robust transgene expression and meaningful clinical benefit. High-dose IT delivery was well tolerated in both rodent and large animal models, with no significant toxicity observed.^22^, ^23^, ^24^ Additional DSMB meetings for safety review were held at 60-day post dose intervals for the rest of cohort. The patients were dosed at 90-day interval after getting clearance from DSMB.

In order to reduce the risk of host immune response to AAV9 based therapy, participants were started on a prophylactic immune suppression regimen. For immune suppression, participants were stratified into two groups based on cross-reactive immunological material (CRIM) status:

The first group with mutations with any predicted residual MFSD8 protein presence, would be considered CRIM positive and placed on immune modulation with enteral prednisone and sirolimus.

The second group with predicted null mutations (no residual endogenous MFSD8 protein), would be considered CRIM negative and placed on enteral prednisone, sirolimus, and tacrolimus for immune modulation.

### Safety outcomes

The primary outcome of this study was the safety and tolerability of single high dose AAV9/CLN7 gene therapy administered intrathecally quantified by the incidence and severity of treatment related serious adverse events. MRI brain data, blood work, CSF data and nerve conduction studies were collected to monitor for evidence of an immune response, transaminitis, bone marrow reactivity or DRG toxicity. Safety assessments were preformed using standard-of-care clinical and laboratory evaluations, including but not limited to complete blood counts, serum chemistries, urinalysis and cerebrospinal fluid analysis. MRI brain studies, obtained as part of safety monitoring, were reviewed and interpreted by board-certified neuroradiologists and performed uniformly per clinical guidelines and standard operating procedures.

Safety was monitored in two periods. An initial period from dosing to 24 months, then annual monitoring for an additional 5 years for long-term safety.

### Efficacy outcomes

The secondary outcome was to evaluate the efficacy of the therapy as quantified by a series of standardised tests for motor function and seizure burden. Specifically, these were the Clinical Global Impression Scale (CGI), the 2-min timed walk test, the paediatric balance scale, and the Gross Motor Function Measure (GMFM). In addition, Electroencephalography (EEG) and MRI Brain were completed which could potentially serve as surrogate measures.

Participants underwent neuropsychological assessment conducted by a board-certified neuropsychologist in their native language or with a professional interpreter. The following measures were administered in this order: Mullen Scales of Early Learning (Mullen)^26^, Vineland Adaptive Behavior Scales, 3rd Edition Comprehensive Interview (Vineland-3)^27^, Infant Toddler Quality of Life Questionnaire (ITQOL)^28^ for children under 5 years and the parent reported Quality of Life Inventory-Disability (QI-Disability) questionnaire^29^ for children 5 years of age and older.

### Sample size

The number of participants was determined based upon the availability of the study drug. The first participant received a low dose of 5 x 10^14^ vg of AAV9/CLN7 via intra thecal route. DSMB reviewed the 60-day post-infusion safety data and clearance was granted for the next participant to receive the high-dose intrathecal therapy of 1 x 10^15^ vg AAV9/CLN7. Additional DSMB meetings for safety review were held at 60-day post dose intervals for the rest of cohort. The patients were dosed at 90-day interval after getting clearance from DSMB.

### Randomisation

Patients were assigned to receive either a low-dose or high dose based on first come, first served enrolment scheme. The first patient enrolled in the study would receive low dose and the subsequently enrolled patients would receive the high dose.

### Statistics

Given the small sample size and primary outcome of safety, no formal statistics were performed on this cohort. All data is presented in a descriptive format. Data analysis and visualisation were conducted using R (version 4.4.0).

### Data handling

All data were recorded manually during assessments and stored in individual binders for each patient. No digital database was used for initial data entry. The recorded scores were subsequently transcribed into a password-protected digital database with restricted access to authorised study personnel only. No automated data processing tools were used.

Data completeness varied across efficacy outcomes and visits, depending on patient availability and ability to complete the assessments. If a data point was missing, it was plotted as such without imputation or statistical adjustments. Missing data primarily arose due to two reasons: the inability to perform certain assessments at specific visits and missed visits where the patient did not attend the scheduled assessment. The extent of missing data was not systematically tracked for missingness patterns. Missing data could introduce bias in interpreting individual trends, but as the study did not rely on complete case analysis, this was not a limiting factor. Data analysis and visualisation were conducted using R (version 4.4.0).

### Role of Funders

The Batten's Hope Foundation and Mila's Miracle Foundation supported the production of the AAV9/CLN7 vector, but did not have input into study data interpretation of this publication. Additional funding for the project came from Children's Health Dallas and an anonymous philanthropic gift to Dr. Gray's laboratory at UT Southwestern. Ms. Nettesheim also received stipend funding for one year through NIH training grant 5T32GM131945-03. None of them had any input into study design, data collection, data analyses, interpretation, or writing of report.

## Results

### Study participants

Twenty participants were pre-screened on a first come, first serve basis. Four participants (3 female and 1 male participant) were enrolled in this study, over an enrolment period spanning 10 months. The participants, aged between 4 and 5 years old, represented a diverse group, both geographically and linguistically.

Participants’ clinical and demographic data are summarised in Table 1.

**Table 1:** Demographic data.

Category	Patient 1	Patient 2	Patient 3	Patient 4
Sex^a^	F	M	F	F
Ethnicity	Hispanic	Romanian	Arab	Caucasian
Age at Onset (Years. Months)	2	3	3	3
Age at Dosing (Years. Months)	5.1	4.7	5.11	4.1
Symptoms at onset	Language regression, seizures	Myoclonus, Seizures	Falls, myoclonus	Language and fine motor regression
Pathogenic Variants	MFSD8 c.103C > T (p.Arg35Ter) Homozygous Stop gained	MFSD8 c.1373C > A (p.Thr458Lys) Homozygous Missense	MFSD8 c.198+2T > C Homozygous GT Splice donor	MFSD8 C.1444C > T (p.Arg482Ter) C.1206del(p.Ile403LeufsTer11) Compound heterozygous Stop gain/Frameshift
Dose	5x10^14^ vg	1x10^15^ vg	1x10^15^ vg	1x10^15^ vg
CRIM status	Negative	Positive	Negative	Negative
Time post-dosing (months)	40	36	32	29
Weight at the time of dosing (kg)	24	20.4	19.7	15.5
Height at the time of dosing (cm)	116	112.5	119	100
Anti-Seizure medications at the time of dosing	Clobazam, Lamotrigine	Valproic acid Levetiracetam	Valproic acid	Levetiracetam

Table 1. Demographic data of the four patients enrolled in the trial. This table provides an overview of key demographic characteristics, including age at enrolment, sex, ethnicity, and baseline clinical features, to contextualise the study population and support the interpretation of trial outcomes.

a Reported by patient/caregiver.

**Fig. 1:**
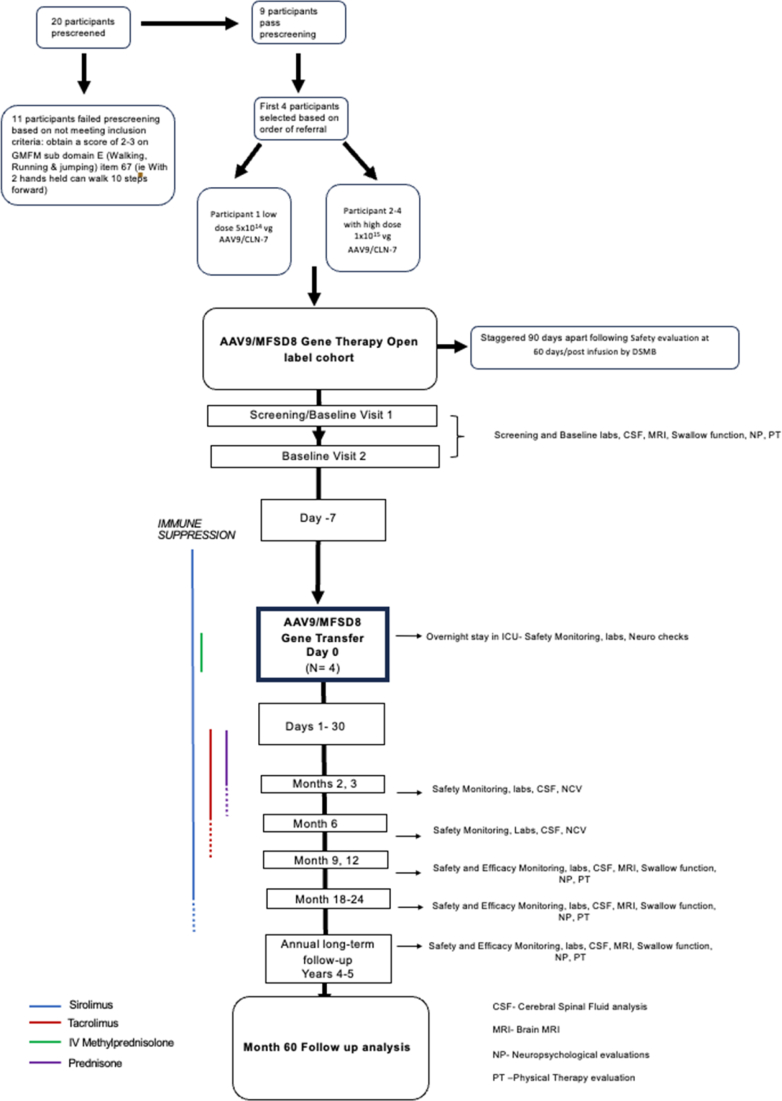
**Study schematic for open-label trial for AAV9/MFSD8**. A schematic representation of the immune modulation protocol for CRIM-positive (CRIM+) and CRIM-negative (CRIM−) participants, outlining its alignment with study visits. The diagram also illustrates the timing of neuroimaging procedures, cerebrospinal fluid (CSF) collection, and the ELISpot assay schedule. Serial brain MRIs and lumbar punctures were performed at day 90, day 180, day 360, day 540 (MRI only) and day 720 to assess for any evidence of an immune response to the vector or transgene. Participants predicted to be positive for cross-reactive immunological material (CRIM) were managed with prednisone and sirolimus. Participants who were predicted to be CRIM negative, based on gene sequencing results, were managed with prednisone, tacrolimus and sirolimus. Twenty participants were pre-screened, 11 participants failed prescreening based on not meeting inclusion criteria: obtain a score of 2–3 on GMFM sub domain E (Walking, Running & jumping) item 67 (i.e., With 2 hands held can walk 10 steps forward). 9 participants passed prescreening and 4 were selected for treatment. Enrolment proceeded in sequential order once eligibility was confirmed.

Participants were followed for 2 years with safety assessments occurring at regular intervals. The schedule of clinical and lab monitoring is summarised in Fig. 1. Serial brain MRIs and lumbar punctures were performed at baseline, day 90, day 180, day 360, day 540, and day 720 to assess for any evidence of an immune response to the vector or transgene. After 24 months, participants transitioned to a long-term follow up protocol for 5 years.

### Primary safety endpoint and adverse events

Overall, both low dose and high dose AAV9/CLN7 were well tolerated. Additionally, there were no serious adverse events from immune suppression regimen. There have been no clinically significant cytopenias. In addition, lab data revealed stability of liver and kidney function and no evidence of microangiopathy (Supplementary Figure 1). Immunosuppression drug trough levels indicate that participants had reached therapeutic range with no toxic effects noted.

There were 57 adverse events reported in the 4 participants treated over 24 months (Table 2 and Supplementary Table 1). Of those, 2 were considered treatment emergent adverse events (TEAE's) to be possibly or probably related to AAV9/MFSD8 treatment in 2 of the 4 participants and included vomiting (1 of 4 participants) and pleocytosis (1 of 4). There were no reported adverse events (AEs) potentially related to the administration procedure.

**Table 2:** Adverse Events represented as number of events per organ class.

System Organ Class	Event	% of total AEs	Possibly related to IP
Infections and infestations	10	17.2	0/10
Gastrointestinal disorders	9	15.5	0/9
Nervous system disorders	7	8.6	2/5
Renal and urinary disorders	2	3.4	0/2
Musculoskeletal and connective tissue disorders	6	10.3	0/6
Respiratory, thoracic and mediastinal disorders	1	1.7	0/1
Injury, poisoning, and procedural complications	5	8.6	0/5
General disorders and administration site conditions	18	34.4	0/20

Table 2: Summary of adverse events (AEs) categorised by system organ class (SOC). The table presents the number of AEs per SOC, the percentage of total AEs each category represents, and the number of events that were possibly related to the investigational product (IP). General disorders accounted for the largest proportion of AEs (34.4%), followed by infections and infestations (17.2%) and gastrointestinal disorders (15.5%). No AEs in the infections, gastrointestinal, renal and urinary, musculoskeletal, respiratory, injury-related, or general disorders categories were considered related to the IP. Two events are within the nervous system disorders category and were possibly related to IP.

Most of the AEs were graded 1 for severity (90% at the low dose and 72% at the high dose), with 9 being Grade 2 (5% at the low dose and 22% at the high dose.), and 2 (0% at the low dose and 6% at the high dose) being Grade 3. Grade 2 AEs/SAEs included nasal congestion, leg and foot pain, a broken clavicle, increased seizure frequency, COVID infection, urine infection and fever. Grade 3 AEs/SAEs included vomiting and pleocytosis. There were no Grade 4 or 5 adverse events for either dose levels.

The first TEAE thought to be related to the investigational product occurred following steroid tapering. The participant predicted to be CRIM positive, CLN7-02, received immune modulation with prednisolone and sirolimus per protocol. Participant's CSF profile revealed pleocytosis in their CSF on their Day 180 visit (after steroid taper was complete). Participant 2 (CLN7-02) was restarted on steroids and had a prolonged steroid taper. The participant underwent an additional safety MRI and lumbar puncture 4 weeks post Day 180 visit. The repeat analysis showed resolution of CSF pleocytosis and MRI of the brain was stable with no evidence of inflammation. The participant was maintained on sirolimus long term like the CRIM negative cohort. CSF sampling revealed a second event of mild pleocytosis for the same participant, on Day 720 visit. The participant was positive for para-influenza virus and clinically presented with nasal congestion, with no new or worsening neurologic symptoms (i.e., had stable mental status with no increased seizure frequency) and remained clinically stable.

The second TEAE categorised as possibly or probably related occurred within the first few days after receiving AAV9/MFSD8 for fourth participant (CLN7-04) and was resolved within 7 days of onset. The participant had vomiting 3 days after treatment, requiring hospitalisation for monitoring. The participant was managed with supportive care and intravenous hydration. Oral medications were held, and prednisolone was converted to intravenous steroids until the participant tolerated enteral feeding.

**Fig. 2:**
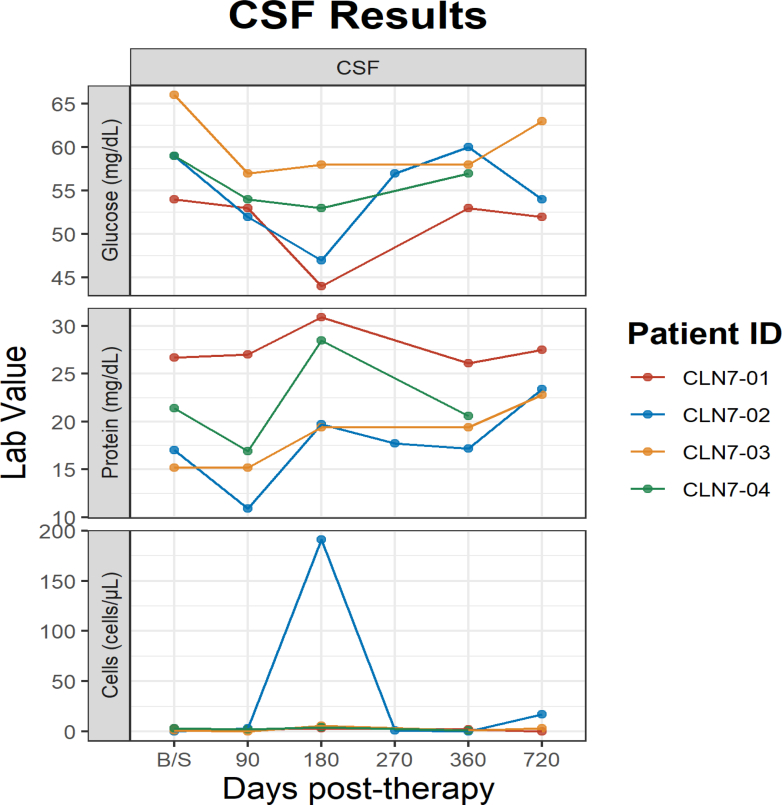
**CSF results**. CSF glucose, protein and cell count values of four participants at baseline and follow up visits at 90-,180-, 270-, 360- and 720- days post therapy. Patient 2 (CLN7-02) had CSF pleocytosis on Day 180 visit (after steroid taper was complete). Patient 2 was restarted on steroids and had a prolonged steroid taper due to CSF pleocytosis. CSF sampling revealed a second event of mild pleocytosis (nucleated cells-17) for the same patient, on Day 720 visit. Patient was positive for para-influenza virus and clinically presented with nasal congestion, with no new neurologic symptoms. CLN-01 received the smaller dose of 5 x 10^14^ vg. The rest received the higher dose of 1 x 10^15^ vg.

Three participants, CLN7-01, CLN7-03 and CLN7-04 received prednisolone, sirolimus, and tacrolimus due to their predicted CRIM negative status. All completed a steroid taper with no anti-capsid or anti-gene product positive ELISpot results and had normal CSF results which are reported in Fig. 2. ELISpot were performed at regular intervals and demonstrated no detectable T-cell responses to either the AAV9 vector or the MFSD8 transgene following treatment.

**Fig. 3:**
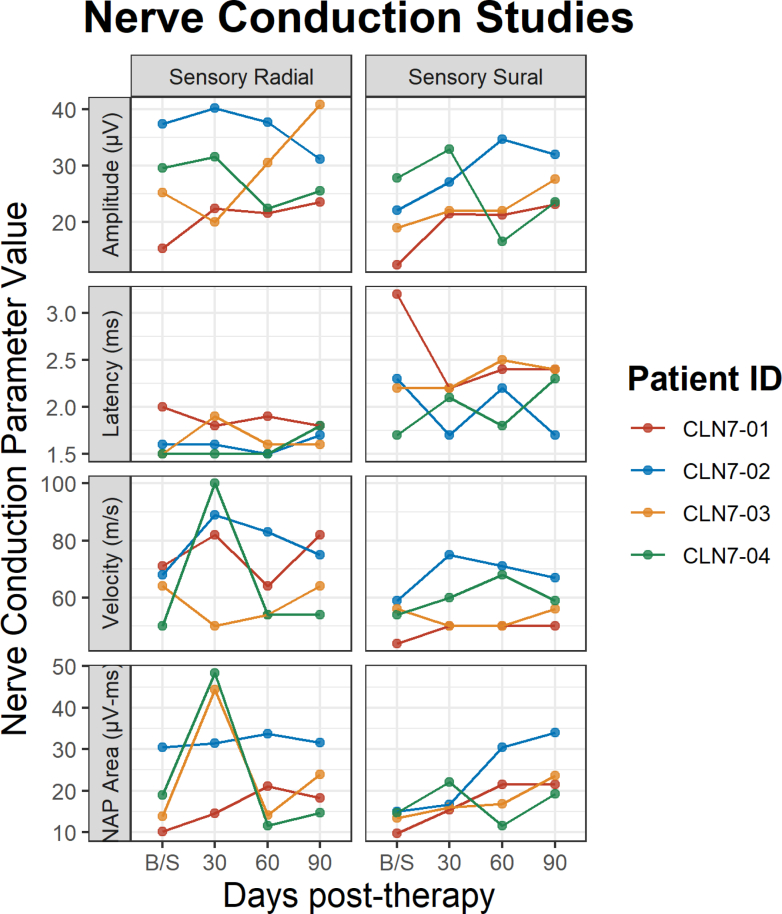
**Nerve conduction studies**. Nerve conduction study results of radial and sural nerves, showing amplitude, latency, velocity and NAP area for all 4 participants at baseline and follow up visits at 30-, 60- and 90-days post therapy. CLN-01 received the smaller dose of 5 x 10^14^ vg. The rest received the higher dose of 1 x 10^15^ vg.

Monitoring for DRG pathology involved serial exams and electrophysiology studies. Serial nerve conduction studies failed to identify any evidence of peripheral neuropathy or altered DRG function (Fig. 3). There was no evidence of new autonomic dysfunction over the course of the trial in any of the participants.

### Secondary endpoints

Initial neuroimaging for all participants revealed findings consistent with CLN7 disease. At baseline, all participants had mild to moderate generalised atrophy with abnormal signal intensity in periventricular and deep cerebral white matter. Progressive changes were seen in neuroimaging that were consistent with underlying CLN7 disease. Due to lack of matched data from a natural history cohort, we cannot comment on whether the rate of progression was altered in the treated cohort. In addition, no noticeable difference is appreciated between the CLN7-01 (recipient of low dose gene therapy) and CLN7-02, CLN7-03 and CLN7-04 (all who received high dose gene therapy).

Serial EEGs were acquired for all of the participants beginning with a baseline assessment. Over the course of the study all of the EEGs were abnormal showing variable degrees of diffuse slowing of the interictal background along with generalised and multifocal epileptiform discharges. In a single participant, CLN7-03, a focal motor seizure with partial awareness was captured. This event was characterised clinically by eyelid myoclonia, triggered by photic stimulation with an electrographic correlate in the bi-occipital regions. These findings did not change significantly over the course of the study. Of note, CLN7-03 received high dose gene therapy.

**Fig. 4:**
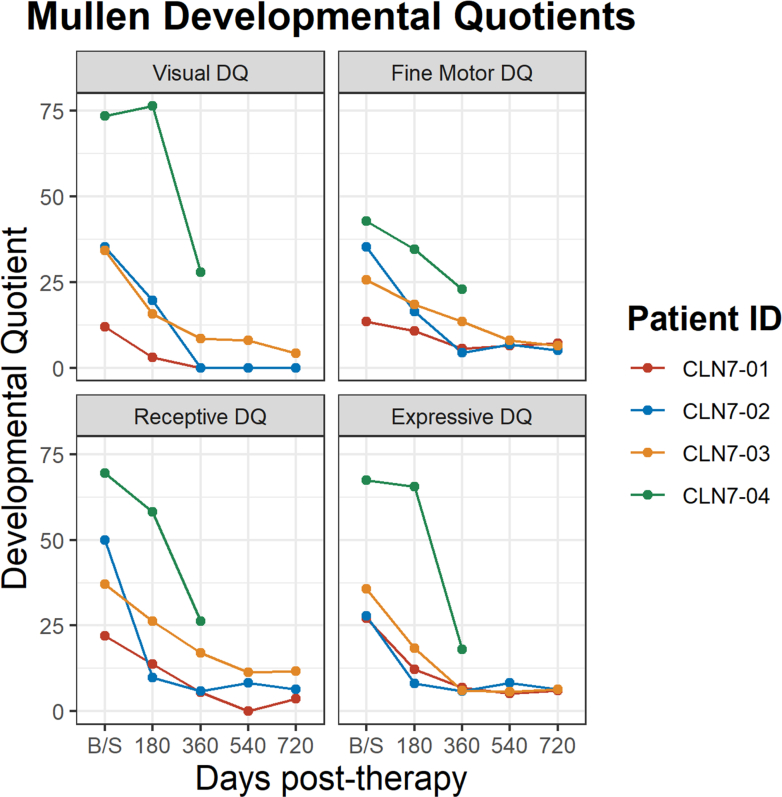
**Mullen development quotients**. Developmental Quotients Scores from Mullen for participants 1–4. The scores represented are the Developmental Quotient (DQ) for Visual Reasoning, Fine Motor, Receptive Language, and Expressive Language subscales. CLN-01 received the smaller dose of 5 x 10^14^ vg. The rest received the higher dose of 1 x 10^15^ vg. CLN-04 lacks data for data 540 and 720 as the family wished to avoid travel to the study site and invasive procedures.

Neuropsychologic function tests were acquired prospectively to seek evidence of efficacy. When possible, raw scores were converted to age equivalents and subsequently to developmental quotients (age equivalent/chronological age ∗ 100) as opposed to norm-referenced scores. The children's cognitive abilities as measured by the Mullen were all near the floor (i.e., lowest possible) of the instrument when examining norm-referenced scores by day 180 for CLN7-01, CLN7-02, and CLN7-03; and by day 360 for CLN7-04 (Supplementary Figure 2) Therefore, the composite does not provide sufficient information. As such, Developmental Quotients best captured their functioning on which significant decline was seen by day 360 for all participants with plateauing seen for the subsequent timepoints. CLN-03 had the most gradual decline especially in receptive language and fine motor skills. CLN-04 was the highest functioning initially but sharply declined between days 180 and 360 especially in visual reasoning and language (Fig. 4).

**Fig. 5:**
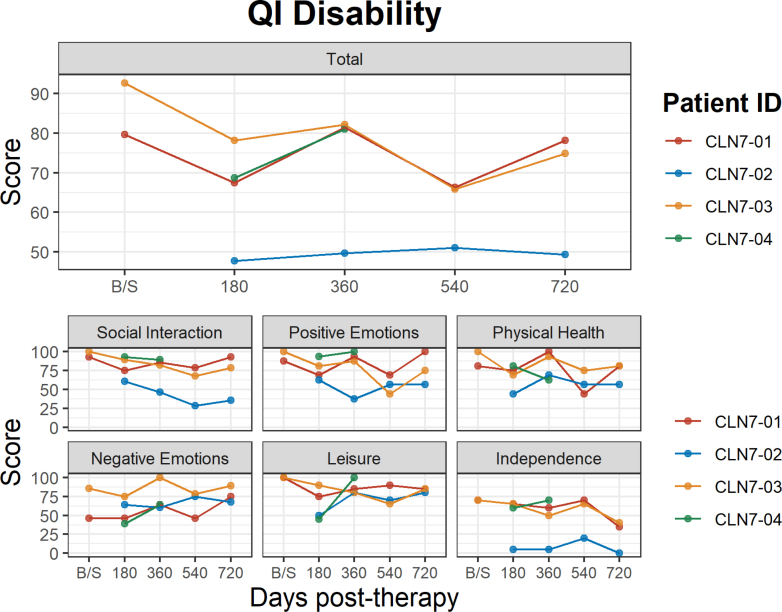
**Quality of life and disability.** The QI-Disability scores for participants 1–4. The scores represented include Total, Social Interactions, Positive Emotions, Physical Health, Negative Emotions, Leisure, and Independence subscales. CLN-01 received the smaller dose of 5 x 10^14^ vg. The rest received the higher dose of 1 x 10^15^ vg. CLN-04 lacks data for data 540 and 720 as the family wished to avoid travel to the study site and invasive procedures.

The QI-Disability was administered to assess the participants quality of life (QOL). CLN7-01, patient who received low dose gene therapy and CLN7-03, who received high dose gene therapy had a decline in QOL scores between baseline and day 180, as well as between days 360 and 540, but there was a reported increase in QOL from days 180–360 and again between days 540 and 720, whereas CLN7-02 reported a low QOL throughout the study (Fig. 5). The QOL score for CLN7-04 increased from just below 70 to above 80 between day 180 and 360 (Fig. 5). After baseline, day 360 proved to be the highest point of QOL for all participants except CLN7-02 with scores just above 80 for all three. However, there was variable impact by each of the subdomains whereby social interactions, positive emotions, and leisure skills remained positive for CLN7-01, CLN7-03 and CLN7-04 (Fig. 5). Declines and variability were greatest on physical health and independence. CLN7-02 had the lowest ratings on all except for negative emotions (e.g., behavioural outbursts, withdrawn behaviour) when they were rated more like the other participants.

**Fig. 6:**
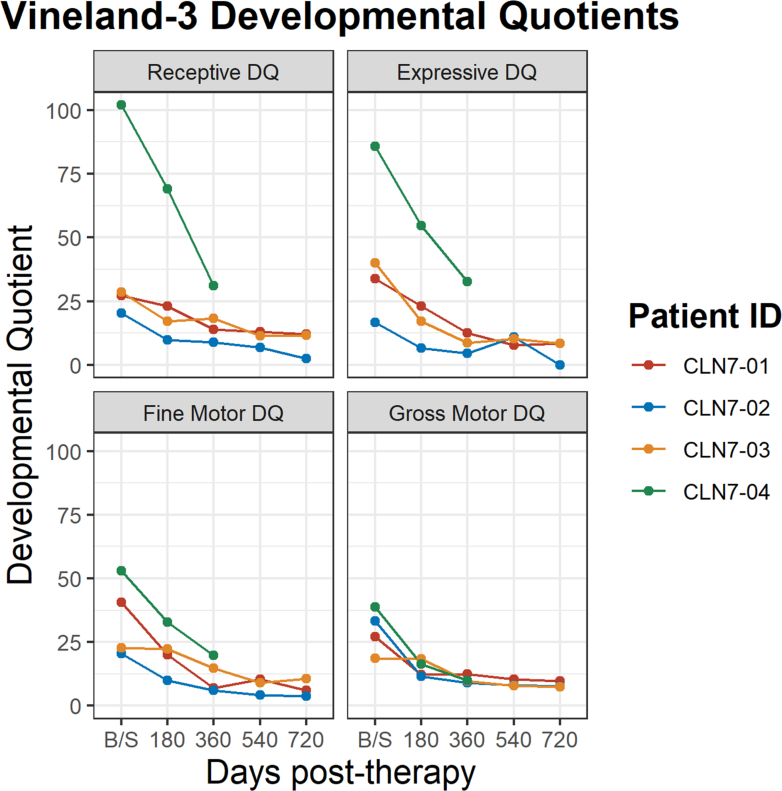
**Vineland −3 developmental quotients**. Developmental Quotients Scores from the Vineland-3 for participants 1–4. The scores represented are the Receptive Language, Expressive Language, Fine Motor, and Gross Motor subscales. CLN-01 received the smaller dose of 5 x 10^14^ vg. The rest received the higher dose of 1 x 10^15^ vg. CLN-04 lacks data for data 540 and 720 as the family wished to avoid travel to the study site and invasive procedures.

The Vineland-3 was administered to measure changes in adaptive behaviours (i.e., skills for functional independence). No participant had improvement in scores, but three participants had relative stabilisation of their composite score between day 180 and 720 (Supplementary Figure 3) The greatest declines were seen in the areas of communication and motor skills and resulted in scores at the floor of the measure. Skills in the areas of daily living and social skills were generally more stable. Similar to scores on the Mullen, CLN7-04 started out the highest but showed sharpest decline especially in the area of communication. Developmental Quotients were examined for Receptive and Expressive Language as well as Fine and Gross Motor skills (Fig. 6). This revealed more gradual declines in language as well as in fine motor skills with a general plateauing by day 360.

**Fig. 7:**
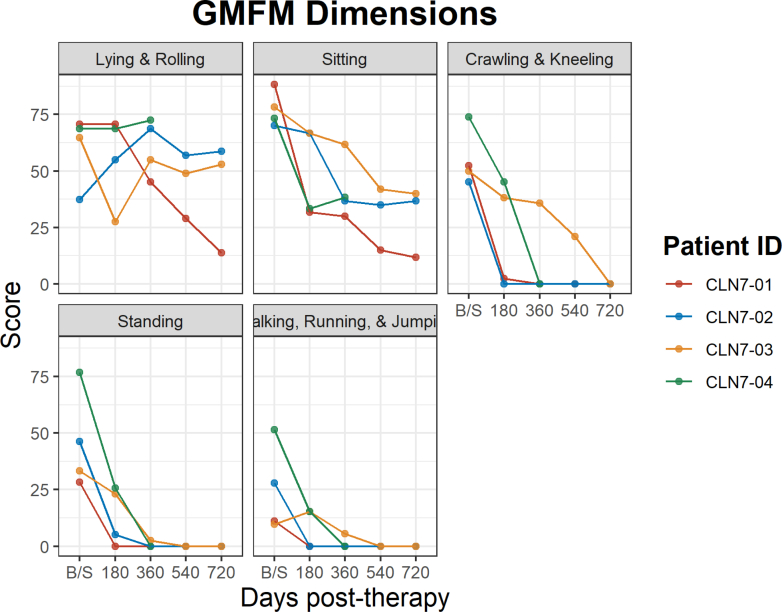
**The Gross Motor Function Measure (GMFM)**. Consists of five dimensions (lying and rolling, sitting, crawling, standing and walking) which includes 88 items. Over the course of the trial all participants experienced a decline in motor function as measured by the GMFM, except for the lying and rolling dimension which had evidence of stable scores in 3 out of 4 participants. CLN-01 received the smaller dose of 5 x 10^14^ vg. The rest received the higher dose of 1 x 10^15^ vg. CLN-04 lacks data for data 540 and 720 as the family wished to avoid travel to the study site and invasive procedures.

The Gross Motor Function Measure (GMFM) was administered to capture changes in motor function. Over the course of the trial all participants experienced decline in motor function as measured by the GMFM, except for the lying and rolling dimension which had evidence of stable scores in 3 out of 4 participants (Fig. 7).

## Discussion

This phase 1 study of AAV9/CLN7 gene therapy explored the highest intrathecally-delivered dose of viral vector for a human participant to date. This study reached its primary endpoint with a favourable safety and tolerability profile for such a dosing regimen, opening future trials to explore this dose and others in the pursuit of maximising transgene delivery to CNS cells. Additionally, this study identified important considerations relative to immunosuppression regimens for gene therapy and monitoring protocols.

Intrathecal high dose AAV9/MFSD8 showed a positive safety profile and is well tolerated in patients with CLN7.

First, relative to dose, prior gene therapy trials utilising AAV9 intrathecal delivery vectors have utilised doses below 5 x 10^14^ vg. This trial provided data for three participants with doses of 1 x 10^15^ vg, the highest intrathecal dose utilised in any gene therapy trial to date. It is worth noting that this lot of AAV9/CLN7 was comprised of 42% genome-containing particles, so the 1 x 10^15^ vg dose delivered an AAV9 capsid dose of 2.38 x 10^15^ particles. Prior to this study, concerns related to capsid mediated immune toxicity limited dose ceilings. This trial utilised a more aggressive immunosuppression regimen to determine if high dose intrathecal therapy would be tolerated. The lack of early anti-capsid immune mediated toxicity and the lack of DRG toxicity suggest that higher doses of gene therapy can be considered in the future. Specifically, regarding the DRG toxicity, recent studies suggest this is due to transgene overexpression,^30^ so use of the relatively weak JeT promoter may have contributed to the safety of AAV9/CLN7. Increased dosing is critical for a therapy that likely can only be dosed once.

Second, relative to the immunosuppression regimen our protocol called for a combination of corticosteroids with sirolimus, or corticosteroids combined with both sirolimus and tacrolimus. The use of the two regimens was for participants with CRIM positive status, based on an assessment of the patient's MFSD8 gene mutations that would predict they make some amount of full-length MFSD8 protein. CRIM negative recipients would require additional immune suppression to prevent an immune response to the newly expressed transgene. In our study, 3 of 4 participants were predicted to be CRIM negative and were managed with three immunosuppressant medications. Over the course of the trial, none of these participants had evidence of an immune response to the transgene based on serial MRIs, CSF analysis and ELISpots. The single participant predicted to be CRIM positive (patient 2), who was managed with only two immunosuppressants, experienced a clinically asymptomatic CSF pleocytosis after weaning off corticosteroids at six months post-injection. This pleocytosis resolved with reinstitution of steroids and did not appear to alter anti-AAV9 capsid humoral or T-cell–mediated immune responses observed from ELISpot analysis. The parent reportedly did not strictly adhere to the prescribed steroid tapering protocol, and the participant experienced multiple viral infections during this period.

After 24 months, all participants were enrolled in a 5-year long-term follow-up protocol including optional weaning from sirolimus with safety assessments including MRI, lumbar punctures, and laboratory tests conducted. No immunological reactions were observed thus far in participants that discontinued sirolimus, and participants have remained clinically stable over subsequent follow up. These findings contribute to our understanding of safely tapering paediatric patients from immunomodulatory drugs in CRIM-negative cases and provide valuable insights into immune suppression protocols for intracerebral gene therapy delivery.

### AAV9/MFSD8 IT delivery demonstrated some disease modification in patients with CLN7

The typical presentation of CLN7 disease is a uniformly fatal neurodegenerative condition that follows a course of steady decline. Participants in this trial demonstrated some stabilisation among certain measures, but continued declines in others. It is notable that after two years of follow up all of the participants are between 6 and 8 years of age and have maintained their respiratory function. In addition, at the end of initial two year-monitoring period, all the participants maintained oral feedings, some with modified diet, and did not require G button surgery. This is of significance as most of the patients in advanced stages of CLN7 disease will require G button feeds to sustain the daily caloric and nutritional needs around 8–10 years of age.

Three out of four families consistently reported a subjective improvement in myoclonic seizures with good seizure control on anti-seizure medication regimen. At the end of the two-year monitoring period, one participant was on monotherapy with levetiracetam, one was stable on levetiracetam and clobazam, one was stable on levetiracetam, Clobazam and lamotrigine. The fourth participant who struggled with seizure control was also found to have multiple viral illnesses in the first six months post therapy, which might have contributed to difficult seizure control. It is noteworthy that patients with CLN7, as described in literature and reported above, typically have seizure onset at an average age of 4.5 years, and by 6.5 years of age with progressive disease, seizures become intractable and are difficult to control with anti-seizure medications. It is possible that the single dose of AAV9 vector transduces cells in a regional manner throughout the brain causing improvement in certain populations of neurons while others continue to degenerate. This possibility underscores the need to find ways to increase the dosage of vector used and identify immunosuppression regimens that may allow for repeat dosing. Alternatively, it is also possible that there are regional differences in the rate of neurodegeneration, such that some functions were already beyond the capacity for rescue when the participants were treated. The latter supports a noncontroversial assumption supported by the preclinical studies^20^, that the timing of the intervention is likely to have a very large impact on the treatment efficacy and underscores the need to identify participants earlier in their disease course (ideally pre-symptomatically). This was the first study of AAV9 gene therapy to utilise an intrathecal dose of 1 x 10^15^ vg and it was shown to be safe and well tolerated within this protocol. Preclinical data indicates that the NOAEL exceeds the selected human dose by 3 times, suggesting a potential for dose escalation in future studies, which may improve efficacy outcomes.^20^ Further studies at this dose and higher doses should be considered. In addition, alternative routes like intracerebrovascular or intracisternal routes should be explored to assess potential improvements in brain bioavailability. Future studies are being planned focused on a Phase 3 trial to treat additional subjects and further evaluate the efficacy of the drug product. Observations from this study support efficacy evaluations for future studies centred around seizure burden, g-button dependency/swallow function tests and survival.

The study has several limitations, including a small sample size. Additionally, it is an open-label, non-randomised trial.

### Conclusions

This study explored the safety and preliminary efficacy of a high intrathecal dose of an AAV9 based gene therapy for CLN7 disease. It documented this dose, and the associated immunosuppression regimen was well tolerated overall. While overall efficacy signals were limited, there was some suggestion of a clinical impact. Future AAV9 based gene therapy studies should consider high doses and adequate immunosuppression to prevent adverse reactions to capsid protein or gene product.

## Contributors

BMG is the IND sponsor and contributed to study design, data collection, Formal analysis, manuscript creation and editing. BMG had full access to data and verified data.

BM contributed to the IND preparation, study design, data analysis and manuscript editing. BM had full access to data.

SM contributed to the IND preparation, study design, trial management, data collection, data analysis and manuscript preparation and editing. SM had full access to data.

VBE contributed to data collection, data analysis and manuscript editing.

AL contributed to data collection, data analysis and manuscript editing.

HD contributed to data analysis and manuscript editing.

ERN contributed to data collection, data analysis and manuscript editing.

HHN contributed to trial management, data collection and manuscript editing.

SH contributed to protocol development and the IND preparation.

ARM contributed to data analysis and manuscript editing.

KS contributed to data generation, data analysis and manuscript editing.

SI contributed to data collection, data analysis and manuscript editing.

GV contributed to the IND preparation, study design and manuscript editing.

SJG developed the AAV9/CLN7 vector, contributed to the IND preparation and study design, and edited the manuscript. SJG had full access to data.

SNK contributed to the IND preparation, study design, data collection, data analysis, manuscript preparation and editing. SNK had full access to data and verified data.

All authors read and approved the final version of the manuscript.

## Data sharing statement

Individual participant data that underlie the results reported in this article will be made available after de-identification. Information will be shared with researchers who provide a methodologically sound proposal to achieve the aims of the approved proposal. Proposals should be directed to benjamin.greenberg@utsouthwestern.edu; to gain access, data requestors will need to sign a data access agreement. Data will be shared directly with approved requestors.

## Declaration of interests

SJG is an inventor on the AAV9/CLN7 vector design (patent application publication # US-2021-0316012-A1) which is held by the University of North Carolina at Chapel Hill. Royalties were paid by Neurogene for a prior license and has received royalty income related to this invention from Neurogene. He is also named in a pending International Patent application PCT/US19/045911 for optimized CLN7 genes and expression cassettes and their use. He also serves on the Board of Directors of Elpida Therapeutics, and of the American Society of Gene and Cell Therapy (both unpaid). Dr. Messahel received served as a consultant to Citizen Health and Agastiya Biotechnology and participated on an Advisory Board for Chelsea's Hope. Dr. Bordes Edgar is co-investigator on grants from the Raynor Cerebellum Project, the Cure SPB50 Foundation, the NIH-NINDS, and Elpida Therapeutics (including receipt of consulting fees as a consultant for neuropsychological protocols and assessments across multiple study sites). Dr. Bordes Edgar also received honoraria for speaking presentations and/or travel support for the International Neuropsychological Society, the National Academy of Neuropsychology, and the Hispanic Neuropsychological Society. She participates on the Scientific Advisory Board for the National Institutes of Health Toolbox, the Pediatric Mental Health Care Access Advisory Council for the My Health My Resources Tarrant County, as a Scientific Research Council Member for Niños Health, and as a Board member for the American Academy of Neuropsychology, the Council of Representatives – American Psychological Association, and the Hispanic Neuropsychological Society. Dr. Muthukumar received a grant from Elpida Therapeutics.
