# Supplementary material for: First-in-human high dose AAV9 intrathecal gene therapy for paediatric CLN7 disease: a phase 1, open-label, single ascending dose, non-randomised clinical trial
Source: eBioMedicine. 2025 Nov 27;123:106044. doi: 10.1016/j.ebiom.2025.106044 (PMC12703863; doi:10.1016/j.ebiom.2025.106044)
Supplement: Supplementary Material 1 [file mmc1.docx]

**Supplementary Table 1: Line listing of all adverse events by AAV9/MFSD8:** Line listings for all adverse events (AEs) are documented for the four participants in the study (IDs CLN7-01 to CLN7-04). Most AEs were mild (Grade 1) and unrelated to the intervention, including issues such as muscle cramps, infections, and gastrointestinal symptoms. A few moderate (Grade 2) events, such as increased seizure frequency and nasal congestion, were reported. There were three notable Grade 3 events: pleocytosis in CLN7-02 (one of which was considered possibly related to the intervention) and a hospital admission for vomiting in CLN7-04, also classified possibly related to the intervention. Overall, the AEs were varied, but the majority were mild and not linked to the treatment. No Adverse events of special interest (AESIs) were reported.

| **Participant ID** | **Dose of AAV9/CLN7** | **Relationship to Intervention**  **(Y/N)** | **Grade** | **Expected**  **(Y/N)** | **Description** |
| --- | --- | --- | --- | --- | --- |
| CLN7-01 | 5E14vg | N | 1 | N | Laceration of face |
| CLN7-01 | 5E14vg | N | 1 | N | Bruising of leg |
| CLN7-01 | 5E14vg | N | 1 | N | QT shortening |
| CLN7-01 | 5E14vg | N | 1 | N | Muscle cramp |
| CLN7-01 | 5E14vg | N | 1 | N | Hair texture abnormal |
| CLN7-01 | 5E14vg | N | 1 | N | Hyperlipidaemia |
| CLN7-01 | 5E14vg | N | 1 | N | Bruising |
| CLN7-01 | 5E14vg | N | 1 | N | Neck muscle cramp |
| CLN7-01 | 5E14vg | N | 1 | N | Flatulence |
| CLN7-01 | 5E14vg | N | 1 | N | Urticaria |
| CLN7-01 | 5E14vg | N | 2 | N | ER visit due to Seizure |
| CLN7-01 | 5E14vg | N | 1 | N | Constipation |
| CLN7-01 | 5E14vg | N | 1 | N | Flatulence |
| CLN7-01 | 5E14vg | N | 1 | N | Pinworms |
| CLN7-01 | 5E14vg | N | 1 | Y | Oral Ulcers |
| CLN7-01 | 5E14vg | N | 1 | N | Viral Infection |
| CLN7-01 | 5E14vg | N | 1 | N | Dental Infection |
| CLN7-01 | 5E14vg | N | 1 | N | Constipation |
| CLN7-01 | 5E14vg | N | 1 | N | Choking |
| CLN7-01 | 5E14vg | N | 1 | Y | Mouth Blisters |
| CLN7-01 | 5E14vg | N | 1 | N | Tooth Extraction |
| CLN7-02 | 1E15vg | N | 1 | N | Constipation |
| CLN7-02 | 1E15vg | N | 1 | N | Laceration to lip |
| CLN7-02 | 1E15vg | N | 1 | N | Serum Amylase increase |
| CLN7-02 | 1E15vg | N | 1 | N | Muscle cramp in left leg |
| CLN7-02 | 1E15vg | N | 1 | Y | Hair texture abnormal |
| CLN7-02 | 1E15vg | N | 1 | N | Urine Infection |
| CLN7-02 | 1E15vg | N | 1 | N | Fever |
| CLN7-02 | 1E15vg | N | 1 | N | Increased Seizure Frequency |
| CLN7-02 | 1E15vg | N | 2 | N | Leg/foot pain |
| CLN7-02 | 1E15vg | N | 2 | N | Nasal Congestion |
| CLN7-02 | 1E15vg | N | 3 | Y | Pleocytosis |
| CLN7-02 | 1E15vg | N | 1 | N | Para-flu Infection |
| CLN7-02 | 1E15vg | N | 1 | N | Fever |
| CLN7-02 | 1E15vg | N | 1 | N | Vomiting |
| CLN7-02 | 1E15vg | N | 1 | N | Facial rash |
| CLN7-03 | 1E15vg | N | 1 | N | Rhinorrhoea |
| CLN7-03 | 1E15vg | N | 1 | N | Sore throat |
| CLN7-03 | 1E15vg | N | 1 | N | Constipation |
| CLN7-03 | 1E15vg | N | 1 | N | Nasal Congestion |
| CLN7-03 | 1E15vg | N | 1 | N | Leg/foot Pain |
| CLN7-03 | 1E15vg | N | 1 | N | Vitamin D deficiency |
| CLN7-03 | 1E15vg | N | 2 | N | Urine infection |
| CLN7-03 | 1E15vg | N | 1 | Y | AST Elevation |
| CLN7-03 | 1E15vg | N | 1 | Y | Low WBC |
| CLN7-03 | 1E15vg | N | 1 | Y | Low Platelet count |
| CLN7-04 | 1E15vg | Y | 3 | Y | Hospital Admission due to Vomiting |
| CLN7-04 | 1E15vg | N | 2 | N | Broken Clavicle |
| CLN7-04 | 1E15vg | N | 2 | N | Increased Seizure Frequency |
| CLN7-04 | 1E15vg | N | 2 | N | COVID infection |
| CLN7-04 | 1E15vg | N | 2 | N | Fever |
| CLN7-04 | 1E15vg | N | 2 | N | Increased Seizure Frequency |
| CLN7-04 | 1E15vg | N | 1 | N | Decreased Body Weight |
| CLN7-04 | 1E15vg | N | 1 | N | Leg Cramps |
| CLN7-04 | 1E15vg | N | 1 | N | Neck Discomfort |
| CLN7-04 | 1E15vg | N | 1 | N | Headache |
| CLN7-04 | 1E15vg | N | 1 | N | ST Elevation |

Supplementary figure 1: Lab data for *participants with CLN7 disease over the course of the clinical trial.* Lab data revealed stability of liver and kidney function. There was no evidence of cytopenias, bone marrow reactivity or microangiopathy.

Normal ranges for children are as follows: aspartate aminotransferase (AST), 10–40 U/L; alanine aminotransferase (ALT), 10–45 U/L; blood urea nitrogen (BUN), 5–18 mg/dL; serum creatinine, 0.2–0.7 mg/dL in infants, increasing to 0.4–1.0 mg/dL in adolescents; haemoglobin (Hb),11.5–14.5 g/dL; haematocrit (Hct), 35–45% ; white blood cell (WBC) count, 5.0–15.0 × 10⁹/L; and platelet count, 150–450 × 10⁹/L; prothrombin time (PT), 11–15 seconds; international normalized ratio (INR), 0.8–1.2; and activated partial thromboplastin time (aPTT), 25–40 seconds. Mild physiological variations may occur with growth, hydration status, and developmental stage.


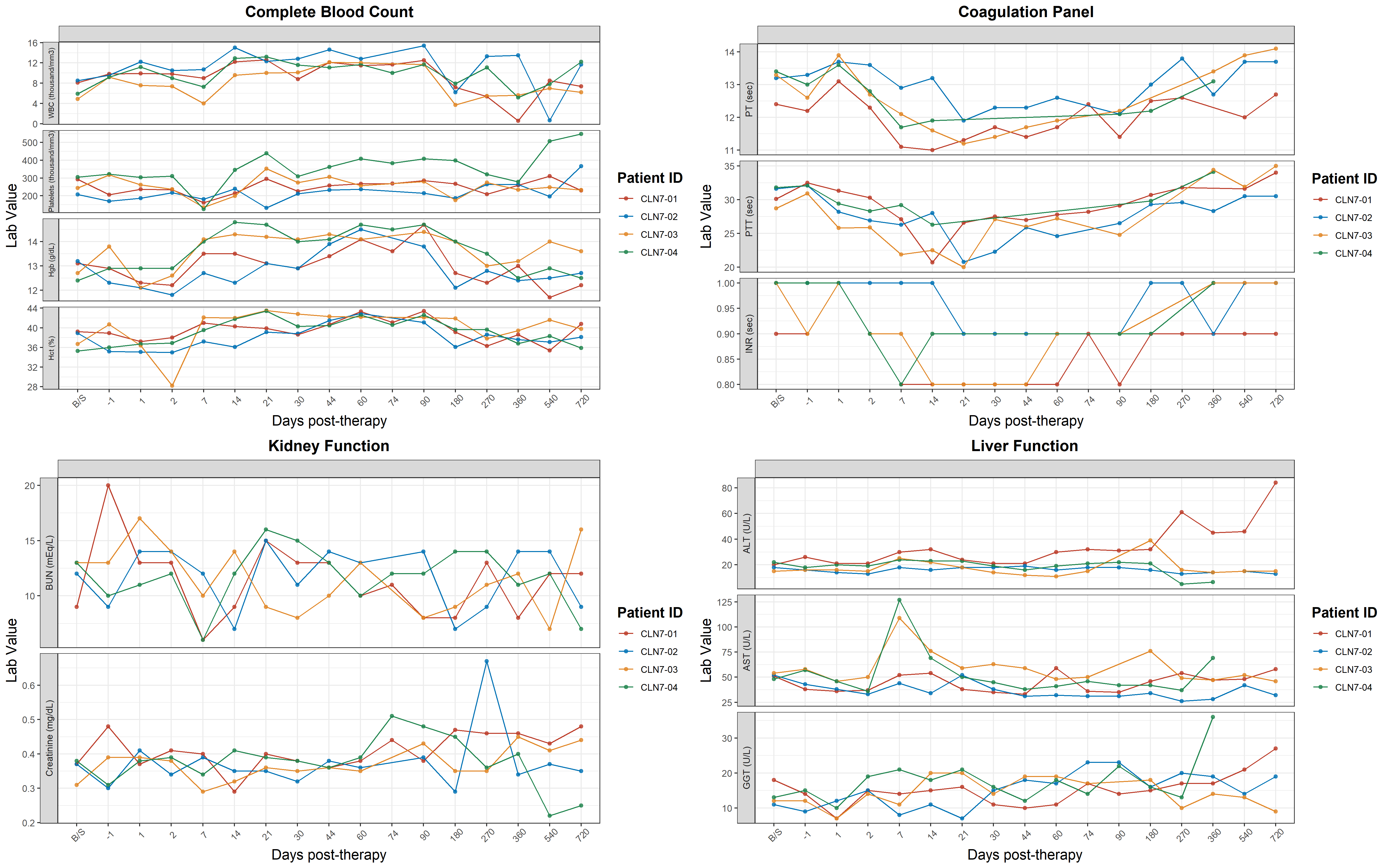


Supplementary figure 2: *Mullen Scales of Early Learning composite and T-scores in participants with CLN7 disease over the course of the clinical trial.* Mullen composite and individual T-scores for four participants. By Day 180, participants CLN7-01, CLN7-02, and CLN7-03 demonstrated scores at or near the lower limit (“floor”) of the instrument, and by Day 360, participant CLN7-04 had also reached floor performance. The resulting composite values are therefore not informative for quantitative comparison.


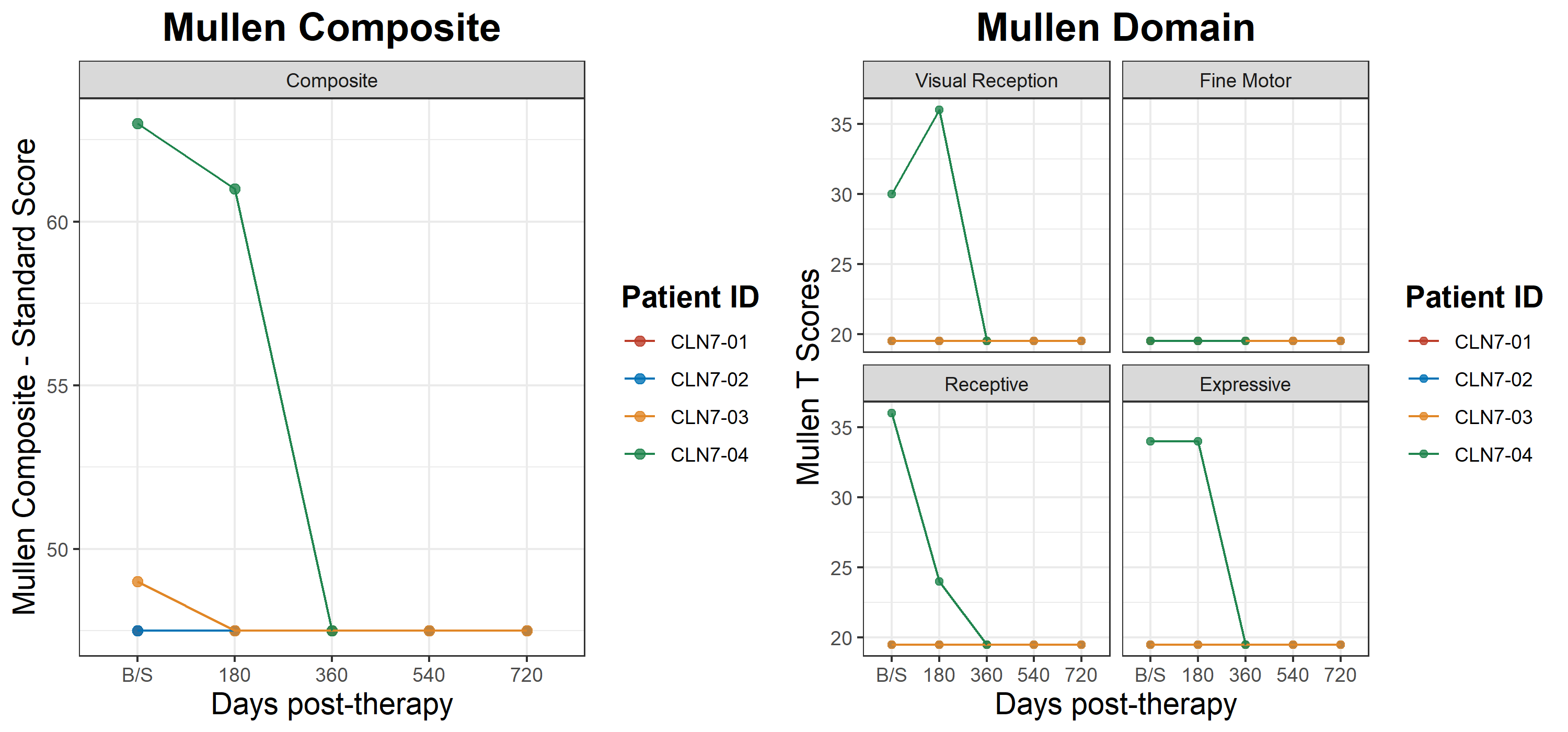


Supplementary figure 3: *The Vineland-3 Adaptive Behavior Scale Standard scores in participants with CLN7 disease over the course of the clinical trial*. Three participants had relative stabilization of their composite score between day 180 and 720. The greatest declines were seen in the areas of communication and motor skills and resulted in scores at the floor of the measure. Skills in the areas of daily living and social skills were generally more stable.


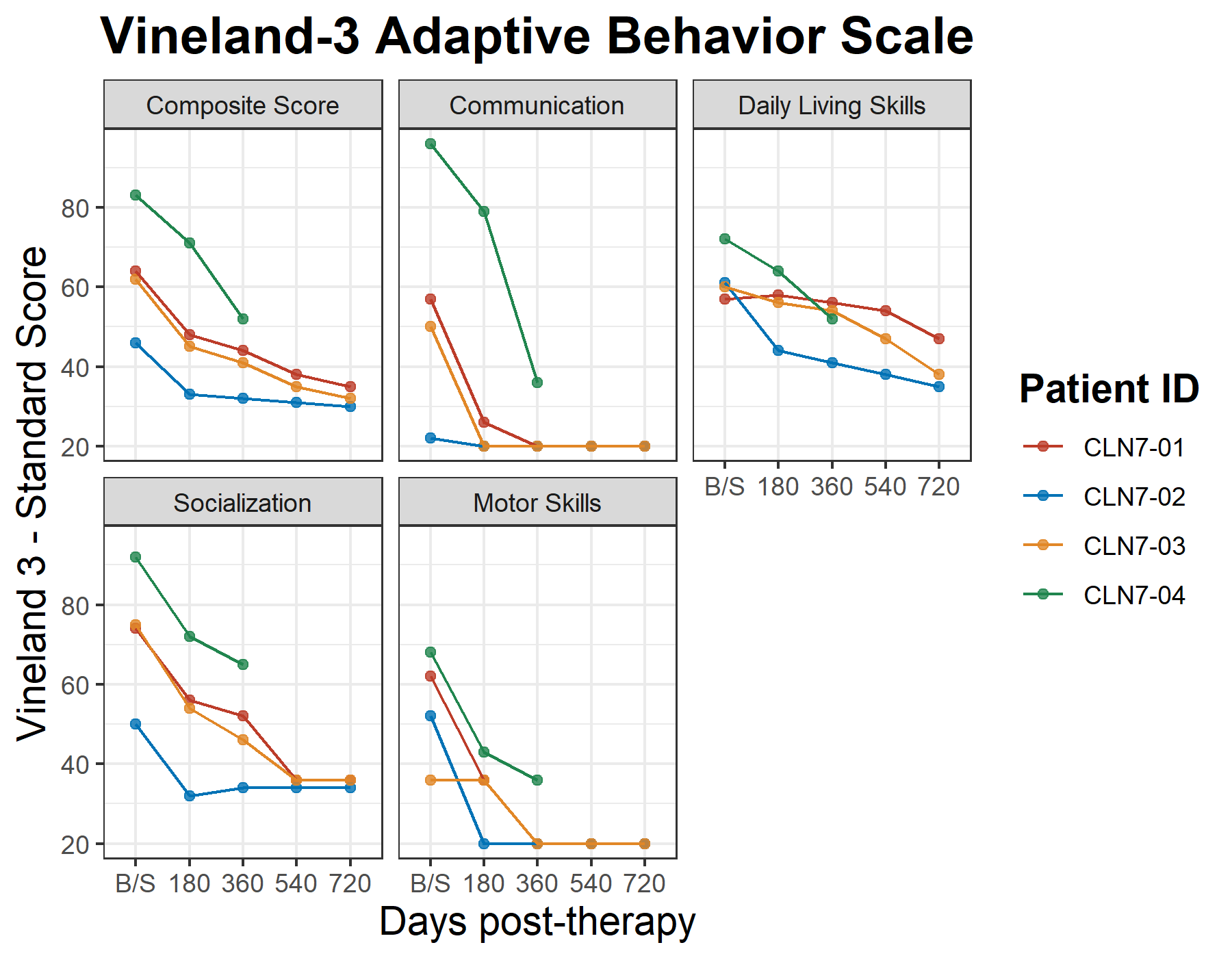


**Study Procedures and Methods**

After signing informed consent, participants completed required screening procedures to confirm eligibility.  Participants were admitted to the hospital the day before AAV9/CLN7 administration. Intrathecal infusion of AAV9/MFSD8 occurred using a volume of 10 mL administered over 10 minutes via lumbar puncture under anaesthesia. Participants were placed in Trendelenburg position (15 degrees, head down) for one hour after infusion and rotated from one side to the other 3 times over the hour.  After the procedure, participants were transferred to paediatric intensive care unit (PICU) for close monitoring with continuous pulse oximetry and telemetry for 24 hours followed by inpatient monitoring in a regular floor for another 24 hours prior to discharge with continued out-patient follow ups.

Sirolimus was started 7 days prior to the day of treatment, with a loading dose of 1mg/m^2^ every 4 hours for 3 doses followed by maintenance dose of 0.5 mg/m^2^/day divided into two doses with a goal serum level of 4-8 ng/ml. On the day of treatment, prior to the infusion, all participants were given loading dose of intra-venous methylprednisolone 10mg/kg, for a maximum dose of 500mg on the day of treatment prior to intrathecal vector administration. On day 1 post therapy a maintenance dose of enteral prednisone or prednisolone was started at 1mg/kg/day for 90 days.  For CRIM negative participants, Tacrolimus was started a day after vector administration at 0.1mg/kg/day with goal serum levels of 4-8 ng/ml.

Participants were monitored with frequent clinic visits, MRI brain, CSF analysis and bloodwork including ELISpot assays for immune responses to either AAV9 capsid peptides or MSFD8 derived peptides. If no evidence of an immune response was identified, participants would begin a prednisone taper after the day 90 visit. CRIM positive participants would begin a sirolimus taper after day 270 visit and labs were completed, while CRIM negative participants would begin a tacrolimus wean after 6-month post treatment. However, all participants in our study remained MRI brain, CSF analysis and bloodwork including ELISpot assays were completed at month 30 post therapy.

***Table of Abbreviations***

| **Abbreviation** | **Full Term** |
| --- | --- |
| 3T | 3 Tesla (a unit of magnetic field strength, often used for MRI machines) |
| Arg | Arginine |
| CLN7 | Ceroid Lipofuscinosis, Neuronal type 7 |
| CNS | Central Nervous System |
| DNA | Deoxyribonucleic Acid |
| EEG | Electroencephalogram |
| ID | Identifier |
| Ile | Isoleucine |
| IRB | Institutional Review Board |
| ITQOL | Infant Toddler Quality of Life Questionnaire |
| Leu | Leucine |
| Lys | Lysine |
| MFSD8 | Major Facilitator Superfamily Domain Containing 8 |
| MPRAGE | Magnetisation Prepared Rapid Gradient Echo |
| MRI | Magnetic Resonance Imaging |
| NCT | National Clinical Trial |
| QI-Disability | Quality of Life Inventory-Disability |
| SD | Standard Deviation |
| SS | Standard Score |
| T2 FLAIR | T2 Fluid-Attenuated Inversion Recovery |
| TE | Echo Time |
| Thr | Threonine |
| TI | Inversion Time |
| TR | Repetition Time |
| Trp | Tryptophan |
| USA | United States of America |
| Val | Valine |
