# Supplementary material for: First-in-human high dose AAV9 intrathecal gene therapy for paediatric CLN7 disease: a phase 1, open-label, single ascending dose, non-randomised clinical trial
Source: eBioMedicine. 2025 Nov 27;123:106044. doi: 10.1016/j.ebiom.2025.106044 (PMC12703863; doi:10.1016/j.ebiom.2025.106044)
Supplement: Supplementary Material 2 [file mmc2.pdf]

Phase I Intrathecal Lumbar Administration of AAV9/CLN7 for Treatment of CLN7 Disease

Authors: Benjamin Greenberg, MD, MHS; Saima Kayani, MD; Steven Gray, PhD

Date: September 2020

Version: 1.0

TABLE OF CONTENTS

LIST OF TABLES 4
1.0 Study Synopsis 5
2.0 Schedule of Events 11
3.0 Background of CLN7 11
4.0 Rationale for the Study 12
5.0 Study Objectives 13
5.1: Primary Objectives 13
5.2: Secondary Objectives 13
6.0 Investigational Plan 13
6.1: Study Design 13
6.2: Dose and Route 13
6.2.1: Dose Selection Rationale 14
6.2.1.1: Justification of clinical study dose 14
6.2.1.2: Rationale for brain size dependent dose calculation 14
6.2.2: Anesthesia Safety 14
6.2.3: Post-Procedure Recovery 15
6.3: Dosing Device for Phase I Clinical Trials 15
6.4: Endpoints 15
6.4.1: Safety 15
6.4.2: Efficacy 16
6.4.2.1: Disease Burden Assessments 16
6.4.2.1.1: Clinical Global Impression (CGI) 16
6.4.2.2: Neuropsychology Assessments 16
6.4.2.2.1: Mullen Scales of Early Learning (Mullen) 16
6.4.2.2.2: Vineland Adaptive Behavior Scales, 3rd Edition (Vineland-3) 17
6.4.2.3: Quality of Life Assessments 17
6.4.2.3.1: Quality of Life Inventory – Disability (QI-Disability) 17
6.4.2.3.2: Infant and Toddler Quality of Life Questionnaire – Short Form (ITQOL) 18
6.4.2.4: Ataxia and Motor Function Assessments 18
6.4.2.4.1: Timed Walk (Two Minute Walk Test or Six Minute Walk Test) 18
6.4.2.4.2: Pediatric Balance Scale 19
6.4.2.4.3: Gross Motor Function Measure (GMFM) 19
6.4.2.5: Surrogate Assessments 20
6.4.2.5.1: Electroencephalogram (EEG) 20
6.4.2.5.2: Magnetic Resonance Imaging of Brain (MRI) 20
6.4.2.6: Exploratory Assessments 20
6.4.2.6.1: Ophthalmologic Evaluation 20
6.4.2.6.2: Swallow Function Test 20
6.4.2.7: Diaries 20
6.4.2.7.1: Seizure Diary 20
6.4.2.7.2: Healthcare Utilization Diary 21
6.4.2.7.3: Telephone Adverse Event Monitoring 21
6.5: Duration of Study 21
6.6: External Data Monitoring 21
7.0 Selection of Participants 21
7.1: Inclusion Criteria 21
7.2: Exclusion Criteria 21
7.3: Duration of Inclusion of Participants 22
7.4: Participant Withdrawal Criteria 22
8.0 Treatment 22

8.1: Investigational Product ... 22
8.2: Packaging ... 22
8.3: Labeling and Storage ... 23
8.4: Dose Administration ... 23
8.5: Dose De-Escalation Plan ... 23
8.6: Immune Modulation Protocol ... 23
8.6.1: For participants with CRIM-pos status ... 24
8.6.2: For participants with CRIM-neg status ... 24
8.6.3: For Mutations with Predicted Residual Protein Function ... 25
8.6.4: For Null Mutations ... 25
9.0 Study Assessments ... 26
9.1: Screening/Baseline (Days -28 to -7) ... 26
9.2: Pre-Infusion Visit (Day -1) ... 27
9.3: Day of Gene Transfer (Day 0) ... 28
9.3.1: Gene Transfer Procedures ... 28
9.3.2: Dosing ... 28
9.3.3: Intrathecal Administration ... 29
9.3.4: Infusion Reactions ... 29
9.4: Post-Gene Transfer Monitoring ... 29
9.4.1: Dose De-Escalation Plan ... 29
9.4.2: Immunomodulation Taper Plan ... 30
9.4.2.1: Steroid Taper ... 30
9.4.2.1.1: For steroid dose below 60 mg ... 30
9.4.2.1.2: For steroid dose at or above 60 mg ... 30
9.4.2.2: Sirolimus Taper ... 31
9.4.2.3: Tacrolimus Taper ... 31
9.4.3: Early Inpatient Monitoring (up to 48 hours of AAV9/CLN7 dose) ... 31
9.4.4: Outpatient Follow-up Visits (Day 7, 30, 60, 90, 180, 270, 360, 540, and 720) ... 32
9.4.5: Additional Visits for Follow-up Lab Monitoring ... 33
9.4.6: Follow-up MRI Brain ... 33
9.4.7: Follow-up Lumbar Puncture ... 33
9.5: Long-Term Monitoring ... 33
9.6: Outcome Measures ... 34
9.6.1: Safety Measures ... 34
9.6.2: Secondary/Exploratory Efficacy Endpoints ... 35
9.6.3: Statistical Analysis ... 35
10.0 Data Monitoring ... 35
10.1: General Plan ... 35
10.2: Monitoring Entity ... 35
10.3: Plans for Assuring Participant Safety, Adverse Event Collection, and Reporting ... 36
10.4: Definitions ... 36
10.4.1: Adverse Event ... 36
10.4.2: Classification of Adverse Events ... 36
10.4.3: Seriousness ... 36
10.4.4: Expectedness ... 37
10.4.5: Causality ... 37
10.4.6: Dose Limiting Toxicity ... 38
10.4.7: Other Adverse Events ... 38
10.5: Reporting Procedures to the DSMB ... 38
10.6: Reporting Procedures to the FDA ... 39
10.7: Reporting Procedures to the IRB ... 40
10.8: Protocol Deviations and Continuing Review ... 40

10.9: Stopping Rules 40
10.10: Data Collection 41
10.10.1: Database Locks 42
10.10.2: Study Monitoring Plan 42
10.10.3: Quality Assurance of Data 42
11.0 References 47

LIST OF TABLES

Table 1: Secondary Outcome Assessments 10
Table 2: Relationship Between Preclinical Study Doses and Proposed Human Intrathecal Dose 14
Table 3: Dose Extrapolation Based on Age and Brain Size 14
Table 4: Neuropsychology Assessments 16
Table 5: Quality of Life Assessments 17
Table 6: Ataxia and Motor Function Assessments 18
Table 7: Five Year Follow-Up (Period B) 34
Table 8: Complete Schedule of Events (Period A) 43

1.0 Study Synopsis

**Sponsor:**	Benjamin M. Greenberg, MD, MHS
**Active Ingredient:**	AAV9/CLN7
**Study Title:**	Phase I Intrathecal Lumbar Administration of AAV9/CLN7 for Treatment of CLN7 Disease
**Study Phase:**	Phase I
**Study Site:**	University of Texas Southwestern Medical Center / Children's Health – Children's Medical Center in Dallas, Texas
**Study Lead**	**PI:** Benjamin Greenberg, MD; **Co-I:** Saima Kayani, MD; Berge Minassian, MD;<br>Steven Gray, PhD
**Objectives:**	
**Primary Objective:**	The primary objective of this study is to evaluate the safety and tolerability of single doses of AAV9/CLN7 administered intrathecally to children with CLN7 disease by the incidence and severity of treatment related serious adverse events (SAEs).
**Secondary Objectives:**	The secondary outcomes will be measures of efficacy of the drug as seen by improved disease burden, motor function, neuropsychological, and quality of life assessments to include the following:<br><br>**Disease Burden Assessments**:<br>1. Clinical Global Impression Scale (CGI)<br><br>**Ataxia and Motor Function Assessments:**<br>1. Timed Walk tests (2-Minute Walk Test OR 6-Minute Walk Test)<br>2. Pediatric Balance Scale<br>3. Gross Motor Function Measure (GMFM)<br><br>**Neuropsychological Assessments:**<br>1. Mullen Scales of Early Learning<br>2. Vineland Adaptive Behavior Scales, 3rd Edition<br><br>**Quality of Life Measures;**<br>1. Quality of Life Inventory-Disability (QI-Disability)<br>2. Infant/Toddler Quality of Life Questionnaire (ITQOL)<br><br>**Surrogate Measures:**<br>1. Electroencephalogram (EEG)<br>2. MRI brain<br><br>**Exploratory Measures:**<br>1. Optic Nerve Pathology<br>2. Visual Acuity (VA)

3. Electroretinography (ERG)
4. Optical Coherence Tomography (OCT)

Methodology

Study Design

This will be a first-in-human Phase I, open-label, single dose clinical study of AAV9/CLN7 administered intrathecally (IT) through a lumbar puncture (LP) in participants with confirmed pathogenic or likely pathogenic mutations in the *MFSD8* gene. Participants will be symptomatic with clinical signs/symptoms of CLN7 disease or patients with the pathogenic mutation who are still asymptomatic.

Study Procedures

Following obtaining informed consent from the participant/parent/guardian, the participant will be evaluated for inclusion and exclusion criteria and those meeting the requirements will be enrolled. All participants/parents/guardians will provide a written consent or assent before any study procedures are performed. Participants/parents/guardians will be informed that participation in the study is voluntary and they can withdraw at any time without any ramifications on their clinical care. Participants will have a spinal needle inserted percutaneously at the lumbar level into the intrathecal space of the spinal column with placement verified via imaging. A volume of cerebrospinal fluid (CSF) approximately equal to the infusion volume will be withdrawn from the lumbar thecal sac. A solution of the agent at a dose of 1x1015 vector genomes (vg) (or the low dose of 5x1014vg) will then be infused at a rate of one (1) mL per minute by infusion pump. This concentration is calculated based on pre-clinical data and is the maximum dose that is expected to be safely tolerated (Table 2). The participant will remain in the Trendelenburg position at 15 degrees (head down) for one (1) hour following vector administration, during which time the patient will be turned every 15 minutes. The procedure will be performed in a procedure unit with an anesthesiologist or qualified physician present to administer sedation as needed. Participants will stay in the Pediatric Intensive Care Unit (PICU) overnight. Physiologic monitoring in accordance with the standards set by the American Society of Anesthesiologists will be utilized for all participants while they are receiving analgesia/anesthesia and until they have fully recovered from its effects. Vital signs including blood pressure (BP), heart rate (HR), respiration rate (RR), temperature (T), oxygen saturation (O2 sat), and heart rhythm (via telemetry) and daily electrocardiogram (ECG) will be checked frequently while hospitalized after the procedure. A tapering course of prophylactic enteral prednisone/prednisolone and sirolimus will be administered. In addition, tacrolimus will be used for participants who are predicted to have null mutations with no residual protein function and are labelled as cross-reactive immunological material negative (CRIM-neg).

Participants will have outpatient clinic follow-up visits on Day 7, 30, 60, 90, 180, 270, 360, 540, and 720. There will be further follow-up lab visits (in addition to the above-mentioned visits) weekly in the first month and biweekly during the second and third months after dosing. During outpatient clinic visits, participants will undergo physical and neurologic exams with vital signs. Various clinical assessments to include disease burden assessments, motor, and neuropsychological tests will be done at baseline and at 6-month intervals after the procedure.

ECG will be done on Days -28 to -7, -1, 0, 1, 2, 7, 14, 21, 30, 60, 90, 360, and 720. Additional ECG with cardiology consult may be required if any abnormality is detected on ECG.

Repeat magnetic resonance imaging of the brain with or without sedation (MRI brain with or without contrast) will be completed on days 90, 180, 360, 540 and 720 and LP (also with or without anesthesia) will be completed for safety assessments on days 90, 180, 360 and 720. See Table 8 for a full visit schedule. Data obtained through MRI brain might also be used as an exploratory efficacy outcome measure.

Ophthalmologic assessments to include a dilated eye exam, electroretinogram (ERG), and when patients are clinically able, ocular coherence tomography (OCT) and visual acuity (VA) will be completed. They will be done at baseline and at 12-month intervals. Ophthalmologic assessments might serve as an internal control specifically for participants who are pre-symptomatic or are in early stages of the disease.

A swallow study will be done at baseline and at 12-month intervals from baseline for 24 months.

COVID-19 testing will be performed per hospital and institutional policies.

Please note that any new unanticipated medical or neurologic signs and symptoms might need extended monitoring and intervention during the inpatient stay, or might result in additional clinical or lab visits at any point during study follow-up.

Inclusion Criteria

- 1-18 years of age
- Clinically symptomatic patients with diagnosis of CLN7 based upon molecular testing with homozygous or compound heterozygous and pathogenic or likely pathogenic mutations in *MFSD8* gene with symptom onset before age 4
- Clinically pre-symptomatic patients with molecularly confirmed diagnosis of CLN7 with homozygous or compound heterozygous pathogenic mutations in *MFSD8* gene less than or equal to 4 years of age
- Written informed consent provided by participant/parent/guardian and willingness to participate and comply with all the study related visits and procedures. Assent provided by children 10 -17 years old based on their ability to understand the risks and possible benefits, and the activities expected of them as participants.

Exclusion Criteria

- Diagnosis of a second neurodegenerative disease or another genetic syndrome with a progressive course
- Hypersensitivity to any drugs used per procedural protocol
- Inability to tolerate anesthesia or study procedures
- Advanced stage disease defined by the use of chronic invasive ventilatory support (tracheostomy with ventilator dependence) and a non communicative status
- Concomitant illness that places patient at risk for gene transfer or gene transfer related procedures and immunosuppression
- Active, symptomatic viral infection (including but not limited to HIV or serology positive for Hepatitis B or C, or COVID-19) at the PI’s discretion
- Family is unwilling or unable to participate with required follow-up assessments
- Abnormal lab values that are clinically significant:
    - Platelet count < 100,000/mm^3
    - Abnormal absolute neutrophil count (ANC) of < 1000/mm^3

  - Persistent leukopenia or leukocytosis (Total white blood cell count < 3,000/mm or > 15,000/mm respectively)
  - Significant anemia (Hb <10 g/dL)
  - Abnormal prothrombin (PT) or partial thromboplastin time (PTT)
  - Abnormal liver function tests (>2 X ULN or > 2 X the baseline value at time of dosing)
  - Abnormal pancreatic enzymes (>2 X ULN or > 2 X the baseline value at time of dosing)
  - Renal impairment defined as urinary protein concentration greater than or equal to 0.2 g/L on 2 consecutive tests
  - Any other abnormal lab values that are clinically significant per PI's discretion
  - If labs are abnormal, these can be rechecked during the screening period. If labs normalize with or without intervention, patient can be enrolled at the discretion of PI.
- Contraindications for intrathecal administration of the product via lumbar puncture, such as bleeding disorders or other medical conditions (e.g., spina bifida or clotting abnormalities)
- Contraindications for MRI scans (including but not limited to cardiac pacemaker, metal in the eye, aneurysm clip in the brain, etc.)
- History of or current chemotherapy, radiotherapy, or other immunosuppression therapy within 30 days preceding screening (corticosteroid treatment may be permitted at the discretion of the PI)
- Receipt of any other investigational agent within the previous 3 months
- Positive Beta hCG pregnancy test (females of child bearing potential will have a pregnancy test on Day -1)
- Any other medical condition that puts the subject at increased risk of adverse events related to the investigational product or study-related procedures.

Investigational Product Safety and Dose Rationale

Dosing Procedure for Phase I Clinical Trials:
The study agent, vials of AAV9/CLN7, will be formulated as a concentrated stock in phosphate-buffered saline containing 5% D-sorbitol and 0.001% pluronic F68, and stored at ≤ -80˚C until the day of agent administration. The solution will be thawed within 4 hours of administration and diluted to the appropriate final dosage concentration and volume using phosphate-buffered saline with 5% D-sorbitol and 0.001% pluronic F68 (if necessary). The first participant will receive a low dose of $5X10^{14}$ vg and following a safety and tolerability evaluation, subsequent participants will receive a higher dose of $1x10^{15}$ vg of the AAV9/CLN7 agent. Participants below the age of 4 years will receive a dose calculated on the basis of their brain volume. This dose of the agent is calculated on the basis of pre-clinical studies and is expected to be the maximum dose that is tolerable (Table 2).

Anesthesia Safety:
Physiologic monitoring in accordance to the standards set by the American Society of Anesthesiologists will be utilized for all participants while they are receiving analgesia/anesthesia and until they have fully recovered from its effects.

Recovery:
After the procedure, participants will be transported to a Post-Anesthesia Care Unit (PACU) or PICU based on clinician assessment and bed availability with continuous pulse oximetry monitoring and oxygen if needed by bag/mask/nasal canula or blow-by. Participants will be

	put on telemetry. Vital signs including heart rate, respiratory rate, blood pressure, and pulse oximetry will be monitored every 15 minutes for the first 2 hours post infusion, every 30 minutes during the third and fourth hour post infusion, then hourly for 4 hours, and finally every 4 hours until discharge.
**Data Collection and Schedule**	Participants will be screened for inclusion/exclusion criteria and those meeting the requirements will be enrolled. All participants/parents/guardians will provide a written consent or assent before any study procedures are performed. Participants/ parents/guardians will be informed that participation in the study is voluntary and they can withdraw at any time without any ramifications on their clinical care. No study procedures will occur prior to consent/assent. Participants will be admitted on Day -1 to the hospital. AAV9/CLN7 will be administered on Day 0 and safety will be monitored in 2 parts, Period A and Period B. During Period A, participants will be tested at screening/baseline (-28 to -7 days) and return for follow-up visits on Days 7, 30, 60, 90, 180, 270, 360, 540, and 720 for safety and efficacy assessments (complete visit schedule in Table 8). In Period B, subjects will be assessed on annual visits for five additional years after completion of Period A for long-term safety and efficacy assessments. During Period B, in between annual visits, participants/parents/guardians will be encouraged to contact the investigator for any suspected adverse event reporting between visits. Unscheduled visits may occur if the PI determines that they are necessary to assess safety, repeat labs, etc. Because practical and ethical concerns preclude contemporaneous or untreated control participants, experimental data will be compared with historical data from existing CLN7 registries, natural history studies, and baseline.<br>Study eligibility will be re-confirmed before participants are admitted to the hospital on Day -1 before the scheduled administration of AAV9/CLN7 (see Table 8 for a full visit schedule).
**Study Population**	This is a single-administration study with a planned cohort size of four (4) participants. The number of participants is selected based on the amount of product available currently for the study. Some participants will be recruited from the pool of patients followed at Children's Health. We will also consider patients referred by the family foundation and/or ClinicalTrials.gov. In addition to symptomatic patients, we will also recruit patients who are not yet symptomatic but have mutation(s) that cause the disease. This is a rare disorder affecting only a few known people in the United States, therefore no information on the demographic distribution is available. We will, however, make every effort to ensure a diverse pool of participants is recruited.
**Number of Participants**	Four (4)
**Study Duration**	The total study duration is seven (7) years for each participant. Initial screening and evaluation will be performed at -28 to -7 days before the administration of AAV9/CLN7. We will evaluate short-term safety over a two-year period. Participants will be tested at baseline (-28 to -7 days) and return for follow-up visits on days 7, 30, 60, 90, 180, 270, 360, 540, and 720 days for active monitoring (Period A). After the 24-month visit, they will be followed according to an annual monitoring plan for five (5) years after completion of Period A (Period B).

Outcome Assessments:

Safety

The primary outcome is safety.

A Data Safety Monitoring Board (DSMB) is appointed and will review all relevant safety data from the first participant at Day 30, before a second participant is dosed. Similarly, the third participant will not be dosed until safety on the second patient has been reviewed by the DSMB at 30 days post-injection.

Thirty days following the dosing of the third patient, data will be reviewed by the DSMB to determine if any further participants will be enrolled utilizing the same dose, or a lower dose, according to the conditional dose de-escalation plan.

Monitoring and follow-up for safety and efficacy will be done in two parts, referred to as Period A and Period B in this document. Period A will include safety and short-term efficacy assessments. In Period A, participants will be tested at screening/baseline (-28 to -7 days), early inpatient monitoring for 24 hours after AAV9/CLN7 dosing, and return for outpatient follow-up visits on Days 7, 30, 60, 90, 180, 270, 360, 540, and 720. In addition, there will be lab visits every week during the first month and every other week during the second and third month after dosing (see Table 8 for complete visit schedule). Participants will also undergo a MRI brain with or without contrast on Days 90, 180, 360, 540 and 720 and a LP with CSF analysis as safety assessments on Days 90, 180, 360, and 720. In Period B, participants will be assessed at annual visits for five (5) years after completion of Period A for long-term safety, as per FDA guidance for gene therapy studies, and efficacy assessments. During this period in between annual visits, participants/parents/guardians will be encouraged to contact the study team for any suspected adverse event reporting. Unscheduled visits may occur if the PI determines they are necessary to assess safety, repeat labs, etc.

Efficacy

The secondary outcome is efficacy as measured by the following cognitive, motor function, and quality of life assessments:

Table 1: Secondary Outcome Assessments

Purpose	Assessments	Frequency
Disease Burden Scale	1. Clinical Global Impression	Days -28 to -7, 180, 360, 540, 720
Motor Function Assessments	1. 2-Minute Walk Test OR 6-Minute Walk Test<br>2. Pediatric Balance scale<br>3. Gross Motor Function Measure (GMFM)	Days -28 to -7, 180, 360, 540, 720
Intelligence and Cognition Assessments	1. Mullen Scales of Early Learning<br>2. Vineland Adaptive Behavior Scales, 3rd Edition	Days -28 to -7, 180, 360, 540, 720

	Quality of Life Measurements	1. Quality of Life Inventory-Disability (QI-Disability)<br>2. Infant/Toddler Quality of Life Questionnaire (ITQOL)	Days -28 to -7, 180, 360, 540, 720
	Surrogate Measures	1. EEG<br>2. MRI Brain	1. Days -28 to -7, 180, 360, 540, 720<br>2. Days -28 to -7, 90, 180, 360, 540, 720 (MRIs will also serve as safety measures.)
**Safety Review and Stopping Criteria**	Please refer to Section 10 of this document.		

2.0 Schedule of Events

Participants will be screened for inclusion/exclusion criteria and those meeting the requirements will be enrolled. All participants/parents/guardians will provide a written consent or assent before any study procedures are performed. Participants/parents/guardians will be informed that participation in the study is voluntary and they can withdraw at any time without any ramifications on their clinical care. No study procedures will be occur prior to consent/assent. Participants will be admitted on Day -1 to the hospital. AAV9/CLN7 will be administered on Day 0 and safety will be monitored in 2 parts, Period A and Period B. During Period A, participants will be tested at screening/baseline (-28 to -7 days) and return for follow-up visits on Days 7, 30, 60, 90, 180, 270, 360, 540, and 720 for safety and efficacy assessments (complete visit schedule in Table 8). In Period B, subjects will be assessed on annual visits for five additional years after completion of Period A for long-term safety and efficacy assessments. During Period B, in between annual visits, participants/parents/guardians will be encouraged to contact the investigator for any suspected adverse event reporting between visits. Unscheduled visits may occur if the PI determines that they are necessary to assess safety, repeat labs, etc.

A complete schedule of events for the first 24 months monitoring (Period A) is found in Table 8 at the end of this document.

3.0 Background of CLN7

CLN7 is caused by homozygous or bi-allelic heterozygous variants in CLN7/MFSD8 gene [1]. Siintola et al (2007) identified the *CLN7/MFSD8* gene in chromosome 4q28.1-28.2. *MFSD8* encodes a 518-amino acid polytopic protein which is expressed ubiquitously and belongs to a major facilitator superfamily of transmembrane proteins [1]. This lysosomal transmembrane protein has 12 membrane-spanning domains [1]. Since the initial identification of a mutation in the gene in 2007, a total of 38 different *MFSD8* mutations and 2 sequence variations have been reported in populations throughout the world [2, 3, 4]. The types of mutations include missense, splice site, nonsense, frame shift, sequence deletion or insertion. The autosomal recessive condition in children is inherited from healthy carrier parents, each contributing a defective copy.

The clinical severity varies from a mild, late-onset with non-syndromic visual deficits [5] to a severe, early-onset version that manifests as neurological signs with progressive deterioration in intellectual and motor capabilities, seizures, muscle spasms, and visual deficits culminating in premature death [2, 3, 6, 7]. Kousi et al [3] described the clinical phenotype of 25 patients of which 24 presented with the typical phenotype of variant late infantile neuronal ceroid lipofuscinosis (NCL). The mean age of onset was 3.3 years with an age range of 1.5-5 years. Mean age of death was 11.5 years. These patients were noted to have rapid deterioration in motor, language, and cognitive function after the diagnosis was made.

There are no proven therapies for CLN7. Current standard of care is based upon supportive care. The treatment approach is symptomatic as these may be different in various individuals with CLN7. These patients need a multidisciplinary approach with various sub-specialists including, but not limited to, pediatricians, pediatric neurologists, geneticists, cardiologists, physical medicine and rehabilitation specialists, dieticians and gastroenterologists.

4.0 Rationale for the Study

Since CLN7 is caused by monogenic mutations, gene replacement therapy could be a good choice to treat the disease. AAV9/CLN7 is a recombinant serotype 9 adeno-associated virus (AAV) encoding a codon-optimized human *CLN7* transgene. The final product consists of AAV9 capsids that are packaged with the self-complementary AAV genome comprising a mutant AAV2 inverted terminal repeat (ITR) with the D element deleted, the “JeT” promoter [8], codon-optimized human CLN7 deoxyribonucleic acid (DNA) coding sequence, the simian virus 40 polyadenylation signal, and wild-type AAV2 ITR. As a gene therapy, AAV9/CLN7 is expected to provide a fully functional human *MFSD8* cDNA copy to participant’s neuronal cells, thus reducing neurodegeneration. Given the urgent and severe unmet need in CLN7 disease and observations of safety in animals and efficacy in *in vitro* studies, clinical development of CLN7 is justified.

AAV9/CLN7 is an AAV9-based gene therapy vector that expresses the fully functional form of MFSD8 under the control of a synthetic promoter. AAV9/CLN7 will be delivered intrathecally and is designed to achieve stable, potentially life-long expression of MFSD8 in non-dividing cells. This clinical study is a first-in-human study designed to assess safety and tolerability of AAV9/CLN7 in CLN7 participants, as well as the impact of the gene therapy on disease progression.

Numerous investigators have utilized recombinant AAV9 directed at the central nervous system (CNS) in ongoing human clinical trials for gene therapy (clinical trial.gov identifiers NCT02122952, NCT02362438, NCT02725580, NCT02716246, NCT03315182). These vectors are non-pathogenic, non-replicating, and transduce non-dividing cells. However, the recombinant vectors are incapable of coding viral proteins or actively integrating with the host genome making them an ideal vector currently available for gene delivery. Additionally, AAV9 can be purified in large quantities at high concentrations for potential use in delivering a functional copy of a gene to cells with aberrant, disease causing mutations. In disorders of neurologic origin, targeted CNS-focused administration achieves broad transgene distribution in the CNS. An approach utilizing intrathecal (IT) administration of AAV9 was first advanced as a treatment of Giant Axonal Neuropathy (GAN). The laboratory of Dr. Steven Gray, in partnership with Hannah’s Hope Fund, initiated a first-in-human Phase I gene therapy clinical trial for GAN, in collaboration with Dr. Carsten Bonnemann at the US National Institutes of Health Clinical Center (NCT02362438) in 2015.

As CLN7 is a progressive neurodegenerative disease with onset of symptoms around two years of age and a shortened life span, we believe that the enrollment of adults in this clinical trial is not feasible. This is because there is a limited number of patients that can be enrolled and a limited number of adult patients with advanced disease, which will be prohibitive from a logistical standpoint at the performance site. In addition, if patients with milder disease were enrolled, that would diminish our capacity of measuring meaningful clinical outcomes due to the absence of a control cohort.

Based on our pre-clinical data, we expect that inclusion of pre-symptomatic patients will increase the probability of demonstrating clinical benefit. This subset of patients will have known pathogenic mutations in the *MFSD8* gene which is predicted to result in a typical clinical phenotype seen in variant late infantile NCL with a neurodegenerative course. This will include, but not be limited to, the siblings of diagnosed patients who have a predicted neurodegenerative course.

5.0 Study Objectives

5.1: Primary Objectives

The primary objective of this study is to evaluate the safety and tolerability of single doses of AAV9/CLN7 administered intrathecally to children with CLN7 disease by the incidence and severity of treatment related serious adverse events (SAEs).

5.2: Secondary Objectives

The secondary objectives will be efficacy of the drug. Participants will undergo motor function, neuropsychological, and disease burden assessments every 6 months starting at screening/baseline for up to 24 months. These assessments are summarized in the Table 1 and explained in section 6.4.2.

6.0 Investigational Plan

6.1: Study Design

This will be a first-in-human Phase I, open-label, single dose clinical study of AAV9/CLN7 administered intrathecally (IT) through a lumbar puncture (LP) in participants with confirmed mutations in the *MFSD8* gene. The first three participants will be symptomatic. After safety data is analysed from the third subject at 30-day post injection, pre-symptomatic patients may be enrolled in the study.

6.2: Dose and Route

All participants will have a spinal needle inserted percutaneously at the lumbar level into the intrathecal space of the spinal column. A volume of CSF approximately equal to the infusion volume is withdrawn from the lumbar thecal sac. The vector solution is then infused at a rate of 1 mL per minute for a total of 10 mL for participants 4 years of age and older (see Table 3 for volume adjustments for younger participants). The participant will remain sidelying in Trendelenburg position (head down) at 15 degrees for one (1) hour following agent administration, during which time the patient will be turned every 15 minutes. Dosing volumes will be calculated per Table 2 in section 6.2.1, depending on final vector product concentration. The procedure will be performed in a procedure unit with an anesthesiologist or qualified physician present to administer sedation as needed. Participants will stay in the Pediatric Intensive Care Unit (PICU) overnight. Prophylactic enteral prednisone or prednisolone and sirolimus will be administered to all participants. Certain participants will require additional immunosuppression with tacrolimus as defined in the immune modulation protocol (protocol section 8.6).

6.2.1: Dose Selection Rationale

6.2.1.1: Justification of clinical study dose

Table 2: Relationship Between Preclinical Study Doses and Proposed Human Intrathecal Dose

Species	Injection Volume (mL)	Weight (kg)	CSF Volume (mL)	Dose as % of CSF Volume	Max Total Human Dose (vg) ***	Min Dose per CSF Volume (vg/mL)	Max Total Mouse Dose (vg)	Max Dose per CSF Volume (vg/mL)
**Pre-clinical:**								
Mouse	0.005	~0.025	0.035	14	2.5×10^{11}	7×10^{12}	8×10^{11}	2.2×10^{13}
Rat	0.02	0.25	0.25	8.0	1.8×10^{12}	7×10^{12}	5.8×10^{12}	2.2×10^{13}
NHP*	1.0	3-6	12	8.3	8.4×10^{13}	7×10^{12}	2.7×10^{13}	2.2×10^{13}
**Clinical:**								
Human (≥4 years)**	10	~40	140	7.1	1×10^{15}	7×10^{12}	3.2×10^{15}	2.2×10^{13}

For CSF Volumes, we used the following references:[9, 10, 11]
* Provided for reference. Additional NHP studies are not proposed.
** For human participants <4 years old, a dosing strategy is outlined in Table 3.
***This is the proposed high dose in humans and extrapolated dose from preclinical models. The low dose is half of the high dose.

6.2.1.2: Rationale for brain size dependent dose calculation

Table 3: Dose Extrapolation Based on Age and Brain Size

Age (years)	Brain Volume (approx. cm^3)	Infusion volume (mL)	Total IT High Dose ($\times 10^{14}$ vg)
4+	1312	10	10
3	1180	9	9
2	1080	8.2	8.2
1	955	7.3	7.3
0.5	525	4	4
Newborn	400	3	3

Normative average brain volumes across ages are used to calculate doses [12, 13, 14]. Calculations for the High Dose cohort are shown. The concentration of the high dose injection solution is kept constant at 1×10^{14} vg/mL, and lower volumes are administered to younger patients to maintain approximately the same vg dose per brain volume using the calculation: ([average brain volume]/1312 cm^3) x 10 mL = infusion volume. For the lower dose (1/2 the high dose), all calculations are the same except the injection solution concentration is kept constant at 5×10^{13} vg/mL.

6.2.2: Anesthesia Safety

Participants will undergo a comprehensive pre-anesthesia evaluation during the screening period. Physiologic monitoring in accordance with the standards set by the American Society of Anesthesiologists will be utilized for all participants while they are receiving analgesia/anesthesia and until they have fully recovered from its effects.

Active warming devices will be used during anesthesia as needed since patients are prone to hypothermia during the anesthesia.

6.2.3: Post-Procedure Recovery

After the procedure, participants will be transported to a Post Anesthesia Care Unit (PACU) or PICU with continuous pulse oximetry monitoring and oxygen if needed by bag/mask/nasal canula or blow-by. Vital signs including heart rate, respiratory rate, blood pressure, and pulse oximetry will be monitored every 15 minutes for the first 2 hours post infusion, every 30 minutes during the third and fourth hour post infusion, then hourly for 4 hours, and finally every 4 hours until discharge. In the event that abnormalities are detected, appropriate medical intervention will occur, including the possibility of extending the hospitalization and/or subsequent testing. If low oxygen saturation is measured, the patient will be evaluated and treated as clinically indicated. This may include, but are not limited to, assessments on physical examination, initiating of oxygen therapy, measurement of blood oxygen levels by ABG (arterial blood gas), Chest X-ray, CT imaging of the chest, PFTs (pulmonary function tests), or aerosol therapy. In the unlikely event that a severe allergic reaction should occur, the medical and nursing staff will follow the anaphylaxis guidelines.

6.3: Dosing Device for Phase I Clinical Trials

An atraumatic Sprotte needle will be inserted percutaneously at the lumbar level into the intrathecal space of the spinal column. Spinal needle placement will be confirmed using fluoroscopic intraoperative imaging (C-Arm) scanner at the chosen injection site prior to and after vector administration.
A volume of CSF approximately equal to the infusion volume will be withdrawn from the lumbar thecal sac. The AAV9/CLN7 vector solution will be loaded into a 20 mL BD syringe, connected to the needle with 60 inch mini volume IV extension tubing and a Braun 4-way stopcock. The vector solution is then infused at a rate of 1 mL per minute, using a CareFusion Alaris 8110 syringe pump.

6.4: Endpoints

6.4.1: Safety

The primary endpoint of this study is to evaluate the safety and tolerability of single doses of AAV9/CLN7 administered intrathecally to children with CLN7 disease by the incidence and severity of treatment related serious adverse events (SAEs).

A DSMB is appointed and will review all relevant safety data from the first participant at Day 30 before a second participant is dosed. Similarly, the third participant will not be dosed until safety on the second patient has been reviewed by the DSMB at 30 days post-injection.

Thirty days following the dosing of the third patient, data will be reviewed by the DSMB to determine if any further participants will be enrolled utilizing the same dose or a lower dose according to the conditional dose de-escalation plan.
Monitoring and follow-up for safety and efficacy will be done in two parts, referred to as Period A and Period B in this document. Period A will include safety and short-term efficacy assessments. In Period A (early monitoring up to 24 months), participants will be tested at screening/baseline (-28 to -7 days), participants will be closely monitored for 24 hours in PICU followed by another 24 hours in step-down in patient unit after AAV9/CLN7 dosing. They will return for outpatient follow-up visits on Days 7, 30, 60, 90, 180, 270, 360, 540, and 720. In addition, there will be lab visits every week during the first month and every other week during the second and third month after dosing (see Table 8 for complete visit schedule). Participants will also undergo MRI brain as safety assessments on Days 90, 180, 360, 540 and 720. Participants will also undergo a LP with CSF analysis as safety assessments on Days 90, 180, 360, and 720. In Period B, participants will be assessed at annual visits for five (5) years after completion of Period A for long-term safety, as per FDA guidance for gene therapy studies, and efficacy

assessments. During this period in between annual visits, participants/parents/guardians will be encouraged to contact the study team for any suspected adverse event reporting. Unscheduled visits may occur if the PI determines they are necessary to assess safety, repeat labs, etc.

Reports outlining all adverse events and serious adverse events will be provided to the DSMB throughout the course of the study.

6.4.2: Efficacy

The secondary endpoint of this study is to explore the efficacy of single dose AAV9/CLN7 administered intrathecally to children with CLN7 disease. Clinical assessments to assess disease burden will be used including Clinical Global Impression, neuropsychological assessments, and physiotherapy evaluations. These assessments will be completed every 6 months.

6.4.2.1: Disease Burden Assessments

6.4.2.1.1: Clinical Global Impression (CGI)

The CGI is a 3-item observer-rated scale that measures illness severity (CGI-S), global improvement or change (CGI-C), and therapeutic response.

The CGI is rated on a 7-point scale, with the severity of illness scale using a range of responses from 1 (normal) through to 7 (amongst the most severely ill patients). The Early Clinical Drug Evaluation Program (ECDEU) version of the CGI asks that the clinician rate the patient relative to their past experience with other patients with the same diagnosis. Treatment response ratings should take account of both therapeutic efficacy and treatment-related adverse events and range from 0 (marked improvement and no side effects) to 4 (unchanged or worse and side effects outweigh the therapeutic effects). Each component of the CGI is rated separately and the instrument does not yield a global score.

6.4.2.2: Neuropsychology Assessments

Table 4: Neuropsychology Assessments

**Assessment**	**Description**
Mullen Scales of Early Learning	A comprehensive assessment of early language, motor, and cognitive abilities
Vineland Adaptive Behavior Scales, 3rd Edition	A standardized questionnaire administered in a structured interview format that assesses key domains of adaptive functioning
Quality of Life Assessments	Standardized questionnaires of quality of life including social interactions, positive emotions, physical health, negative emotions, leisure skills, and independence that parents complete via paper and pen.

6.4.2.2.1: Mullen Scales of Early Learning (Mullen)

The Mullen is a test of early cognitive ability and motor development for use with children from birth to 68 months. The Mullen includes assessments of gross motor coordination, fine motor coordination, visual reception (visual problem-solving), and receptive and expressive language abilities. The Mullen domain scores are T-scores. Age

equivalents are also obtained for all domains. There is also a composite score of Early Learning that is a standard score.

Administration and scoring guidelines are included in the manual. Split-half reliability and test-retest reliability range from good to excellent and are found in the technical manual.

The Mullen is administered by a trained neuropsychologist. Even though the Mullen is normed for children through 68 months, it will be administered to children older than 68 months and an age-equivalent will be generated.

6.4.2.2.2: Vineland Adaptive Behavior Scales, 3rd Edition (Vineland-3)

Adaptive behavior can be defined as the performance of daily activities required for personal and social sufficiency. Adaptive behavior is impacted in virtually all disorders that cause cognitive/developmental regression. The Vineland-3 is one of the most well-known and well-validated measures of adaptive functioning. The age span is birth to age 90. This test will be administered in a structured interview format with the participants/parents/guardians, which reduces the impact of parental reporting bias on scores. Four key domains will be rated, including communication skills, daily living skills, social skills, and motor skills. The combination of these makes up the Adaptive Behavior Composite. Scores are based on standard scores for the domains and age-equivalents and v-scores for sub-domains.

Administration and scoring guidelines are included in the manual. From the Vineland-3 Technical Manual, the average internal consistency reliability coefficients for the Vineland-3 composites range from .90 to .98 (excellent range). The majority of the test-retest reliability coefficients are also in the excellent range, and inter-rater reliability coefficients range from good to excellent.

6.4.2.3: Quality of Life Assessments

Table 5: Quality of Life Assessments

Assessment	Description
Quality of Life Inventory-Disability (QI- Disability)	A standardized questionnaire of quality of life including social interactions, positive emotions, physical health, negative emotions, leisure skills, and independence that parents complete via paper and pen.
Infant/Toddler Quality of Life Questionnaire (ITQOL)	A standardized questionnaire of quality of life measure looking at physical and psychosocial domains that parents complete via paper and pen.

6.4.2.3.1: Quality of Life Inventory – Disability (QI-Disability)

The QI-Disability is a way to capture the health and well-being of children ages 5-18 with intellectual disability [15, 16]. This measure was particularly developed to assess children with a wide range of disabilities and has been validated on children with Rett Syndrome, Down Syndrome, Cerebral Palsy, Autism Spectrum Disorder, and CDKL5 deficiency disorder [17]. Both Rett Syndrome and CDKL5 deficiency disorder are most similar to our study population. The validation on children with CDKL5 deficiency disorder spanned children as young as 3 years of age. This 32-item measure assesses a child’s social interactions, positive emotions, physical health, negative emotions, leisure skills, and independence. Scores are a 5-point Likert scale and then are transformed to a scale of 0 to 100 with higher scores indicating better quality of life.

Administration is conducted by handing out the questionnaire to parents either in paper or online, or via telephone interview by qualified study personnel. Scoring is completed via a rubric provided by the test developer. Close communication via the study team and test developer is ongoing and timely regarding questions and expansion of administration to younger populations. Test-retest reliability across a 35-day span was excellent for the total score and four domains (adjusted ICC ≥ 0.80), good for physical health (adjusted ICC = 0.68) and fair for positive emotions (adjusted ICC = 0.58) (Jacoby, et al., in press).

6.4.2.3.2: Infant and Toddler Quality of Life Questionnaire – Short Form (ITQOL)

The ITQOL Short Form is a 47-item measure to assess physical and psychosocial functioning of children ages 2 months to 5 years. It contains 12 scales including physical functioning, growth and development, bodily pain, temperament and moods, general behavior, getting along, general health perceptions, parental impact-emotional, parental impact-time, family activities, family cohesion, and change in health. Scores are transformed to a scale of 0 to 100 with higher scores indicating better quality of life.

Administration and scoring guidelines are included in the manual. Test-retest reliability across a 2-week span was good for 4 scales (ICC ≥ 0.70; general behavior, getting along, general health, family cohesion), moderate for 6 scales (ICC 0.50–0.70; physical functioning, growth and development, temperament and mood, parental impact-emotional, parental impact-time, family activities); poor for bodily pain (ICC below 50).

These measures were chosen as the questions on the QI-Disability are thought to be more appropriate for our patients with more significant neurological impairment. Given that the validation has been primarily on children ages 5-19 with only a single study on ages 3-5, a second QOL measure to examine children under age 5 was selected. The ITQOL was selected over the PedsQL Infant Scale as the ITQOL has been validated for children 2 months to 5 years. PedsQL Infant Scale only spans children 1-24 months. For our sample, parents of children ages 1-5 will complete both QOL measures so that comparative data can be collected and analyzed in order to identify which measure is most valid for our population. Parents of children ages 5 and above will complete only the QI-Disability [18].

6.4.2.4: Ataxia and Motor Function Assessments

Table 6: Ataxia and Motor Function Assessments

Assessment	Purpose
2-Minute Walk Test OR<br>6-Minute Walk Test	Measures endurance in ambulatory individuals over age 3
Pediatric Balance Scale	Measures balance (static and dynamic) in children over age 2
Gross Motor Function Measure	Measures self-initiated movement in both ambulatory and non-ambulatory individuals

6.4.2.4.1: Timed Walk (Two Minute Walk Test or Six Minute Walk Test)

The timed walk test will be completed by participants who can ambulate sufficiently. The Two Minute Walk Test (2MWT) measures the distance that an individual over age 3 can walk on a flat surface in a period of 2 minutes, and assesses endurance relative to the musculoskeletal, pulmonary, and cardiovascular systems.

The Six Minute Walk Test (6MWT) measures endurance in a similar fashion using a time interval of 6 minutes and is a commonly used measure for evaluating outcomes in research for multiple different populations of children and adults.

6.4.2.4.2: Pediatric Balance Scale

The Pediatric Balance Scale is a 14-item test used to evaluate functional balance in children. It is an adapted version of the Berg Balance Scale. It contains both static and dynamic components and has normative values available for children ages 2 to 17. This test will allow for assessment of balance in children who have vision and for those who have visual impairments. It will also allow for monitoring of changes in balance over time. A maximum score of 56 points is possible with scores for each item from 0-4.

6.4.2.4.3: Gross Motor Function Measure (GMFM)

This is a criterion-referenced measure consisting of 5 Domains (Lying & Rolling, Crawling & Kneeling, Sitting, Standing and Walking, Running, and Jumping). This test has been used in children with Cerebral Palsy as an outcome measure, but has also been used to document outcomes in other populations. It is based on a child's performance of self-initiated functional movements including typical developmental motor milestones. This test will allow for monitoring of gross motor abilities and changes over time. The Lying & Rolling, Crawling & Kneeling, and Sitting Domains will be important for evaluating children who are non-ambulatory.

The proposed outcome measures for this study are included for the purpose of tracking change in motor function over time in participants. The 6MWT [19] or 2MWT [20, 21] will allow for the measurement of musculoskeletal and cardiopulmonary endurance. The 6MWT has been used in studies of children with Duchenne Muscular Dystrophy (DMD) and demonstrated excellent test-retest reliability with an ICC of 0.92 [22]. The minimal clinically important difference (MCID) measured 28.5 and 31.7 meters as determined by two statistical distributions. Additionally, the 6MWT has been used in one study of children with CLN3 disease [23]. The study participants showed deterioration in 6MWT performance over time that corresponded to progression of the disease. The 2MWT is a modified version of the 6MWT and will be used with children who do not have sufficient endurance to complete the 6MWT [20,21,24]. The 2MWT has been compared to the 2MWT in children with DMD, and both tests were found to have good test-retest reliability. The 2MWT was also utilized by Stahlhut, Downs, Leonard, Bisgaard, and Nordmark to evaluate walking in girls with Rett Syndrome [25]. The test-retest reliability was found to range from 0.86-0.98, and it demonstrated a standard error of measurement (SEM) of 13 meters. Rett Syndrome generally results in deterioration of motor function over time, however, the researchers in the Stahlhut et al. study were able to conduct this test in a modified fashion with good test-retest reliability.

Additionally, the Pediatric Balance Scale [26] is a modified version of the Berg Balance Scale that has been used in previous research studies with children. The Pediatric Balance Scale has normative values for children ages 2 to 13 years and will allow investigators to detect change over time in this study population. Franjoine and Darr found the test-retest reliability to be .998 and inter-rater reliability to be excellent (ICC = 0.997) [27]. Further, Chen, Shen, Chen Wu, Liu, et al. tested children with Cerebral Palsy and found a minimal detectable change (MDC) of 1.59 (Total Score). The minimal clinically important difference (MCID) measured 3.66-5.83. The Pediatric Balance Scale consists of both static and dynamic items, and Chen et al. evaluated the MDC and the MCID of both of these components. [28] Due to the potential of study participants having visual impairment, having subscales of static and dynamic components will allow for identification of changes in these areas separately. Two studies have used the Pediatric Balance Scale with children having visual impairment [29, 30]. The fact that the researchers were able to perform this assessment with children who have limited vision suggests its usefulness for this study.

The Gross Motor Function Measure-88 (GMFM-88) is an assessment that has been used extensively in research with children who have Cerebral Palsy [31]; however, it will be an important measure in this study to detect small changes in function in children who are non-ambulatory. Ko and Kim found the overall reliability of the GMFM to be excellent (ICC= 0.952-1.00). Inter-rater reliability was >0.97 and intra-rater reliability was >0.94 in their study evaluating responsiveness and reliability of the GMFM -88 conducted with children having Cerebral Palsy [32]. The GMFM has been shown to be valid and reliable in studies conducted with children who have Hereditary Spastic Paraplegia (HSP) and children with Spinal Muscular Atrophy (SMA) [33]. Both of these conditions are progressive in nature and typically result in a decline in motor function over time. Due to the similar neurological deterioration

that characterizes CLN7, the Gross Motor Function Measure therefore has potential as a meaningful tool to track change in individuals with this disease.

6.4.2.5: Surrogate Assessments

6.4.2.5.1: Electroencephalogram (EEG)

Participants will undergo a scalp EEG at baseline and post-injection Days 180, 360, 540, and 720. Scalp EEG is an important test that records the electrical activity of the brain. This test is safe and painless. Electrodes are attached to the participant's scalp which are connected to an electric box. These electrodes do not transmit any electricity to the scalp. The electric box is connected to an EEG machine which records the tracing of electric activity. Video is recorded during this EEG. Video EEG will be completed for an hour in this study. Different stimuli like photic stimulation (flashing lights) and hyperventilation (if possible) are performed during EEG. EEG is a safe test; however photic stimulation may provoke seizures.

6.4.2.5.2: Magnetic Resonance Imaging of Brain (MRI)

Brain MRI (with or without contrast) will be done at baseline and at 6 month intervals. MRI studies will be done at Children's Health on the same machine each time. The pulse sequences include localizer images and the following:

- MPRAGE (3D sagittal T1 with TR/TE/TI=2500/3.11/1240 ms; flip angle =8 degree, slice thickness=1 mm, in plane acquisition resolution 0.8mm and reconstructed into 0.4 mm pixel size, acquisition time 5:14 min:sec)
- Diffusion tensor and kurtosis imaging (b=1000, 2000 s/mm^2; voxel size 3x2.7x2.7mm^3, 30 directions for each shell, acquisition time 12:14 min:sec)
- 3D T2 FLAIR (TR/TE/TI=5000/394/1800 ms, slice thickness=1mm, in plane acquisition resolution 1 mm and reconstructed into 0.5 mm pixel size, acquisition time 6:07 min:sec)
- Single voxel proton spectroscopy (TR/TE=2000/30ms, voxel size 30x30x30mm^3, the ROI is located at the frontal lobe centerline across the right and left hemispheres, 80 averages and acquisition time 2:50 min:sec).
- When clinically indicated, or radiographic changes are observed, T1 post contrast sequences will be obtained

6.4.2.6: Exploratory Assessments

6.4.2.6.1: Ophthalmologic Evaluation

Participants will undergo an ophthalmologic exam to assess disease progression involving the eye. Testing may include electroretinography (ERG) and dilated eye exam. Formal visual acuity and OCT will be completed only in participants who are able to cooperate with the testing. Ophthalmologic evaluations will be completed at the Screening/Baseline visit and on Days 360 and 720.

6.4.2.6.2: Swallow Function Test

A swallow study will be performed to determine whether the patient has signs of aspiration. The swallow function test will be completed at the Screening/Baseline visit and on Days 360 and 720.

6.4.2.7: Diaries

6.4.2.7.1: Seizure Diary

Parent(s)/guardian(s) will be provided with a diary for recording frequency and duration of their child's seizures. Parents/guardian(s) will record seizure activity and the seizure diary will be completed at baseline and on Days 90, 180, 270, 360, 540, and 720.

6.4.2.7.2: Healthcare Utilization Diary

Parent(s)/guardian(s) will be provided with a diary for recording frequency and type of healthcare utilization required for care of their child. This will be completed at baseline and on Days 90, 180, 270, 360, 540, and 720.

6.4.2.7.3: Telephone Adverse Event Monitoring

Long term adverse event monitoring will include annual investigator/research team member telephone contact with subject's parent(s)/guardian(s) on an annual basis in Part B.

6.5: Duration of Study

Participants will be enrolled in the study for a duration of 24 months for part A. Part B consists of an additional 5 years of follow-up.

6.6: External Data Monitoring

We have appointed a DSMB who will review all labs and safety data from the first patient at Day 30, before initiating dosing of a second participant. Similarly, the third participant will not be dosed until safety data from the second participant has been reviewed by the safety officer at 30 days post-injection.

Thirty days following the dosing of the third participant, data will be reviewed by the DSMB to determine if further participants will be enrolled utilizing the same dose, or a lower dose, according to the conditional dose de-escalation plan.

See Section 10 for complete data and safety monitoring plan.

7.0 Selection of Participants

7.1: Inclusion Criteria

- 1-18 years of age
- Clinically symptomatic patients with diagnosis of CLN7 based upon molecular testing with homozygous or compound heterozygous, pathogenic or likely pathogenic mutations in CLN7 gene with symptom onset before age 4
- Clinically pre-symptomatic patients with molecularly confirmed diagnosis of CLN7 with homozygous or compound heterozygous pathogenic mutations in CLN7 gene less than or equal to 4 year of age
- Written informed consent provided by participant/parent/guardian and willingness to participate and comply with all the study related visits and procedures. Assent provided by children 10 -17 years old based on their ability to understand the risks and possible benefits, and the activities expected of them as participants.

7.2: Exclusion Criteria

- Diagnosis of a second neurodegenerative disease or another genetic syndrome with a progressive course.
- Hypersensitivity to any drugs used for study procedure
- Inability to tolerate anesthesia or study procedures.
- Advanced stage disease defined by the use of chronic invasive ventilatory support (tracheostomy with ventilator dependence) and a non communicative status
- Concomitant illness that places patient at risk for gene transfer or gene transfer related procedures and immunosuppression

- Active viral infection (including HIV or serology positive for Hepatitis B or C, or COVID-19).
- Family is unwilling to or unable to participate with required follow-up assessments
- Abnormal lab values that are clinically significant:
  - Platelet count < 100,000/mm^3
  - Abnormal absolute neutrophil count (ANC) of < 1000/mm^3
  - Persistent leukopenia or leukocytosis (total white blood cell count < 3,000/mm or > 15,000/mm respectively)
  - Significant anemia (Hb <10 g/dL)
  - Abnormal prothrombin (PT) or partial thromboplastin time (PTT)
  - Abnormal liver function tests (>2 X ULN or > 2 X the baseline value)
  - Abnormal pancreatic enzymes (>2 X ULN or > 2 X the baseline value)
  - Patients with renal impairment defined as urinary protein concentration greater than or equal to 0.2 g/L on two consecutive tests
  - Any other abnormal lab values that are clinically significant, per PI's discretion
  - Note – If labs are abnormal, these can be rechecked during the screening period. If labs normalize with or without intervention, the patient can be enrolled at the discretion of PI.
- Contraindications for intrathecal administration of the product via lumbar puncture, such as bleeding disorders or other medical conditions (e.g., spina bifida, clotting abnormalities)
- Contraindications for MRI scans (e.g., cardiac pacemaker, metal in the eye, aneurysm clip in the brain)
- History of or current chemotherapy, radiotherapy, or other immunosuppression therapy within 30 days preceding screening (corticosteroid treatment may be permitted at the discretion of the PI)
- Receipt of any other investigational product within the past 3 months
- Positive beta hCG pregnancy test (females of child bearing age will have pregnancy test at Day-1)
- Any other medical condition that puts the subject at risk of adverse events related to the study drug or study related procedures

7.3: Duration of Inclusion of Participants

Each participant will be enrolled in the study for 84 months.

7.4: Participant Withdrawal Criteria

Participants/parents/guardians will be consented prior to their enrollment in the study. They will be made aware that participation in the study is voluntary and they can withdraw at any time. Participants can be withdrawn from the study if they:

- Withdraw consent
- Are unable to tolerate the dose of the drug
- Are unable to participate in the follow-up assessments

8.0 Treatment

8.1: Investigational Product

The study agent, AAV9/CLN7 vials, will be formulated as a concentrated stock in phosphate-buffered saline (PBS) containing 5% D-sorbitol and 0.001% pluronic F68, and stored at ≤ -80˚C until the day of the administration procedure(s). The solution will be thawed within 4 hours of administration and diluted to the appropriate final dosage concentration and volume using PBS with 5% D-sorbitol and 0.001% pluronic F68 (if necessary).

8.2: Packaging

AAV9/CLN7 and diluent are provided in 2 mL Corning screw-cap polypropylene cryovials (1.05 mL per vial).

8.3: Labeling and Storage

AAV9/CLN7 and diluent are labeled with the lot/batch number, individual vial number, contents, and manufacture date, along with a warning that they are for investigational use only. They will be stored at or below -80˚C in room Y4.204 on the campus of UTSW. This is a locked and monitored freezer located within the UTSW Viral Vector Facility where drug product will be stored until transferred to the Children’s Health Investigational Drug Services (IDS) Pharmacy for preparation.

8.4: Dose Administration

CLN7 participants will have a spinal needle inserted percutaneously at the lumbar level into the intrathecal space of the spinal column. A volume of CSF approximately equal to the infusion volume is withdrawn from the lumbar thecal sac. The vector solution is then infused at a rate of 1 mL per minute. The participant will remain at 15-degree Trendelenburg (head down) position for 1 hour following vector administration.

The first participant will receive a low dose of $5x10^{14}$ vg. If this is tolerated well then, there are no safety concerns and approval is given by the DSMB, we will proceed to dose participants 2-4 at $1x10^{15}$ vg. A $1X10^{15}$ dose of the AAV9-CLN7 (or $5x10^{14}$ vg for the low dose) will be administered to participants older than 4 years of age. For participants younger than 4 years, the dose is calculated based on brain volume (Table 3).

8.5: Dose De-Escalation Plan

Upon DSMB review of the initial safety data, doses may be decreased to $5x10^{14}$ vg if there is a concern for participant safety based on the higher dose of the investigational agent. A dose comparable to this (scaled to mice by CSF volume) was able to confer a benefit to CLN7 mice, albeit a lower benefit than the higher dose.

8.6: Immune Modulation Protocol

Since an antigen specific T-cell response to the AAV9 vector is anticipated during the 2-12 weeks post-transfer similar to other related gene therapy studies [34, 35], there is a potential for clearance of the transduced cells and loss of transgene expression due to antigen specific T-cell responses. Thus, to reduce the risk of the host immune response to our AAV9-based therapy, participants will be started on prophylactic enteral prednisone or prednisolone (approximately 1 mg/kg/day, maximum dose 60 mg). On the day of the intrathecal (IT) procedure marked as Day 0, participants will receive a bolus dose of IV methylprednisone at 10mg/kg (maximum dose= 500mg IV) prior to the IT gene transfer. Maintenance doses of enteral prednisone/prednisolone will be started the day after the gene transfer. This will be continued after gene transfer for 3 months (plus a taper period) to prevent immune responses to the vector associated with the AAV9/CLN7 administration. This protocol has been used in several gene transfer protocols (NCT02716246, NCT03315182, NCT02362438, and NCT02122952). Additionally, participants will receive the immune suppressant sirolimus to further reduce the risk of deleterious anti-AAV9 and/or anti-CLN7 T-cell responses starting during screening.

After 3 months of maintenance steroids, at the Day 90 visit, safety labs, CSF analysis and an ELISpot assay will be completed. If normal, the prednisone/prednisolone dose will be reduced by 15% increments per week. This taper will start after lab results are obtained. The taper will be over 4-6 weeks and the dose will be decreased per the steroid tapering schedule provided under section 9.4 (post-dose monitoring). The taper will continue until the participant reaches a dose of 5 mg. After participants are on 5mg for 1 week, an 8am cortisol level will be checked. If the 8 AM cortisol level is normal, prednisone/prednisolone will be tapered off in 1 mg decrements every 4-7 days. If the last dose is above 5 mg, it will be rounded down to 5 mg between Day 120-134. All participants will be off steroids by Day 150. At Day 180, if they tapering guidelines have been met, scheduled safety and immunomodulation labs and CSF analysis will be checked.

If safety lab results are not normal at any point during the time participants are on maintenance steroids, the prednisone/prednisolone dose may be increased to 2 mg/kg/day (max daily dose 60mg) at the PI's discretion, depending on a T-cell response against CLN7 protein measured by ELISpot assay specifically. The PI might also decide to prolong the tapering protocol based on the individual participant's immune response to the gene transfer, again assessed by ELISpot assay specifically for CLN7 protein, or based on other monitoring tests. Should the participate experience an acute neuroinflamatory response, the management may include pulsed high dose IV methylprednisolone per PI discretion.

Of note, all the participants will also be on sirolimus. One of the potential adverse effects of sirolimus is transaminitis. If AST and/or ALT is elevated, the sirolimus dose can be held for a week to determine if this resolves the abnormalities in AST/ALT, per PI's discretion.

Strategies for long-term monitoring of immune responses will differ based on the participant's CRIM (cross-reactive immune material) status relative to endogenous production of the CLN7 protein at baseline. If the participant is predicted to produce some CLN7 protein at baseline (CRIM-pos), then they will follow the standard immunomodulatory regimen which will consist of prednisone/prednisolone and sirolimus. If the participant is predicted not to produce any CLN7 protein at baseline (CRIM-neg), then they will follow the alternate immunomodulatory regimen which will consist of prednisone/prednisolone, sirolimus, and tacrolimus. These participants will be put on sirolimus indefinitely based on their measure of tolerance and at the PI discretion.

In addition, as participants are immunocompromised, participants will be placed on Bactrim (trimethoprim/sulfamethoxazole) for prophylaxis against opportunistic infections such as documented Pneumocystis jirovecii pneumonia (PCP). Dosing will be 150 mg TMP/m²/day enteral divided q12 hr for 3 days/week on consecutive or alternate days. If a participant has an allergy to trimethoprim/sulfamethoxazole, Dapsone will be used at 4 mg/kg/dose enteral qWeek (not to exceed 200 mg/week).

8.6.1: For participants with CRIM-pos status

Enteral prednisone or prednisolone along with sirolimus administration will be continued following discharge, and will then be tapered according to liver function testing, CSF anaylsis and IFN-gamma producing T-cell studies in the following weeks. If post-infusion IFN-gamma T-cell responses remain ≤ 50 SFC per $1x10^6$ PBMCs, the subject's prednisone/prednisolone dose will begin to be tapered around Day 90 after gene transfer. Tapering will proceed slowly, typically over 4 to 7 weeks. For tapering, if either the AST and ALT exceeds > 2.5 times the upper normal limit values and results are confirmed on a follow-up blood test within three days, the prednisone/prednisolone regimen will be maintained at 1 mg/kg/day until the enzyme levels fall below these levels. As discussed above, sirolimus can cause elevation in AST/ALT, and if the abnormalities in these labs are considered secondary to sirolimus side effects, then sirolimus could be held for a week at the PI's discretion. Similarly, sirolimus will be tapered starting at 10 months post-injection (see immune modulation protocol above).

8.6.2: For participants with CRIM-neg status

Enteral prednisone or prednisolone administration will be continued following discharge and will then be tapered according to liver function testing and IFN-gamma T-cell studies in the following weeks. If post-infusion IFN-gamma T-cell responses remain ≤ 50 SFC per $1x10^6$ PBMCs, the participant's prednisone/prednisolone dose will begin to be tapered around Day 90 after gene transfer. Tapering will proceed slowly, typically over 4 to 7 weeks. For tapering, if either the AST and ALT exceeds > 2.5X the upper normal limit and results are confirmed on a follow-up blood test within three days, the prednisone/prednisolone regimen will be maintained at 1 mg/kg/day until the enzyme levels fall below this amount. Similarly, tacrolimus and sirolimus will be continued following discharge. Tacrolimus will be tapered starting at 6 months post-injection over the course of 6-8 weeks. Participants will remain on sirolimus indefinitely.

8.6.3: For Mutations with Predicted Residual Protein Function

One Week Prior to Vector Administration:

- Sirolimus load: 1 mg/m2 every 4 hours x 3 enteral doses (load only given on one day)
- Starting the day after the sirolimus load, begin enteral daily dosing at 0.5 mg/m2/day, divided in twice per day dosing (goal level: 4-8 ng/mL)

Day of Vector Administration (Day 0):

- Acetaminophen (15 mg/kg/dose enteral; maximum 650 mg per dose)
- Diphenhydramine (0.5 mg/kg/dose enteral; maximum 50 mg/dose)
- IV methylprednisolone (10 mg/kg to a maximum single dose of 500 milligrams, infused over 30 minutes)

Day after Vector Administration (Day 1):

- Begin daily enteral prednisone/prednisolone at 1 mg/kg/day x 3 months.
- Continue enteral daily sirolimus dosing at 0.5mg/m^2/day, divided in twice per day dosing (goal level: 4-8 ng/mL)

Maintenance:

- Enteral Prednisone/Prednisolone 1 mg/kg/day x 3 months, then taper according to schedule
- Sirolimus 0.5 mg/m^2/day, divided in twice per day dosing until tapering at approximately 10 months. Tapering at 10 months (following vector administration) will be completed by 12 month post-gene-transfer. If there are signs or symptoms of transgene mediated CNS inflammation by examination, brain imaging, and/or laboratory testing, longer administration of immunomodulatory medications and possibly addition of other immunomodulatory agents (such as tacrolimus) may be required.

Monitoring:

- Weekly BP checks x 4 weeks
- Sirolimus troughs every 1 week x 4, then every 2 weeks x 4, then monthly once levels are stable within the desired range. CBC with differential testing at every blood draw.
- 8 AM cortisol level after participants have been on a 5 mg dose prednisone/prednisolone for 1 week
- Monthly fasting lipid profile while on immunomodulation and at PIs discretion

8.6.4: For Null Mutations

1 Week Prior to Vector Administration:

- Sirolimus load: 1 mg/m^2 every 4 hours x 3 doses (load only given on one day)
- Starting the day after the sirolimus load, begin enteral daily dosing at 0.5 mg/m^2/day, divided in twice per day dosing (goal level: 4-8 ng/mL)

Day of Vector Administration (Day 0):

- Acetaminophen (15 mg/kg/dose enteral; maximum 650 mg per dose)
- Diphenhydramine (0.5 mg/kg/dose enteral; maximum 50 mg/dose)
- IV methylprednisolone (10 mg/kg to a maximum single dose of 500 milligrams, infused over 30 minutes)

Day after Vector Administration (Day 1):

- Begin daily enteral prednisone/prednisolone at 1 mg/kg/day x 3 months.
- Continue enteral daily sirolimus dosing at 0.5mg/m^2/day, divided in twice per day dosing (goal level: 4-8 ng/mL)
- Tacrolimus at 0.1 mg/kg/day divided into twice daily dosing (goal level: 4-8 ng/mL)

Maintenance:
- Enteral prednisone/prednisolone at 1 mg/kg/day x 3 months, then taper according to schedule
- Sirolimus 0.5 mg/m^2/day, divided in twice per day dosing. If there are signs or symptoms of transgene mediated CNS inflammation by examination, brain imaging, and/or laboratory testing, longer administration of immunomodulatory medications and possibly addition of other immunomodulatory agents may be required.
- Tacrolimus at 0.1 mg/kg/day divided into twice daily dosing (goal level: 4-8 ng/mL); tacrolimus will be continued for 6 months and will begin taper by 7 months after gene transfer. The taper will be started if there are no signs or symptoms of transgene mediated CNS inflammation by examination, brain imaging, and/or laboratory testing, which if present may require longer administration of immunomodulatory medications.

Monitoring:
- Weekly BP checks x 4 weeks.
- Sirolimus troughs every 1 week x 4, then every 2 weeks x 4, then monthly once levels are stable within the desired range.
- Tacrolimus troughs every 1 week x 4, then every 2 weeks x 4, then monthly once levels are stable within the desired range
- CBC with differential testing at every blood draw
- 8 AM cortisol level when participants are on 5 mg dose prednisone/prednisolone for 1 week
- Monthly fasting lipid profile while on immunomodulation and at PIs discretion

9.0 <u>Study Assessments</u>

<u>9.1: Screening/Baseline (Days -28 to -7)</u>

The following evaluations will be conducted during their Screening/Baseline Visit prior to gene transfer:
- Medical history
- A complete physical examination will include but may not be limited to the following systems: general, head and neck, eyes, ears and throat, chest, lungs, heart/pulse, abdomen, and skin
- Neurologic exam will include but may not be limited to the testing of cranial nerves, muscle bulk/tone and strength, sensation, cerebellar function, involuntary movements, myotatic reflexes, toe sign, gait, and stance
- Concomitant medication history
- Vital signs (temperature, heart rate, respiratory rate, blood pressure, oxygen saturation)
- Height, weight and head circumference
- Electrocardiogram
- Echocardiogra.
- 1-hour video EEG
- Brain MRI (3T) with MR Spectroscopy and Diffusion Tensor Imaging
- Safety-related laboratory studies:
  - Complete blood cell count (CBC) and differential with smear
  - Electrolytes
  - Blood urea nitrogen (BUN)
  - Total calcium
  - Creatinine
  - Aspartate aminotransferase (AST)
  - Alanine transaminase (ALT)
  - Gamma-glutamyl transpeptidase (GGT)
  - Alkaline phosphatase
  - Serum total bilirubin

  - Serum total protein
  - Albumin
  - Prothrombin time (PT) and INR
  - Activated partial thromboplastin time (PTT)
  - Serum glucose
  - Amylase
  - Lipase
  - Urinalysis
  - Capillary blood gas
- Blood for DNA isolation
- Alpha-fetoprotein
- Hepatitis (A, B, C), HIV and COVID-19
- Plasma biobanking
- Blood DNA and RNA biobanking
- Biological fluid samples for vector shedding (urine, saliva, feces, and plasma)
- ELISpots for T-cell responses to AAV9 and CLN7 protein
- ELISA for detection of total antibodies to AAV9
- Confirm diagnosis of CLN7 via gene sequencing
- Ophthalmologic assessments will include electroretinogram (ERG) and dilated eye exam. Visual acuity and ocular coherence tomography (OCT) will be completed if participants are clinically able.
- Swallow study
- Global disease burden assessments:
  - Clinical Global Impression (CGI)
  - Neuropsychological assessments done by neuropsychologist (see Table 4)
  - Participant/parent/guardian reported quality of life measures (see Table 5)
  - Motor assessments done by physical therapist (see Table 6)

9.2: Pre-Infusion Visit (Day -1)

Participants enrolled based on the screening data will arrive to the hospital within 24 hours prior to gene transfer. They will undergo the following evaluations:

- Medical History
- Adverse event review
- A complete physical examination will include but may not be limited to the following systems: general, head and neck, eyes, ears and throat, chest, lungs, heart/pulse, abdomen, and skin
- Neurologic exam will include but may not be limited to the testing of cranial nerves, muscle bulk/tone and strength, sensation, cerebellar function, involuntary movements, myotatic reflexes, toe sign, gait, and stance
- Vital signs (temperature, heart rate, respiratory rate, blood pressure, oxygen saturation)
- Weight
- Concomitant medications review
- Labs will be obtained, including:
  - CBC with differential
  - Electrolytes
  - Total calcium
  - Serum total protein
  - Albumin
  - PT/INR, PTT
  - Creatinine
  - BUN
  - AST, ALT, GGT
  - Alkaline phosphatase

- Amylase
- Lipase
- Serum total bilirubin
- Serum glucose
- Beta hCG pregnancy test (females of child bearing age)

9.3: Day of Gene Transfer (Day 0)

Each participant will be admitted to the hospital the day prior to gene transfer. Prior to vector infusion, each subject will undergo the following evaluations:

- Adverse event review
- A complete physical examination will include but may not be limited to the following systems: general, head and neck, eyes, ears and throat, chest, lungs, heart/pulse, abdomen, and skin
- Concomitant medication review
- Vital signs (temperature, heart rate, respiratory rate, blood pressure, oxygen saturation)
- Participants will receive a loading dose of IV methylprednisolone (10 mg/kg to a maximum single dose of 500 milligrams, infused over 30 minutes). They will also be premedicated with enteral diphenhydramine and acetaminophen.
- Spinal needle placement will be confirmed using a fluoroscopic intraoperative imaging (C-Arm) scanner at the chosen injection site prior to and after vector administration
- Lumbar puncture: CSF will be collected prior to injection of the vector product. CSF analysis will be done which will include protein, glucose, culture, gram stain, and cell count with differential.

9.3.1: Gene Transfer Procedures

An independent assessment will be performed by Children's Health providers per local procedures with vitals collected for clinical purposes to ensure the subject is cleared for sedation.

If the subject appears inadequately hydrated in the judgment of the PI, bolus(es) of 10-20 mL/kg normal saline may be given during the time between participant check-in and gene transfer. The lowest level of sedation required will be used, and sedation will occur at least 48 hours after Screening/Baseline MRI. Participants will be continued on their usual diet until eight hours prior to gene transfer, after which they will have no solid food. Clear liquids will be allowed as per institutional guidelines for sedation, based on age. They will resume their usual diet after they have returned to pre-sedation baseline.

The study agent, AAV9/CLN7 vials, will be formulated as a concentrated stock in phosphate buffered saline containing 5% D-sorbitol and 0.001% F-68, stored at ≤ -80°C until the day of the administration procedure(s). The solution will be thawed within 4 hours of administration and diluted to the appropriate final dosage concentration and volume using phosphate-buffered saline with 5% D-sorbitol and 0.001% F-68 (if needed).

Gene transfer will be performed under sterile conditions in an appropriate procedure suite with an anesthesiologist or qualified physician present to administer sedation as needed.

9.3.2: Dosing

The stated doses are for participants ≥4 years of age and will be scaled down for younger participants as appropriate. The first participant will receive a low dose of $5x10^{14}$ vg, and following safety and tolerability assessments, subsequent participants will receive a higher dose of $1x10^{15}$ vg in 10 mL (or a lower dose in younger participants according to Table 3).

9.3.3: Intrathecal Administration

The intrathecal (IT) injection will be delivered in a constant volume (scaled with age and/or brain volume according to Table 3). A sprotte needle will be inserted percutaneously at the lumbar level into the ITspace of the spinal column with placement verified via imaging. A volume of CSF approximately equal to the infusion volume will be withdrawn from the thecal sac and will be sent for standard evaluations which will include cell count with differential, glucose, protein, culture, and gram stain. The vector solution will then be infused at a rate of 1 mL per minute. The participant will remain in 15-degree Trendelenburg position (head down) for 1 hour, during which the patient will be turned every 15 minutes, following vector administration to promote distribution throughout the CSF space.

9.3.4: Infusion Reactions

The Common Terminology Criteria for Adverse Events (CTCAE) Version 5.0 provides a grading system that is used to categorize the severity of adverse events, as follows:

- Grade 1 Mild: transient, requires no special treatment or intervention, does not interfere with daily activities
- Grade 2 Moderate: alleviated with simple treatments, may limit daily activities
- Grade 3 Severe: requires therapeutic intervention and interrupts daily activities
- Grade 4 Life-threatening or disabling
- Grade 5 Death

Infusion will be terminated for evidence of an allergic reaction of Grade 2 or greater. Under CTCAE v.5 criteria, anaphylaxis is Grade 3 (symptomatic bronchospasm with or without urticaria; allergy-related edema/angioedema; hypotension), and would result in infusion termination and systemic treatment.

Participants will remain in a PICU bed following gene transfer and remain admitted to the hospital for at least 24 hours. Vital signs will be obtained every 15 minutes for the first two hours post-infusion, 30 minutes for the third and fourth hours post-infusion, then hourly for 4 hours following the injection, and finally every 4 hours until discharge.Transfer out of the PICU to a step-down inpatient unit may be undertaken after the initial 24 hours of post-infusion monitoring if there are no medical safety concerns in the opinion of the PI.

9.4: Post-Gene Transfer Monitoring

Monitoring and follow up for safety and efficacy will be done in two parts, referred to as Period A and Period B in this document. Period A will include safety and short-term efficacy assessments. In Period A participants will be tested at screening/baseline (-28 to -7 days), early inpatient monitoring for 48 hours after AAV9/CLN7 dosing, and return for outpatient follow-up visits on Days 7, 30, 60, 90, 180, 270, 360, 540, and 720. In addition, there will be lab visits every week during the first month and every other week during the second and third month after dosing (see Table 8 for complete visit schedule). Participants will also undergo MRI brain on Days 90, 180, 360, 540 and 720 and LP with CSF analysis as safety assessments on Days 90, 180, 360 and 720. In Period B, participants will be assessed at annual visits for five (5) years after completion of Period A for long-term safety, as per FDA guidance for gene therapy studies, and efficacy assessments. During this period in between annual visits, participants/parents/guardians will be encouraged to contact the study team for any suspected adverse event reporting. Unscheduled visits may occur if the PI determines they are necessary to assess safety, repeat labs, etc.

9.4.1: Dose De-Escalation Plan

The DSMB will review all labs and safety data from the first participant receiving the low dose of $5x10^{14}$ vg at Day 30, before initiating dosing of a second participant on the higher dose of $1x10^{15}$ vg. Similarly, the third participant will not be dosed until safety data from the second participant has been reviewed by the DSMB at 30 days post-injection.

Thirty days following the dosing of the third participant, data will be reviewed by the DSMB to determine if further participants will be enrolled utilizing the higher dose of $1x10^{15}$ vg.

If the DSMB deems that the dose should be decreased for further participants, then the dose will be decreased to 5 $x10^{14}$ vg. A dose comparable to this (scaled to mice by CSF volume) was able to confer a benefit to CLN7 mice, albeit a lower benefit than the higher dose.

9.4.2: Immunomodulation Taper Plan

9.4.2.1: Steroid Taper

Participants will be on maintenance prednisone/prednisolone for 90 days. The steroid taper will be started after the Day 90 labs are completed and are deemed acceptable by the PI.

The weeks numbered below are weeks in taper schedule:

9.4.2.1.1: For steroid dose below 60 mg

At the day 90 visit, the dose will be incrementally decreased by 15% of the total dose each week. The numbers will be rounded in 2.5 mg increments.

At the Day 90 visit, safety labs and ELISpot will be checked. Once lab results have been reviewed and if normal, the steroid taper will begin as indicated below.

Week 1: Prednisone/Prednisolone will be reduced by 15% of the baseline dose.
Week 2: Prednisone/Prednisolone will be reduced by another 15% of the baseline dose.
Week 3: Prednisone/Prednisolone will be reduced by another 15% of the baseline dose.
Week 4: Prednisone/Prednisolone will be reduced by another 15% of the baseline dose.
Week 5: Prednisone/Prednisolone will be reduced by another 15% of the baseline dose.
Week 6: Prednisone/Prednisolone will be reduced by another 15% of the baseline dose

The taper will continue until the participant reaches 5 mg, at which time tapering will be stopped temporarily. If the last dose is above 5 mg, it will be rounded down to 5 mg between Days 120-134. After participants are on a 5 mg dose for 1 week, an 8 AM cortisol level would be checked. If the 8 AM cortisol level is normal, prednisone/prednisolone will be tapered off in 1 mg increments every 4-7 days. All participants will be off steroids by Day 150. At Day 180, scheduled safety and immunomodulation labs will be checked.

9.4.2.1.2: For steroid dose at or above 60 mg

At the Day 90 visit, safety labs and ELISpot will be checked. Once lab results have been reviewed and if normal, the steroid taper will begin as indicated below.

Week 1: Prednisone/ Prednisolone will be reduced to 40 mg/day.
Week 2: Prednisone/ Prednisolone will be reduced to 30 mg/day.
Week 3: Prednisone/ Prednisolone will be reduced to 20 mg/day.
Week 4: Prednisone/ Prednisolone will be reduced to 10 mg/day.
Week 5: Prednisone/ Prednisolone will be reduced to 5 mg/day.

All participants will continue at a 5 mg dose for one week. At this point, an 8 AM cortisol level will be checked. If the 8 AM cortisol level is normal, prednisone/prednisolone will be tapered in 1 mg increments every

4-7 days. All participants be off steroids by Day 150. At Day 180, scheduled safety and immunomodulation labs will be checked.

If safety lab results are not normal at any point during the time participants are on maintenance steroids, prednisone/prednisolone dose may be increased to 2 mg/kg/day (max daily dose 60mg) at the PI's discretion, depending on the T-cell response measured by ELISpot assay specifically to the CLN7 protein. The PI might also decide to prolong the tapering protocol based on the individual participant's immune response to the gene transfer, again assessed by ELISpot assay specifically for the CLN7 protein.

9.4.2.2: Sirolimus Taper

The sirolimus taper will be started after the Day 270 labs are normal. The taper will start no later than 10 months. The taper will complete over the following 4-6 weeks. On Day 360 visit, safety labs will be checked.

9.4.2.3: Tacrolimus Taper

The tacrolimus taper will be started after Day 180 if labs are normal. Taper will complete over next 4-6 weeks. On Day 270, safety labs will be checked.

9.4.3: Early Inpatient Monitoring (up to 48 hours of AAV9/CLN7 dose)

The subject will be evaluated by the PI or designee on the morning of Day 1 following vector administration for adverse events, vital signs, repeat physical/neurologic examination, and review safety laboratory studies. Participants will also be on telemetry per PICU protocol. Participants will be monitored in PICU for the first 24 (+/- 4) hours after the vector administration and will be transferred to a pediatric floor after the initial 24 hours if no medical complications are noted. Enteral prednisone or prednisolone will be administered as outlined in the immunomodulation regimen. Participants will remain on the pediatric floor for continued monitoring another 24 (+/- 4) hours, then will be discharged if there are no complications.

Participants will undergo the following evaluations:

- Medical History
- Adverse event review
- A complete physical examination will include but may not be limited to the following systems: general, head and neck, eyes, ears and throat, chest, lungs, heart/pulse, abdomen, and skin
- Safety neurologic checks will be completed every 4 hours for the first 24 (+/-4) hours post-infusion and will include mental status, cranial nerve examination, muscle strength, sensory exam to assess response to external stimuli, and myotatic reflexes and toe sign
- Neurologic exam on discharge will include testing of cranial nerves, muscle bulk/tone and strength, sensation, cerebellar function, involuntary movements, myotatic reflexes, toe sign, gait and stance
- Vital signs (temperature, heart rate, respiratory rate, blood pressure, oxygen saturation)
- Weight
- Concomitant medications review
- Samples for future vector shedding analysis (plasma, urine, saliva, and if available feces)
- Safety Labs will be obtained, including:
  - CBC with differential
  - Electrolytes
  - Total calcium
  - Serum total protein
  - Albumin
  - PT/INR, PTT
  - Creatinine

- BUN
- AST, ALT, GGT
- Alkaline phosphatase
- Amylase
- Lipase
- Serum total bilirubin
- Serum glucose
- Capillary blood gases

Administration of enteral prednisone/prednisolone, along with sirolimus, will be continued following discharge and will then be tapered according to liver function testing and IFN-gamma T-cell studies in the following weeks. If post-infusion IFN-gamma T-cell responses remain ≤50 SFC per $1x10^6$ PBMCs, the participant's prednisone/prednisolone dose will begin to be tapered around Day 90. Tapering will proceed slowly, typically over 4 to 7 weeks. For tapering, if either the AST and ALT exceeds >2.5X the upper normal limit and results are confirmed on a follow-up blood test within three days, the prednisone/prednisolone regimen will be maintained at 1 mg/kg/day until the enzyme levels fall below these levels. Similarly, sirolimus will be tapered starting at 10 months post-injection (see Section 9.4.2). Tacrolimus will be added to the immune modulation regimen for patients with null mutation. At any time at the discretion of the the PI, additional labs may be drawn.

The steroid dose may be increased to approximately 2 mg/kg/day, depending on the T-cell response measured by ELISpot assay, and prolong the tapering protocol based on the individual participant's immune response to the gene transfer. Liver function testing (AST, ALT, GGT) will be repeated 2 weeks after the last dose of steroid. Should the participate experience an acute neuroinflamatory response, the management may include pulsed high dose IV methylprednisolone per PI discretion.
The participant will be evaluated by the PI or designee on the morning of Day 2 for repeat physical examination. Enteral prednisone/prednisolone, sirolimus, and tacrolimus (if indicated) will be administered. The participant will be discharged from the hospital if the physical examination and laboratory studies do not indicate a need for further inpatient care.

9.4.4: Outpatient Follow-up Visits (Day 7, 30, 60, 90, 180, 270, 360, 540, and 720)

The subject will be evaluated by the PI or designee in an outpatient setting. They will undergo the following evaluations:

- Medical history
- Adverse events
- A complete physical examination will include but may not be limited to the following systems: general, head and neck, eyes, ears and throat, chest lungs, heart, pulses, abdomen and skin
- Neurologic exam will include testing of cranial nerves, muscle bulk/tone and strength, sensation, cerebellar function, involuntary movements, myotatic reflexes, toe sign, gait and stance
- Vital signs (temperature, heart rate, respiratory rate, blood pressure, oxygen saturation)
- Weight
- Concomitant medications will be reviewed
- ELISA for detection of serum antibodies to AAV9
- ELISpot for T cell responses to AAV9 and CLN7
- Labs will be obtained, including:
  - CBC with differential
  - Electrolytes
  - Serum total protein
  - Total calcium
  - Albumin
  - PT/ INR, PTT

  - Creatinine
  - BUN
  - AST, ALT, GGT
  - Alkaline phosphatase
  - Amylase
  - Lipase
  - Serum total bilirubin
  - Serum glucose
- Collection of exploratory samples to include plasma, urine, saliva, and feces for future analyses, including vector shedding
- Secondary outcome assessments of disease burden and product efficacy (see Table 1) will be completed on Days 180, 360, 540, and 720

9.4.5: Additional Visits for Follow-up Lab Monitoring

In addition to the above-mentioned visits, labs will be drawn for safety monitoring every week for the first month and every other week for the second and third months to include the following:

- CBC with differential
- Electrolytes
- Serum total protein
- Total calcium
- Albumin
- PT/ INR, PTT
- Creatinine
- BUN
- AST, ALT, GGT
- Alkaline phosphatase
- Amylase
- Lipase
- Serum total bilirubin
- Serum glucose

9.4.6: Follow-up MRI Brain

MRI of the brain (with or without contrast) will be obtained on Days 90, 180, 360, 540, and 720. Additional imaging may be required and will be obtained based upon the PI's clinical assessment (e.g., if participant has any new neurologic symptoms not consistent with disease progression of CLN7 deficiency, and/or unexpected worsening of previous neurologic symptoms).

9.4.7: Follow-up Lumbar Puncture

LP will be obtained on Days 90, 180, 360, and 720. This is to monitor the development of CSF pleocytosis which may warrant a change in immunomodulation therapy. CSF analyses will include protein, glucose, culture, gram stain, and cell count with differential.

9.5: Long-Term Monitoring

We will follow the most recent FDA guidelines regarding long-term participant follow up after gene transfer. As indicated by the guidelines, the proposed vector has a very low probability of gene transfer-related adverse events. We will, however, evaluate short-term safety over a two-year period that incorporates the active phase of the protocol. Following the active two-year follow-up phase of the study, participants will then transfer to a long-term

monitoring program where data will continue to be collected from annual visits with the study team during Period B of follow-up. The study team will request medical records as indicated.

In addition, participants/parents/guardians will be encouraged to contact the study team for any suspected adverse event reporting between visits. Unscheduled visits may occur if the PI determines they are necessary to assess safety, repeat labs, etc. The following information will be requested to be collected annually on standard case report forms (CRFs) and submitted to UTSW as part of the annual monitoring program:

- Review of any medical issues, pain, or discomfort.
- Review of body systems.
- Medications.
- Survey of behavioral and day-to-day function.

If newly identified risks are associated with this agent, or if the participants suffer any adverse events during this period, we will initiate a long-term follow-up according to the FDA guidelines. The proposed schedule of events is described below:

Table 7: Five Year Follow-Up (Period B)

Day	1 (±14)	360, 720, 1080, 1440, 1800 (±14)
Informed consent review	X	
Review of medical issues, pain, discomfort		X
Review of body systems		X
Survey of behavioral and day-to-day function		X
Adverse events		X
Concomitant medications		X

9.6: Outcome Measures

9.6.1: Safety Measures

The primary outcome consists of safety and tolerability of the investigational agent, including clinically significant changes from baseline. Measurements that will be used for the evaluation of toxicity for 24 months of follow-up will include:

- Adverse events and concomitant medication
- Routine laboratory tests (hematology, chemistry, and urinalysis)
- Liver function (AST, ALT, total bilirubin, prothrombin time) weekly for the first month; every other week for the second and third months, or until results are unremarkable (total bilirubin, and prothrombin results, and ALT and AST levels below 2 × ULN). For persistents abnormal LFTs, prolonged monitoring may be required at the discretion of the PI.
- Platelet counts weekly for the first month, and then every other week for the second and third months, or until platelet counts return to baseline
- Vital signs (temperature, heart rate, respiratory rate, blood pressure, oxygen saturation)
- Physical examination
- 12-lead electrocardiogram (ECG)

- Immunogenicity: Serum neutralizing antibodies against AAV9, ELISpot results for T-cell reactivity to AAV9 and CLN7
- Incidence of drug-related adverse events

9.6.2: Secondary/Exploratory Efficacy Endpoints

Endpoint	Assessments
Disease burden	1. Clinical Global Impression
Ataxia and motor function assessments:	1. 2-Minute Walk Test OR 6-Minute Walk Test<br>2. Pediatric Balance Scale<br>3. Gross Motor Function Measure
Intelligence and cognition assessments	1. Mullen<br>2. Vineland Adaptive Behavior Scales, 3rd Edition
Quality of life measurement:	1. Quality of Life Inventory-Disability (QI-Disability)<br>2. ITQOL
Surrogate measures	1. EEG<br>2. MRI Brain

9.6.3: Statistical Analysis

Data collected from this study will be presented in aggregate. Rates of adverse events and serious adverse events will be reported by category. Given the small sample size, analytic statistics at a population level will not be possible. Descriptive statistics will be provided. Furthermore, rates of change in disability scores per patient will be provided, but the study is not powered to discern the effect size of the therapy.

10.0 Data Monitoring

10.1: General Plan

For this Phase I study of Gene Therapy the safety oversight will be focused not only on the initial treatment but also on an extended observation. The trial will utilize a DSMB and an independent data monitoring to complement the safety responsibilities of the investigators (see below).

10.2: Monitoring Entity

Dr. Benjamin Greenberg (UT Southwestern) is the lead investigator and will be responsible for assuring ongoing safety monitoring of the trial.

Members on the DSMB will serve as independent safety reviewers. The DSMB charter defines the role and responsibilities of the members of the group, and contains assurances of freedom from conflicts of interest.

UTSW Human Research Protection Program Office (HRPPO) will provide data monitoring. The ongoing data monitoring responsibilities are contracted by the UTSW PI, with the UTSW HRPPO on a fee-for-service basis to monitor the study progress and will function independently from the study team. The UTSW PI is responsible for providing copies of the HRPPO monitoring reports, and disclosing any reportable events (REs) submitted to the UTSW HRPPO/IRB to the FDA in accordance with federal requirements.

10.3: Plans for Assuring Participant Safety, Adverse Event Collection, and Reporting

Expected adverse events could be due to the infusion and are listed in Section 9.3.4. Other adverse events could be due to immunomodulatory drugs.

Maximum efforts will be undertaken to ensure the safety of all study participants.

The primary and secondary endpoint assessment is identifying the safety and tolerability of intrathecal administration of AAV9/CLN7. Monitoring for safety will be performed by recording and evaluating type and occurrences of Adverse Events (AEs), concomitant medication usage, and by conducting physical examinations, vital sign assessments, cardiovascular evaluations, and laboratory evaluations (chemistry, hematology, coagulation, immunology).

Further plans are described in Sections 10.4 through 10.11.

10.4: Definitions

10.4.1: Adverse Event

Adverse events (AE) are defined as any untoward occurrence associated with the use of an intervention in humans, whether or not considered intervention-related (21CFR 312.32. a). These events will be reviewed by the PI and determined if they are clinically significant requiring adjustments to medications or interventions. All AEs occurring from the time of informed consent will be documented, recorded, and reported within 7 days to the Monitoring Entities following PI notification.

Clinically significant signs and symptoms or lab abnormalities will be recorded as an AE. A laboratory abnormality should be reported as an AE if it requires an intervention. Interventions include, but are not limited to, discontinuation of treatment, dose changes of medications, additional assessments, or concomitant treatment. In addition, any medically important laboratory abnormality may be reported as an AE at the discretion of the PI. This could include a laboratory result for which there is no intervention, but the abnormal value suggests a disease or organ toxicity. The PI will evaluate all AEs with respect to Seriousness, Severity (intensity or grade), and Causality (relationship to study agent and relationship to research) according to the following guidelines. All AEs will be classified in accordance with the CTCAE v.5. AEs will be coded in accordance with the most current version of the MedDRA coding dictionary.

All events will then be reviewed by the DSMB and an evaluation will be made as to whether the study should be terminated early following the discontinuation rules.

10.4.2: Classification of Adverse Events

Monitoring AEs requires that they be classified as to seriousness, expectedness, and potential relationship to the study drugs, all of which drive the reporting process.

10.4.3: Seriousness

A serious adverse event (SAE) is one that:

- Results in death,
- Is life-threatening (the participant was in immediate danger of death from the event as it occurred),
- Requires inpatient hospitalization or prolongation of existing hospitalization,

- Results in persistent or significant disability/incapacity, or
- Is a congenital anomaly/birth defect in the offspring of a participant.

All SAEs that occur after any patient has been enrolled, before vector dosing, during vector dosing, or up through the last study visit, whether or not they are related to the study, must be recorded on case report forms. All SAEs will be required to be reported to the IRB within 24 hours of it being brought to the attention of the PI.

CTCAE v.5 provides a grading system that is used to categorize the severity of adverse events, as follows:

- Grade 1 Mild: transient, requires no special treatment or intervention, does not interfere with daily activities
- Grade 2 Moderate: alleviated with simple treatments, may limit daily activities
- Grade 3 Severe: requires therapeutic intervention and interrupts daily activities
- Grade 4 Life-threatening or disabling
- Grade 5 Death

An SAE, as defined above, encompasses CTCAE grades 4 and 5, and any Grade 3 event that requires or prolongs hospitalization, or that is disabling. Other SAEs that are considered Important Medical Events (IME) requiring medical judgement that need reporting is when the event does not fit the outcomes listed above, but the event may jeopardize the patient and may require medical or surgical intervention (treatment) to prevent one of the other outcomes.

<u>10.4.4: Expectedness</u>

The purpose of reporting is to provide new, important information on serious reactions or events previously unobserved or undocumented. Therefore, all AEs will be evaluated as to the expectedness of its occurrence as follows:

- Unexpected: An unexpected AE or adverse drug reaction is one for which the nature or severity is not consistent with information in the protocol, consent form, or product brochure.
- Expected: An AE is considered expected if it is known to be associated with the study intervention. Expected adverse events <u>due to underlying disease</u> are listed below:
    - Worsening of ataxia
    - Worsening of vision
    - Worsening seizures and myoclonus
    - Status epilepticus/status myoclonus
    - Progressive atrophy in brain noted in follow-up MRIs
    - Progression of dysphagia and eventual need of a G button
    - Patients who are G button dependent can have complications with displacement of G button or G button leak

<u>10.4.5: Causality</u>

Causality assessment is required in clinical investigations to help determine which events require expedited reporting. The PI must make the determination of relationship to the investigational product for each AE (Unrelated, Possibly Related, Probably Related, or Definitely Related). The PI should decide whether, in his/her medical judgment, there is a reasonable possibility that the event may have been caused by the investigational product. If no valid reason exists for suggesting a relationship, then the AE should be classified as "unrelated." If there is any valid reason, even if undetermined, for suspecting a possible causative relationship between the investigational product and the occurrence of the AE, then the AE should be considered "related." If the relationship between the AE/SAE and the investigational product is determined to be "possible" or "probable", the event will be considered to be related to the investigational product for the purposes of expedited regulatory reporting.

The following criteria will be used to determine causality:

- Unrelated: The event is clearly related to other factors, such as the participant's clinical state or non-study drugs or interventions.
- Possibly Related: The event follows a compatible temporal sequence from the time of administration of the study agent, but could have been produced by other factors such as the participant's clinical state or non-study drugs or interventions.
- Probably Related: The event follows a reasonable temporal sequence from the time of study agent administration, and cannot be reasonably explained by other factors such as the participant's clinical state or non-study drugs or interventions.

<u>10.4.6: Dose Limiting Toxicity</u>

Dose limiting toxicity (DLT) is defined as any SAE that is possibly, probably, or definitely related to the study agent. This would include any Grade 3 or greater event, according to the CTCAE v.5; these classifications are outlined below:

- Grade 1 Mild: transient, requires no special treatment or intervention, does not interfere with daily activities
- Grade 2 Moderate: alleviated with simple treatments, may limit daily activities
- Grade 3 Severe: requires therapeutic intervention and interrupts daily activities
- Grade 4 Life-threatening or disabling
- Grade 5 Death

Study enrollment will be halted by the investigators when any subject experiences a Grade 3 or higher AE that is unanticipated and possibly, probably, or definitely related to the investigational product. The event will then be reviewed by the DSMB and an evaluation will be made as to whether the trial should be terminated early following the discontinuation rules, or if a protocol modification should be considered.

Discontinuation Rules:
Study enrollment will be halted by the investigators when any subject experiences a Grade 3 or higher AE that is unanticipated and possibly, probably, or definitely related to the investigational product that presents with clinical symptoms and requires medical treatment. This will include any patient death, important clinical laboratory finding, or any severe local complication in the injected area related to administration of the study agent. If after review by the DSMB, IRB and FDA, the decision is made to continue, the study will proceed with a lower dose per the dose de-escalation plan.

<u>10.4.7: Other Adverse Events</u>

Other adverse events (OAEs) may be identified by the PI and the DSMB. Significant AEs of particular clinical importance, other than SAEs and those AEs leading to discontinuation of the participant from the study, will be classified as OAEs.

<u>10.5: Reporting Procedures to the DSMB</u>

The DSMB will have access to real-time review of participant data during the course of the study through access to the participants electronic medical record. In addition, following the dosing of the first participant, a detailed review of safety will be conducted by the DSMB prior to dosing subsequent participants. After 30 days following infusion of each participant, the PI will submit a safety report to the DSMB for review. Requests for additional data by the DSMB can be made by communicating the request to the PI.

Other reports submitted will include:

- Immediate and interim subject data reviews. Data for individual subject reviews or for SAEs will be made available as soon as possible
- Summaries of adverse events. (SAEs and AEs)
- Information necessary to review the conduct of the trial, including recruitment, enrollment, and unexpected problems
- Information from nonclinical findings that may impact the safety assessment of the trial will be provided by the PI or his designee
- The reports to the DSMB are considered privileged and not subject to disclosure except as required by law
- Ad hoc data summaries may be prepared upon written request by the DSMB to address a specific safety concern (email is an acceptable method of communication)

10.6: Reporting Procedures to the FDA

As the PI is the sponsor -Investigator, the PI will notify the FDA of potential serious risks, from clinical trials or any other source, as soon as possible, but in no case later than 15 calendar days after the PI determines that the information qualifies for reporting (see below) per 21CFR312.32. In each IND safety report, the PI will identify all IND safety reports previously submitted to FDA concerning a similar suspected adverse reaction, and must analyze the significance of the suspected adverse reaction in light of previous, similar reports or any other relevant information.

Information that qualifies reporting:

- *Serious and unexpected suspected adverse reaction.* The PI will report any suspected adverse reaction that is both serious and unexpected. The PI will report an adverse event as a suspected adverse reaction only if there is evidence to suggest a causal relationship between the drug and the adverse event, such as
    - single occurrence of an event that is uncommon and known to be strongly associated with drug exposure.
    - One or more occurrences of an event that is not commonly associated with drug exposure, but is otherwise uncommon in the population exposed to the drug.
    - An aggregate analysis of specific events observed in a clinical trial (such as known consequences of the underlying disease or condition under investigation or other events that commonly occur in the study population independent of drug therapy) that indicates those events occur more frequently following treatment.

- *Findings from other studies.* The PI will report any findings from epidemiological studies, pooled analysis of multiple studies, or clinical studies that suggest a significant risk in humans exposed to the drug.
- *Findings from animal or in vitro testing.* The PI will report any findings from animal or in vitro testing, whether or not conducted by the sponsor, that suggest a significant risk in humans exposed to the drug.
- *Increased rate of occurrence of serious suspected adverse reactions.* The sponsor must report any clinically important increase in the rate of a serious suspected adverse reaction over that listed in the protocol or investigator brochure.
- *Submission of IND safety reports.* The PI will submit each IND safety report in a narrative format or on FDA Form 3500A or in an electronic format that FDA can process, review, and archive. Reports of overall findings or pooled analyses from published and unpublished in vitro, animal, epidemiological, or clinical studies must be submitted in a narrative format.
- *Unexpected fatal or life-threatening suspected adverse reaction reports.* The PI will also notify FDA of any unexpected fatal or life-threatening suspected adverse reaction as soon as possible but in no case later than 7 calendar days after the PI's initial receipt of the information.

10.7: Reporting Procedures to the IRB

Unanticipated problems involving risks to subjects or others (UPIRSO) will be reported to the IRB within 5 working days of discovery if they follow the following definition:

An event that meets ALL three (3) of the following criteria: 1. Unexpected (in nature, severity, or frequency), AND 2. Probably or definitely related to the research, AND 3. Suggests the research places subjects or others at a greater risk of harm than previously known or recognized.

All other research-related events and reports will be summarized at annual continuing review (CR) or notice of study closure, whichever comes first. That includes, but is not limited to: • Noncompliance events (e.g., deviations) that do not meet the UTSW HRPP definition of either serious or continuing noncompliance • AEs/SAEs that do not meet ALL 3 UPIRSO criteria • Events/reports the sponsor wants submitted to the UTSW IRB/HRPP • Data safety monitoring (DSMB) reports • Other safety reports (e.g. IND) • Monitoring/audit reports • Any other new information since the last IRB/HRPP review

A Summary Report of AEs will be prepared by the PI annually and will be sent to the IRB at continuing review. The Summary Report will contain the following information:

- A statement that for DSMB review of outcome data, AEs, and information relating to study performance took place on a given date.
- A statement as to whether or not the frequency of AEs exceeded what was expected and indicated in the informed consent.
- The DSMB recommendation to either proceed with the study or modify the protocol or informed consent document. If the DSMB recommends changes to the protocols or informed consent document, the rationale for such changes and any relevant data will be provided.
- A statement that if safety concerns are identified, they will be communicated promptly to the investigators.

10.8: Protocol Deviations and Continuing Review

Deviations to the protocol will not be reported to the IRB unless they occur at a rate greater than anticipated by the study team. If the rate of these events exceeds the rate expected by the study team, the events will be classified and reported as though they are unanticipated problems.

The following items will be reported to the UTSW IRB in summary at the time of Continuing Review:

- Serious and non-serious unanticipated problems,
- Expected serious adverse events that are possibly, probably, or definitely related to the research,
- Serious adverse events that are not related to the research,
- All adverse events, except expected AEs and deaths granted a waiver of reporting,
- Serious and non-serious protocol deviations,
- Serious, continuing, and minor non-compliance,
- Any trends or events which in the opinion of the investigator should be reported, and
- Any protocol-specific reporting requirements (as applicable).

10.9: Stopping Rules

The DSMB will have the responsibility and authority to stop or suspend the trial based on their review of the data and the pre-determined stopping rules. If DSMB determines that the trial should be stopped or suspended, they will notify Dr. Greenberg as soon as feasible. Dr. Greenberg will be responsible for notifying the IRB within three days.

The DSMB will review the study data that the PI will submit listed below:

- A narrative summary of trial activity to date,
- A line listing of all AEs reportable per protocol,
- A narrative summary assessment of any safety concerns including:
  - AE and SAE trends,
  - Unanticipated Problems relating directly to protocol-driven activities,
  - Participants withdrawn for safety reasons,
  - Trial halting or pausing activity, and
  - Other events relating to the overall safety of the trial.

After each DSMB review, a recommendation as to whether the study is to continue, be modified, or be terminated will be provided in a summary report. All SAEs, all unanticipated problems, and all IND Safety Reports will be reported by the PI to the DSMB at the same time they are submitted to the IRB. The DSMB will be notified immediately if pausing or halting rules are met and the DSMB will provide a recommendation for continuation, modification, or termination of the study. The PI will submit the written DSMB summary reports with recommendations to the IRB.

Halting the study requires immediate discontinuation of study agent administered for all participants and suspension of enrollment until a decision is made whether or not to continue study agent administration.

Halting Criteria for the Protocol:

- Two or more participants experience the same or similar SAEs that are unexpected and are possibly, probably, or definitely related to the study agent.
- Three or more of the same or similar AEs in different subjects that are grade 3 or above and are unexpected and possibly, probably, or definitely related to the study agent.
- Any safety issue that the site investigators determine should halt the study.

Reporting of Study Halting:
If a halting requirement is met, a description of the event(s) or safety issue must be reported by the PI within one business day by fax or email AND the PI must inform the IRB that a halting rule has been met.

Resumption of a Halted Study:
PI-Sponsor and the DSMB will determine if it is safe to resume the study. The conditions for resumption of the study will be defined in a notification. The PI will notify the IRB of the decision to resume the study.

<u>10.10: Data Collection</u>

Data will be collected at specified time intervals as outlined in the protocol. Once the participant and/or parent/guardian has signed the informed consent/assent form, data can then be collected, including pertinent retrospective medical records per PI discretion.

Source data is all information, original records of clinical findings, observations, or other activities in a clinical trial necessary for the reconstruction and evaluation of the trial. Source data are contained in source documents. Examples of these original documents, and data records include: hospital records, clinical and office charts, pathology reports, laboratory notes, memoranda, participants' diaries or evaluation checklists, pharmacy dispensing records, recorded data from automated instruments, copies or transcriptions certified after verification as being accurate and complete, microfiches, photographic negatives, microfilm or magnetic media, digitized imaging data, x-rays, participant files, and records kept at the pharmacy, at the laboratories, and at medico-technical departments involved in the clinical trial.

The study electronic case report forms (eCRF) is where all data collection will be inputted for the study. All data requested on the eCRF will be recorded by the clinical operations team consisting of the clinical research coordinators/managers and research nurses. The electronic data capture (EDC) platform used in this clinical trial will be REDCap. REDCap is a secure web-based application that is FDA compliant per 21 CFR11 regulation. It supports regulatory trials to ensure Good Clinical Practice (GCP). All missing data must be explained, and it will have automatic data verification in place to ensure complete and accurate data is entered. REDCap will be accessed via a secure personalized login, thus allowing for role-appropriate access, and providing audit trails for data entry, exports, and reports. Trial data will be entered using participant identification numbers. PHI will not be shared outside of IRB approved entities.

10.10.1: Database Locks

For key deliverables requiring analysis of the trial data, an export of the entire database from REDCap will be performed at such periodic intervals in order to have a locked dataset from which all results will be generated. At trial end, a final lock and export will occur after all data queries are resolved and statistical analysis will be performed. The final trial results, FDA summary report, and publications will be prepared from this locked dataset. The study database will be retained at the site 5 years after trial end.

10.10.2: Study Monitoring Plan

This study will be monitored according to the outline in the protocol. The PI will allocate adequate time for such monitoring activities. The data entered into REDCap will be reviewed and verified for accuracy by the Investigator.

The DSMB will monitor the safety data for this study and are considered experts in rare neurological disease, bioethics, and trial design. They will have access to patient data in real-time during screening, dosing and the post-dosing monitoring period. On a planned periodic basis, the PI will submit a report generated from the EDC, which will include information on accrual rate, data collected on each participant, data quality, and data completeness as agreed by the DSMB charter. The focus of these initial reviews/reports will be primary safety and AE rates. In addition, the DSMB will regularly review serious AEs and protocol deviations associated with the research to ensure the protection of human subjects.

UTSW Human Research Protection Program Office (HRPPO) will also provide data monitoring. The ongoing data monitoring responsibilities are contracted by the UTSW PI, Dr. Greenberg, with the UTSW HRPP Office (HRPPO) on a fee-for-service basis to monitor the study progress and will function independently from the study team. The PI is responsible for providing copies of the HRPPO monitoring reports and disclosing any reportable events (REs) submitted to the UTSW HRPPO/IRB to the FDA in accordance with federal requirements. The PI will also ensure that the UTSW IRB or other compliance/quality assurance reviewers are given access to all the above noted study-related documents and study-related facilities and has adequate space to conduct monitoring visits. The PI will permit study-related monitoring, audits, and inspections by the government regulatory bodies such as the FDA. Should any issues arise, a corrective and preventive action plan (CAPA) will be deployed to facilitate improvement with respect to performance issues.

10.10.3: Quality Assurance of Data

Quality assurance (QA) processes are in place to ensure the data will be collected and entered into the EDC accurately and consistently. QA of trial data will employ several approaches, including auditing of CRFs to identify data fields that require adaptation for clinical relevance and accessibility, a Manual of Operations (MOO) prepared by the clinical operations team with detailed instructions for data collection and entry into EDC, and real-time validations of submitted data.

Table 8: Complete Schedule of Events (Period A)

	Pre-Screening	Screening / Baseline	Gene Transfer (inpatient)				24-Month Follow-up												
**Visit**	0	1	2				3	4*	5*	6	7*	8	9*	10	11	12	13	14	15
Days in study	Before -30	-28 to -7	-1	0	1	2	7	14	21 (±2)	30 (±2)	44 (±2)	60 (±2)	74 (±14)	90 (±14)	180 (±14)	270 (±14)	360 (±14)	540 (±14)	720 (±14)
**Eligibility**																			
Informed consent		x																	
Eligibility		x	x	x															
Informed consent review	x		x																
**Study procedure**																			
Study drug administration[1]				x															
**Clinical Assessments**																			
Medical history		x	x				x			x		x		x	x	x	x	x	x
Height,weight and head circumference		x																	
Physical Exam and vital signs[2]		x	x	x	x	x	x			x		x		x	x	x	x	x	x
Safety Neuro checks [3]				x	x	x													
Neurologic exam		x	x			x	x			x		x		x	x	x	x	x	x
CGI		x													x		x	x	x
Physiotherapy assessments		x													x		x	x	x
Neuropsychological testing		x													x		x	x	x
Quality of Life Measures		x													x		x	x	x

	Pre-Screening	Screening / Baseline	Gene Transfer (inpatient)				24-Month Follow-up												
**Visit**	0	1	2				3	4*	5*	6	7*	8	9*	10	11	12	13	14	15
Days in study	Before -30	-28 to -7	-1	0	1	2	7	14	21 (±2)	30 (±2)	44 (±2)	60 (±2)	74 (±14)	90 (±14)	180 (±14)	270 (±14)	360 (±14)	540 (±14)	720 (±14)
**Immunomodulation for CRIM+ Subjects**																			
Steroid dosing[4]				x	x	x	x	x	x	x	x	x	x	x					
Sirolimus Dosing[5]		x	x	x	x	x	x	x	x	x	x	x	x	x	x	x			
**Immunomodulation for CRIM- Subjects**																			
Steroid Dosing[4]				x	x	x	x	x	x	x	x	x	x	x					
Sirolimus Dosing[5]		x	x	x	x	x	x	x	x	x	x	x	x	x	x	x	x	x	x
Tacrolimus Dosing[6]					x	x	x	x	x	x	x	x	x	x	x				
**Labs**																			
Screening labs[7]		x																	
Molecular testing for CLN7- if not previously completed	x	x																	
Safety labs[8, 17] (blood & urine)		x	x		x	x	x	x	x	x	x	x	x	x	x	x	x	x	x
Biobanking Labs[16]		x					x			x		x		x	x	x	x	x	x
Anti-AAV9 antibody[9] titers		x					x			x		x		x	x	x	x	x	x
ELISpots for T-cell[10] responses to AAV9 and CLN7 protein		x					x			x		x		x	x	x	x	x	x
Vector shedding samples[11]		x			x		x	x		x				x	x		x	x	x

	Pre-Screening	Screening / Baseline	Gene Transfer (inpatient)				24-Month Follow-up												
**Visit**	0	1	2				3	4*	5*	6	7*	8	9*	10	11	12	13	14	15
Days in study	Before -30	-28 to -7	-1	0	1	2	7	14	21 (±2)	30 (±2)	44 (±2)	60 (±2)	74 (±14)	90 (±14)	180 (±14)	270 (±14)	360 (±14)	540 (±14)	720 (±14)
Sirolimus/ Tacrolimus Trough levels[12]							x	x	x	x	x	x	x	x	x	x	x	x	x
Lipid Panel[13]		x								x		x		x	x	x	x	x	x
**Procedures**																			
EEG-1 hour		x													x		x	x	x
ECG		x	x	x	x	x	x	x	x	x		x		x			x		x
Echocardiogram		x															x		x
MRI[15]		x												x	x		x	x	x
CSF sample[14] (LP)[15]				x										x	x		x		x
Pulse oximetry		x	x	x	x	x	x			x		x		x	x	x	x	x	x
Capillary blood gas		x	x	x	x	x													
Swallow study		x															x		x
Ophthalmologic evaluations		x															x		x
Seizure diary		x												x	x	x	x	x	x
Healthcare utilization diary		x												x	x	x	x	x	x
Adverse event and concomitant medication review		x	x	x		x	x	x	x	x	x	x	x	x	x	x	x	x	x

* Lab visits

1. Continuous monitoring during gene transfer procedure. Temperature will be captured pre- and post-infusion.
2. Vital signs will be recorded per section 6.2.3 during inpatient hospitalization. Vital signs taken include temperature, heart rate, respiratory rate, blood pressure, and pulse oximetry. The participant’s weight will also be measured on admission.
3. Patient will have neuro-checks every 4 hours post therapy for 24 hours, as needed at PI discretions and prior to discharge.
4. Please refer to immune-modulation protocol for steroid tapering. See section 9.4.2.

5. Please refer to immune-modulation protocol for sirolimus tapering. See Section 9.4.2.
6. Please refer to immune modulation protocol for Tacrolimus dosing. See Section 8.6.
7. Please refer to Section 9.1 for complete list of screening labs.
8. Please refer to Section 9.4.3 for complete list of safety labs. Please note that some of these visits are in person clinic visits and while others are solely lab visits. If participant cannot travel to the site, any inter-visit safety blood collections may be done by a local healthcare provider (and results sent to the study team). Samples that can only be processed and resulted at UTSW labs (e.g ELISpot, ELISA, vector shedding samples) will be shipped to the investigator's site for analysis.
9. Additional levels may be drawn during steroid/sirolimus taper if there are any clinical concerns, based upon PI's discretion.
10. Additional levels may be drawn during steroid/sirolimus taper if there are any clinical concerns, based upon PI's discretion.
11. Vector shedding sample collection will include urine, feces, saliva, and plasma. If samples other than baseline test negative, subsequent time points will not be tested.
12. Sirolimus/tacrolimus levels to be drawn per Immunomodulation protocol outlined in Section 8.6. Sirolimus/tacrolimus trough levels will vary after day 90 depending on when the trough levels are stable and also on CRIM status (as CRIM negative patients will be on Sirolimus indefinitely per PIs discretion).
13. Monthly lipid profile per immune modulation protocol.
14. Cerebrospinal fluid analysis includes cell count, differential protein, glucose, gram stain, culture.
15. LP/MRI may be done with or without sedation.
16. Biobanking labs include collection of blood to be processed into serum, plasma, PBMC and DNA/RNA extraction. See lab manual for full schedule.
17. Liver function tests per Section 9.6.1.

11.0 References

1. Siintola E, Topcu M, Aula N, et al. The novel neuronal ceroid lipofuscinosis gene MFSD8 encodes a putative lysosomal transporter. Am J Hum Genet 2007;81(1):136-46 doi: 10.1086/518902.
2. Aiello C, Terracciano A, Simonati A, et al. Mutations in MFSD8/CLN7 are a frequent cause of variant-late infantile neuronal ceroid lipofuscinosis. Hum Mutat 2009;30(3):E530-40 doi: 10.1002/humu.20975.
3. Kousi M, Siintola E, Dvorakova L, et al. Mutations in CLN7/MFSD8 are a common cause of variant late-infantile neuronal ceroid lipofuscinosis. Brain 2009;132(Pt 3):810-9 doi: 10.1093/brain/awn366.
4. Kousi M, Lehesjoki AE, Mole SE. Update of the mutation spectrum and clinical correlations of over 360 mutations in eight genes that underlie the neuronal ceroid lipofuscinoses. Hum Mutat 2012;33(1):42-63 doi: 10.1002/humu.21624.
5. Roosing S, van den Born LI, Sangermano R, et al. Mutations in MFSD8, encoding a lysosomal membrane protein, are associated with nonsyndromic autosomal recessive macular dystrophy. Ophthalmology 2015;122(1):170-9 doi: 10.1016/j.ophtha.2014.07.040.
6. Mandel H, Cohen Katsanelson K, Khayat M, et al. Clinico-pathological manifestations of variant late infantile neuronal ceroid lipofuscinosis (vLINCL) caused by a novel mutation in MFSD8 gene. Eur J Med Genet 2014;57(11-12):607-12 doi: 10.1016/j.ejmg.2014.09.004.
7. Sharifi A, Kousi M, Sagné C, et al. Expression and lysosomal targeting of CLN7, a major facilitator superfamily transporter associated with variant late-infantile neuronal ceroid lipofuscinosis. Hum Mol Genet 2010;19(22):4497-514 doi: 10.1093/hmg/ddq381.
8. Tornoe J, Kusk P, Johansen TE, Jensen PR. Generation of a synthetic mammalian promoter library by modification of sequences spacing transcription factor binding sites. Gene 2002;297(1-2):21-32.
9. Morgan CJ, Pyne-Geithman GJ, Jauch EC, et al. Bilirubin as a cerebrospinal fluid marker of sentinel subarachnoid hemorrhage: a preliminary report in pigs. J Neurosurg 2004;101(6):1026-9 doi: 10.3171/jns.2004.101.6.1026.
10. Sullivan HG, Miller JD, Griffith RL, Carter W, Rucker S. Bolous versus steady-state infusion for determination of CSF outflow resistance. Ann Neurol 1979;5(3):228-38 doi: 10.1002/ana.410050304.
11. Pardridge WM. Drug transport in brain via the cerebrospinal fluid. Fluids Barriers CNS 2011;8(1):7 doi: 10.1186/2045-8118-8-7.
12. Dekaban AS. Changes in brain weights during the span of human life: relation of brain weights to body heights and body weights. Ann Neurol 1978;4(4):345-56 doi: 10.1002/ana.410040410.
13. Zacharia A, Zimine S, Lovblad KO, et al. Early assessment of brain maturation by MR imaging segmentation in neonates and premature infants. AJNR Am J Neuroradiol 2006;27(5):972-7
14. Caviness VS, Kennedy DN, Richelme C, Rademacher J, Filipek PA. The human brain age 7-11 years: a volumetric analysis based on magnetic resonance images. Cereb Cortex 1996;6(5):726-36 doi: 10.1093/cercor/6.5.726.
15. Epstein A, Williams K, Reddihough D, et al. Content validation of the Quality of Life Inventory-Disability. Child Care Health Dev 2019;45(5):654-59 doi: 10.1111/cch.12691.
16. Downs J, Jacoby P, Leonard H, et al. Psychometric properties of the Quality of Life Inventory-Disability (QI-Disability) measure. Qual Life Res 2019;28(3):783-94 doi: 10.1007/s11136-018-2057-3
17. Tangarorang J, Leonard H, Epstein A, Downs J. A framework for understanding quality of life domains in individuals with the CDKL5 deficiency disorder. Am J Med Genet A 2019;179(2):249-56 doi: 10.1002/ajmg.a.61012
18. Raat H, Landgraf JM, Oostenbrink R, Moll HA, Essink-Bot ML. Reliability and validity of the Infant and Toddler Quality of Life Questionnaire (ITQOL) in a general population and respiratory disease sample. Qual Life Res 2007;16(3):445-60 doi: 10.1007/s11136-006-9134-8

19. Laboratories ACoPSfCPF. ATS statement: guidelines for the six-minute walk test. Am J Respir Crit Care Med 2002;166(1):111-7 doi: 10.1164/ajrccm.166.1.at1102
20. Reuben DB, Magasi S, McCreath HE, et al. Motor assessment using the NIH Toolbox. Neurology 2013;80(11 Suppl 3):S65-75 doi: 10.1212/WNL.0b013e3182872e01
21. Gershon RC, Wagster MV, Hendrie HC, Fox NA, Cook KF, Nowinski CJ. NIH toolbox for assessment of neurological and behavioral function. Neurology 2013;80(11 Suppl 3):S2-6 doi: 10.1212/WNL.0b013e3182872e5f
22. McDonald CM, Henricson EK, Abresch RT, et al. The 6-minute walk test and other clinical endpoints in duchenne muscular dystrophy: reliability, concurrent validity, and minimal clinically important differences from a multicenter study. Muscle Nerve 2013;48(3):357-68 doi: 10.1002/mus.23905
23. Kuper WFE, van Alfen C, van Eck L, et al. Motor function impairment is an early sign of CLN3 disease. Neurology 2019;93(3):e293-e97 doi: 10.1212/WNL.0000000000007773
24. Bohannon RW, Wang YC, Bubela D, Gershon RC. Normative Two-Minute Walk Test Distances for Boys and Girls 3 to 17 Years of Age. Phys Occup Ther Pediatr 2018;38(1):39-45 doi: 10.1080/01942638.2016.1261981
25. Stahlhut M, Downs J, Leonard H, Bisgaard AM, Nordmark E. Building the repertoire of measures of walking in Rett syndrome. Disabil Rehabil 2017;39(19):1926-31 doi: 10.1080/09638288.2016.1212280
26. Franjoine MR, Gunther JS, Taylor MJ. Pediatric balance scale: a modified version of the berg balance scale for the school-age child with mild to moderate motor impairment. Pediatr Phys Ther 2003;15(2):114-28 doi: 10.1097/01.PEP.0000068117.48023.18
27. Franjoine MR, Darr N, Held SL, Kott K, Young BL. The performance of children developing typically on the pediatric balance scale. Pediatr Phys Ther 2010;22(4):350-9 doi: 10.1097/PEP.0b013e3181f9d5eb
28. Chen C-l. Validity, responsiveness, minimal detectable change, and minimal clinically important change of pediatric balance scale in children with cerebral palsy. Elsevier 2013;Volume 34, Issue 3 , March 2013,: Pages 916-22
29. Zyłka J, Lach U, Rutkowska I. Functional balance assessment with pediatric balance scale in girls with visual impairment. Pediatr Phys Ther 2013;25(4):460-6 doi: 10.1097/PEP.0b013e31829ddbc8
30. de Sousa Santos AC. The performance of children and adolescents with low vision on the Pediatric Balance Scale. Motricidade 2018;14(ISSN 1646-107X):71-78
31. Russell DJ, Rosenbaum PL, Cadman DT, Gowland C, Hardy S, Jarvis S. The gross motor function measure: a means to evaluate the effects of physical therapy. Dev Med Child Neurol 1989;31(3):341-52 doi: 10.1111/j.1469-8749.1989.tb04003.x
32. Ko J, Kim M. Reliability and responsiveness of the gross motor function measure-88 in children with cerebral palsy. Phys Ther 2013;93(3):393-400 doi: 10.2522/ptj.20110374
33. Nelson L, Owens H, Hynan LS, Iannaccone ST, Group A. The gross motor function measure is a valid and sensitive outcome measure for spinal muscular atrophy. Neuromuscul Disord 2006;16(6):374-80 doi: 10.1016/j.nmd.2006.03.005
34. Nathwani AC, Tuddenham EG, Rangarajan S, et al. Adenovirus-associated virus vector-mediated gene transfer in hemophilia B. N Engl J Med 2011;365(25):2357-65 doi: 10.1056/NEJMoa1108046
35. Nathwani AC, Reiss UM, Tuddenham EG, et al. Long-term safety and efficacy of factor IX gene therapy in hemophilia B. N Engl J Med 2014;371(21):1994-2004 doi: 10.1056/NEJMoa1407309
