# Supplementary material for: First-in-human high dose AAV9 intrathecal gene therapy for paediatric CLN7 disease: a phase 1, open-label, single ascending dose, non-randomised clinical trial
Source: eBioMedicine. 2025 Nov 27;123:106044. doi: 10.1016/j.ebiom.2025.106044 (PMC12703863; doi:10.1016/j.ebiom.2025.106044)
Supplement: Supplementary Material 3 [file mmc3.pdf]

# Data Safety Monitoring Board Charter

## *Phase I Intrathecal Lumbar Administration of AAV9/CLN7 for Treatment of CLN7 Disease*

## INTRODUCTION

The purpose of this charter is to define the responsibilities of the Data Safety Monitoring Board (DSMB), detail membership requirements, describe the data to be reviewed, delineate the meeting process, and outline the considerations and policies of the DSMB. The DSMB will act in an expert, independent advisory capacity to monitor participant safety and evaluate the efficacy and conduct of the study.

### Study Overview

- Study Title: Phase I Intrathecal Lumbar Administration of AAV9/CLN7 for Treatment of CLN7 Disease
- Study Sponsor/Investigator: Benjamin Greenberg, MD, MHS
- Financial Sponsor: UT Southwestern Medical Center
- Study design: Open-label, single-dose study of AAV9/CLN7 administered intrathecally (IT) through a lumbar puncture (LP) in patients with confirmed pathogenic or likely pathogenic mutations in the *MFSD8* gene.
- Phase: I
- Number of participants: Four (4)
- Number of sites: One (1)

## DSMB RESPONSIBILITIES

The DSMB's responsibilities are to:

- Review the research protocol, informed consent documents and plans for data and safety monitoring;
- Evaluate the progress of the trial, including periodic assessments of data quality and timeliness, participant recruitment, accrual and retention, participant risk versus benefit, trial site performance, and other factors that can affect study outcome;
- Consider factors external to the study when relevant information becomes available, such as scientific or therapeutic developments that may have an impact on participant safety or the ethics of the trial;
- Review study performance, make recommendations and assist in the resolution of problems Reported by the Sponsor/Investigator;
- Protect the safety of the study participants
- Review safety data to determine safety signals or trends;
- Ensure the confidentiality of the trial data and the results of monitoring; and,
- Assist by commenting on any problems with study conduct, enrollment, and sample size and/or data collection.

A DSMB member may resign at any point during the trial, when:

a) A member is not able to fulfill the responsibilities, as outlined in the Charter;

b) A member is found to have a Conflict of Interest (COI) or believes a COI exists.

The DSMB may disband after the study is complete. The study will be considered complete when the last participant dosed has been followed for two (2) years and the results of the study have been analyzed and reported to the DSMB. Long-term follow-up for safety is required by the FDA but will be considered a separate study protocol.

# DSMB MEMBERSHIP

The DSMB membership will consist of at least three (3) persons completely independent of the Sponsor/Investigator who have no financial, scientific, or other conflicts of interest with the trial. Current or past collaborators or associates of Dr. Benjamin Greenberg or any of the Co-Investigators must note any conflict of interest before their eligibility to serve on the DSMB is approved.

The DSMB members are selected by the Sponsor and must meet the following requirements:

- Be willing to serve as a DSMB member for the duration of the study;
- Comply with the conflict of interest policy specified in this charter;
- Not be on the list of Notice of Initiation of Disqualification Proceedings and Opportunity to Explain (NIDPOE) (https://www.accessdata.fda.gov/); and
- Not be on the debarred list of investigators (https://www.fda.gov/inspections-compliance-enforcement-and-criminal-investigations/).

The DSMB includes experts in or representatives from the fields of:

- Pediatric Neurology – Dr. Brenda Banwell, Dr. Kevin Flanigan
- Pediatric Cardiology – Dr. Barry Byrne
- Neuromuscular Disorders – Dr. Kevin Flanigan
- Immunology – Dr. Brenda Banwell, Dr. Barry Byrne
- Gene Therapy Clinical Trials – Dr. Barry Byrne, Dr. Kevin Flanigan
- Pediatric Clinical Trials – Dr. Brenda Banwell, Dr. Barry Byrne, Dr. Kevin Flanigan

Although DSMB members are expected to serve for the duration of the study, in the unlikely event that a member is unable to continue participation, the reason will be documented and a replacement member will be selected by the DSMB Chair, and/or Sponsor. The new member must have comparable expertise and qualifications to the DSMB member he/she is replacing.

## DSMB Chairperson

The DSMB Chairperson is nominated by the Sponsor and will be confirmed by DSMB vote at the first meeting. The DSMB members must provide a full consensus vote when electing the DSMB Chairperson. The Chairperson is responsible for overseeing the meetings and summarize all DSMB recommendations to the Sponsor with input from the DSMB in Executive Session. The Chairperson is the primary contact person for the DSMB.

### Conflict of Interest

DSMB members should have no relationship with the Sponsor/Investigator that could impair the member's ability to objectively review study data as set forth below:

- DSMB members must not have any real or perceived scientific, financial, professional, personal, proprietary, or other conflict of interest related to the conduct, outcome, or impact of the study. This may include having been or being employed by the Sponsor/Investigator, having a fiduciary interest in the Sponsor, conducting and/or managing the study, and/or having contact with participants during the course of regular clinical care;
- DSMB members must not be engaged in any simultaneously occurring competitive studies in any role that could pose a conflict of interest. DSMB members must also identify and disclose any concurrent service on other DSMBs of the same, related, or competing products;
- DSMB members must be independent from the Sponsor/Investigator, IRBs, regulatory agencies, or co-Investigators, steering committee members, advisory board members, Clinical Events Committee members, clinical care of the study participants, or any other capacity related to study operations. Collaborators or associates of the participating investigator(s) are not eligible to serve on the DSMB.

Individuals invited to serve on the DSMB as either voting or non-voting members must disclose any potential conflicts of interest, whether real or perceived. Conflicts of interest can include professional, proprietary, and miscellaneous interests as described in the NIH Grant Policy Statement and 45 CFR Part 94. Potential conflicts that develop during a member's tenure on a DSMB must also be disclosed. Written documentation attesting to an absence of conflict of interest is required annually.

### Confidentiality

All materials, discussions, and proceedings of the DSMB are privileged and confidential. DSMB members agree to use this information exclusively to accomplish the responsibilities of the DSMB. No communication of the deliberations or recommendations of the DSMB, either written or oral, may occur except as required for the DSMB to fulfill its responsibilities. Individual DSMB members are expected to maintain confidentiality regarding the study outside the DSMB (including, but not limited to the investigators, IRBs, regulatory agencies, or sponsor) except as authorized by the DSMB.

## BOARD PROCESSES

Prior to commencement of recruitment, the study team provides drafts or revisions of trial materials (the protocol, consent documents, report templates, and any other materials required) for the DSMB's review.

### Projected Schedule of Meetings

The first meeting is held by teleconference before initiation of the trial to discuss the materials and whether the trial is ready to commence, establish guidelines for monitoring and determine the format for future meetings. The Sponsor will prepare the agenda to 1) review the study materials, 2) appoint the DSMB Chairperson, 3) discuss the plan and timing for safety monitoring, 4) make recommendations to initiate the trial and/or modify the trial materials, and 5) review the charter.

Reports for both the open and closed sessions and plans for interim analyses (if applicable) should be established at the initial DSMB meeting, although changes throughout the trial may be requested by the DSMB. In addition, the DSMB members will vote to recommend commencement of the trial.

Routine meetings of the DSMB are generally held two times a year (or at other intervals determined by the DSMB), via conference call. Attendance at all meetings is highly critical for all DSMB members. Each DSMB member is specifically selected for his/her expertise and thus the member's consistent participation ensures rigorous monitoring throughout the course of the trial. Meetings are to be closed to the public because of participant confidentiality considerations. Reports will be submitted prior to a scheduled meeting for review by the DSMB.

All DSMB discussions are confidential. An *ad hoc* meeting of the DSMB may be called at any time by the Chairperson should ethical or patient safety issues arise. The suggestion to convene an ad-hoc meeting should be transmitted to the Sponsor and/or PI. Depending on the situation, this meeting may include the Chairperson alone, a quorum of the DSMB, or the full DSMB.

Safety data are reviewed regularly by the DSMB. The DSMB decides how frequently it needs to review safety data and the level of details to be provided.

## Meeting Format

DSMB meetings will generally be conducted via teleconference and facilitated by the Sponsor and/or PI, consisting of an open session, a restricted session, and a closed session. A quorum will consist of two (2) voting members of the DSMB. If only a quorum is present for voting, then the vote must be unanimous in order to pass. If the quorum vote is not unanimous, then the third DSMB member must vote to break the tie.

### *Open Session*

The open session may be attended by the Investigator/Sponsor and Co-Investigators. Sponsor representatives may attend the open session with DSMB members, during which time they can provide recruitment updates, information on study conduct, compliance, withdrawals, data quality, and non-confidential information regarding operational/logistical issues. This session gives the DSMB an opportunity to query the Sponsor about issues that have arisen during the review of safety data. Financial Sponsors may attend the meetings *ex officio*.

*Restricted Session*

The restricted session may be attended by the non-conflicted Investigator/Sponsor and Co-Investigators. Any Co-Investigator(s) (i.e. [Name specific conflicted team members]) that have a financial conflict of interest will recuse themselves from this session of the meeting. This session gives the DSMB an opportunity to query the Sponsor/Investigator and other non-conflicted co-Investigators about issues that have arisen during the review of safety data.

### *Closed Session*

The DSMB may move to a closed session that will be restricted to attendance by the voting DSMB members. Closed sessions may consist of a private review of the recommendations the DSMB wishes to make to the Sponsor and a formal vote on these recommendations.

If necessary, a second **open session** to clarify any questions that arise from the DSMB closed session may be held with the study staff. A second **closed session** may also be held, if needed. *Ad hoc* meetings may be held at any time should ethical or safety issues arise.

Each meeting, whether routine or *ad hoc*, must include a recommendation made by all three voting DSMB members to initiate, continue, place on hold, or terminate the trial. The vote may be postponed until further information is acquired. Should the DSMB decide to issue a termination recommendation, the full DSMB must vote.

## Meeting Minutes

Minutes of DSMB meetings will be kept of the open session.

### *Open Session*

Open session meeting minutes include at a minimum:

- Protocol number, study title, version
- DSMB meeting date
- Copy of the open session agenda
- A list of attendees, including DSMB members and any other persons present, listing their professional title and role at the meeting
- Information reviewed and related discussion during the open session, including rationale for recommendations provided by voting DSMB members
- A copy of the DSMB recommendation letter

The Sponsor is responsible for recording and generating meeting minutes of the open session. The DSMB Chair will share any guidance that results from the closed sessions to the Sponsor.

Draft minutes of open sessions will be sent to the DSMB Chair for review and approval within 5 business days of the meeting. The draft minutes will be reviewed by the DSMB Chair within 5 business days, and final minutes of the open session will be distributed to the DSMB members and the Sponsor within 5 business business days of the DSMB meeting. Final minutes will be distributed to DSMB members by secure email.

*Restricted Session*

Restricted session meeting minutes include at a minimum:

- Information reviewed and related discussion during the restricted session, including rationale for recommendations provided by voting DSMB members

The Sponsor is responsible for recording and generating meeting minutes of the open session. Minutes from the restricted session will be recorded outside of the open session minutes and will be kept confidentially by the Sponsor. This will be done to keep in-line with the Conflict Management plans of those study staff with financial conflict. The DSMB Chair will share any guidance that results from the closed sessions to the Sponsor.

### *Closed Session*

The DSMB Chair will draft the final recommendations of the closed sessions and will provide them to the Sponsor for review within 5 business days of the DSMB meeting. The recommendations will be reviewed by the Sponsor. A response to the DSMB will be shared by secure email within 5 business days.

Closed session meeting recommendations will not be divulged beyond the DSMB until after the study is closed unless either:

- The DSMB voting members approve the release for the purpose of preserving the integrity of the study and the safety of participants, or
- The FDA or other regulatory authority requires disclosure

## Sponsor Reports to the DSMB

The DSMB will have access to real-time review of participant data during the course of the study through access to the participant's electronic medical record. In addition, following the dosing of the first participant, a detailed review of safety will be conducted by the DSMB prior to dosing subsequent participants. After 60 days following dosing of each participant, the PI will submit a safety report to the DSMB for review. Requests for additional data by the DSMB can be made by communicating the request to the PI.

Other reports submitted will include:

- Immediate and interim subject data reviews. Data for individual subject reviews or for SAEs will be made available as soon as possible.
- Summaries of AEs and SAEs.
- Information necessary to review the conduct of the trial, including recruitment, enrollment, and unexpected problems.
- Information from nonclinical findings that may impact the safety assessment of the trial will be provided by the PI or his designee.
- The reports to the DSMB are considered privileged and not subject to disclosure except as required by law.
- Data summaries may be prepared upon written request by the DSMB to address a specific safety concern (email is an acceptable method of communication).

## DSMB Reports to the Sponsor

Following each meeting, the DSMB will issue a confidential report separate from the minutes of the open and closed sessions that will be sent to the Sponsor. The report includes a summary of the open session discussion and provides the DSMB's recommendations accompanied by clear, concise rationale for them. The report should contain sufficient information to explain the rationale for any specific actions by the DSMB without jeopardizing conduct or scientific integrity of the study. If no recommendations are made, the report may simply state, "The DSMB recommends that the study continue as planned."

The report should be presented to the Sponsor both in writing and orally. The DSMB Chair communicates directly with the Sponsor to allow them the opportunity to ask questions and discuss any recommendations. If the report does include DSMB recommendations for changes or termination of the study, the report must include a minimum amount of data such that the Sponsor can make a reasoned decision in response to the recommendation.

If the Sponsor accepts the recommendations of the DSMB, the Sponsor will be responsible for implementing the actions in response. In the event the study must be amended, the Sponsor will prepare and submit the amendment to the DSMB for approval prior to implementing amendment changes.

If the Sponsor rejects the DSMB's recommendations, the Sponsor must provide the DSMB with a written explanation of their decision and supporting rationale within 5 business days. If the DSMB has recommended that the study be stopped but the Sponsor decides to continue the study, the Sponsor will inform all concerned regulatory authorities of its decision to continue the study despite the DSMB's recommendation. Public disclosure of the decision to stop the study is at the discretion of the Sponsor. The DSMB will not make any public announcements.

# DSMB CONSIDERATIONS AND POLICIES

## Stopping/Discontinuation Rules

The DSMB will have the responsibility and authority to stop or suspend the trial based on its review of the data and the pre-determined stopping rules. If the DSMB determines that the trial should be stopped or suspended, they will notify the Sponsor/PI as soon as feasible. The PI will be responsible for notifying the IRB within three business days.

The DSMB will review the study data that the PI will submit listed below:

- A narrative summary of trial activity to date,
- A line listing of all AEs reportable per protocol,
- A narrative summary assessment of any safety concerns including:
    - AE and SAE trends,
    - Unanticipated problems relating directly to protocol-driven activities,
    - Participants withdrawn for safety reasons,
    - Trial halting or pausing activity, and
    - Other events relating to the overall safety of the trial.

After each DSMB review, a recommendation as to whether the study is to continue, be modified, or be terminated will be provided in a summary report. All SAEs, all unanticipated problems, and all IND Safety Reports will be reported by the PI to the DSMB at the same time they are submitted to the IRB. The DSMB will be notified immediately if pausing or halting rules are met and the DSMB will provide a recommendation for continuation, modification, or termination of the study. The PI will submit the written DSMB summary reports with recommendations to the IRB.

Halting the study requires immediate discontinuation of study agent administered for all participants and suspension of enrollment until a decision is made whether or not to continue study agent administration.

Halting Criteria for the Protocol:

- Two (2) or more participants experience the same or similar SAEs that are unexpected and are possibly, probably, or definitely related to the study agent
- Three (3) or more of the same or similar AE in different subjects that are grade 3 or above and are unexpected and possibly, probably, or definitely related to the study agent
- Any safety issue that the site investigators determine should halt the study

Reporting of Study Halting: If a halting requirement is met, a description of the event(s) or safety issue(s) must be reported by the PI within one business day by email AND the PI must inform the IRB that a halting rule has been met.

Resumption of a Halted Study: The Sponsor/Investigator in collaboration with the the DSMB will determine if it is safe to resume the study. The conditions for resumption of the study will be defined in an official notification. The PI will notify the IRB of the decision to resume the study.

## Discrepancies with Assessments Concerning Unanticipated Problems

On occasion, there may be disagreements between the Sponsor/Investigator and the DSMB regarding the assessment and/or management of an event that qualifies as an unanticipated problem. The following excerpt gives guidance for cases where there is a difference of opinion among the DSMB (referred to as the “monitoring entity” in the excerpt below) and the Sponsor/Investigator http://www.hhs.gov/ohrp/policy/advevntguid.html.

*If the Sponsor/Investigator determines that an adverse event is not an unanticipated problem, but the monitoring entity subsequently determines that the adverse event does in fact represent an unanticipated*

*problem (for example, due to an unexpectedly higher frequency of the event), the monitoring entity should report this determination to the Sponsor/Investigator, and such reports must be promptly submitted by the Sponsor/Investigator to the IRB (45 CFR 46.103(b)(5)).*

Please note, the DSMB and the Sponsor/Investigator may have iterative discussions regarding the assessment and may later come to agreement regarding the assessment and/or management of an AE. In cases where the DSMB and Sponsor/Investigator come to an agreement after discussions and the event is determined not to be an unanticipated problem, the Sponsor/Investigator is not required to report the event as an unanticipated problem to the IRB. Such discussions should take place promptly so as not to delay appropriate reporting to the IRB.

Please also note that additional reporting requirements [e.g., to the Food and Drug Administration (FDA) and the IRB] are not part of the DSMB process and are the Sponsor/Investigator’s responsibility

# REVIEW OF DATA

## Safety Data

The primary charge of the DSMB is to monitor the study for participant safety. The safety and related data the DSMB will review includes:

Reports will include the following:

- a brief narrative of the study status, including the target enrollment, current and projected time to completing enrollment, and a brief description of any significant events and/or difficulties;
- a brief narrative for each participant describing gender, age, race and ethnicity and other relevant demographic characteristics, as well as a brief description of his/her study status (i.e., dose level, visit number, adverse event information);
- a timeline outlining the study progress relative to visit number for each participant, as well as time points for each SAE/Dose limiting toxicity, and a total for Adverse Events (AEs) for each participant;
- a summary of AEs by classification;
- a listing of AE details grouped by participant;
- a listing of SAE details grouped by participant;
- a listing of deaths;
- a summary of clinically significant laboratory test results;
- a listing of protocol deviations.

## Expedited Reporting of Unanticipated Problems and Serious Adverse Events (SAEs)

### *Unanticipated Problems*

Unanticipated problems are 1) unexpected events that are 2) related or possibly related to participation in the research that 3) place subjects or others at greater risk of harm than was previously known or recognized. All three criteria above must be met to qualify the event as an unanticipated problem. The Office for Human Research Protections (OHRP), the Department of Health and Human Services (HHS), provides a complete definition and the following guidance for reporting unanticipated problems to the Institutional Review Board(s) (IRBs): *Guidance on Reviewing and Reporting Unanticipated Problems*

*Involving Risks to Subjects or Others and Adverse Events* (http://www.hhs.gov/ohrp/policy/advevntguid.html). The DSMB Chair reviews the unanticipated problems to determine if further action is required.

### *Serious Adverse Events*

All Serious Adverse Events (SAEs) that are life threatening (regardless of expectedness, relatedness, or if they meet the definition for unanticipated problems) must be reported to the DSMB Chair within 48 hours of the PI receiving notification of the event. The report will include a description of the event, as well as the Sponsor/Investigator's assessment of expectedness, relatedness and other information, as relevant. Any action taken by the investigative team should be provided in the report. The DSMB Chair will be provided with this information but will provide an independent assessment on attribution and expectedness, as well as whether further action is recommended (e.g., collection of follow up information).

### *Serious Adverse Event Reporting: Content and Format*

Serious adverse events (SAEs) will be monitored by the DSMB Chair in real time throughout the study. SAEs must be reported by the Sponsor to the DSMB Chair via email within one business day of learning of the event.

The serious adverse event report will include, but need not be limited to:

(1) The date of the event

(2) Designation of the report as an initial report or a follow-up report, identification of all safety reports previously filed for the clinical protocol concerning a similar adverse event, and an analysis of the significance of the adverse event in light of previous similar reports

(3) Clinical site

(4) The Sponsor/Investigator

(5) FDA's Investigational New Drug (IND) application number

(6) Vector type, e.g., adeno-associated virus

(7) Vector subtype, if relevant

(8) gene delivery method, e.g., in vivo transduction

(9) Route of administration, e.g., intrathecal

(10) Dosing schedule

(11) A complete description of the event

(12) Relevant clinical observations

(13) Relevant clinical history

(14) Relevant tests that were or are planned to be conducted

(15) Date of any treatment of the event

(16) The suspected cause of the event. Reporting will be based upon a standard template (https://osp.od.nih.gov/wp-content/uploads/Edited-Adverse-Event-Template-revised-4-12-17.docx). A copy of this report will also be sent to the IRB, IBCSC, and FDA according to regulatory requirements described in the protocol section on Safety Reporting.

All participant withdrawals will be monitored by the DSMB Chair in real time throughout the study. All participant withdrawals must be reported by the Sponsor/Investigator to the DSMB Chair via email in a PDF file, within one business day of learning of the withdrawal.

### Other Data

#### *Effectiveness*

The DSMB monitors effectiveness outcomes to determine relative risk/benefit, futility, or for early termination due to overwhelming effectiveness. The DSMB is charged with recommending early termination based on positive efficacy results only when the data are truly compelling, and the risk of a false positive conclusion is acceptably low. If interim data suggest that the product under study is of no benefit (no trend indicating superiority of the product), or that accrual rates are too low and/or that noncompliance is too great to provide adequate power for identifying the specified benefit, the DSMB may consider whether continuation of the study is futile and may recommend termination on this basis.

#### *Study Conduct*

The DSMB reviews data related to study conduct. Data to be reviewed and listed in the DSMB reports regarding study conduct includes: summary of protocol violations, completeness and timeliness of study visit data, enrollment eligibility and ineligibility information, noncompliance, unanticipated problems, information concerning withdrawal of participants. The DSMB may issue recommendations regarding study conduct when concerns arise that aspects of study conduct may threaten participant safety or study integrity. For example, if data presented to DSMB are not current, the DSMB will not be able to meet its responsibility to ensure the study continues to be safe for its current and future participants.

#### *Consideration of external data*

The DSMB may consider data from other studies or external sources during its deliberations, if available, as these results may have a profound impact on the status of the participants and design of the current study.

## OTHER

### Amendments to the DSMB Charter

This DSMB charter can be amended as needed during the course of the study. All amendments will be documented with sequential version numbers and revision dates and will be recorded in the open session DSMB meeting minutes. Each revision will be reviewed and agreed upon by the Investigator/Sponsor and DSMB.

### Archiving

All DSMB documentation and records will be retained by UT Southwestern Medical Center for a time period of 5 years after completion of the study. Access to archived data will be controlled by UT Southwestern Medical Center which will release the information only as specified in this charter or as required by law.

## DSMB CHARTER SIGNATURE PAGE

____________________________________ __________

Kevin Flanigan, MD | Date
DSMB Chair

______________________________________ ___________
Brenda Banwell, MD Date
DSMB Member

Barry Byrne, MD, PhD
DSMB Member

Date

Benjamin Greenberg, MD, MHS | Date
Sponsor-Investigator
