# Supplementary material for: First-in-human high dose AAV9 intrathecal gene therapy for paediatric CLN7 disease: a phase 1, open-label, single ascending dose, non-randomised clinical trial
Source: eBioMedicine. 2025 Nov 27;123:106044. doi: 10.1016/j.ebiom.2025.106044 (PMC12703863; doi:10.1016/j.ebiom.2025.106044)
Supplement: Supplementary Material 4 [file mmc4.doc]

**DSMB Report**

***-Open Session-***

# Title Page

**Phase I Intrathecal Lumbar Administration of AAV9/CLN7 for Treatment of CLN7 Disease**

**PI- Benjamin Greenberg, MD**

# Table of Contents

[**Title Page i**](#__RefHeading___Toc194114881)

[**Table of Contents ii**](#__RefHeading___Toc194114882)

[**Report Summary 1**](#__RefHeading___Toc194114883)

[Protocol Synopsis 2](#__RefHeading___Toc194114884)

[Brief Statement of Purpose of Trial 2](#__RefHeading___Toc194114886)

[Projected Timetable and Schedule 2](#__RefHeading___Toc194114887)

[Recruitment and Participant Status: Figures and Tables 4](#__RefHeading___Toc194114895)

[Figure 1: Overall Study Status 5](#__RefHeading___Toc194114897)

[Figure 2: Enrollment: Actual vs. Expected 6](#__RefHeading___Toc194114898)

[**Table 1: Participant Enrollment Status** 7](#__RefHeading___Toc194114899)

[Table 2: Reasons for Screen Failures 8](#__RefHeading___Toc194114900)

[Table 3: Protocol Deviations 9](#__RefHeading___Toc194114902)

[Table 4: Demographic and Key Baseline Characteristics 10](#__RefHeading___Toc194114903)

[Safety Assessments and values: Tables and Listings 12](#__RefHeading___Toc194114905)

[Table 6: Incidence of Adverse Events by Body System and Preferred Term 13](#__RefHeading___Toc194114907)

[Table 7: Severity of Adverse Events by Preferred Term 14](#__RefHeading___Toc194114908)

[Listing 1: Serious Adverse Events 15](#__RefHeading___Toc194114909)

[Listing 2: Deaths 16](#__RefHeading___Toc194114910)

[Listing 3: Adverse Events 17](#__RefHeading___Toc194114911)

[Table 8: Laboratory Test Results Summary 18](#__RefHeading___Toc194114912)

[Listing 4: Clinically Significant Abnormal Lab Values 19](#__RefHeading___Toc194114913)

Table 9: Sirolimus/Tacrolimus trough levels

Table 10: Elispot results

Table 11: Cardiac results

Table 12: NCS

# Report Summary

Protocol Synopsis

### Brief Statement of Purpose of Trial

### Projected Timetable and Schedule

# Study Administration

**Recruitment and Participant Status:**

Figures and Tables

### Figure 1: Overall Study Status


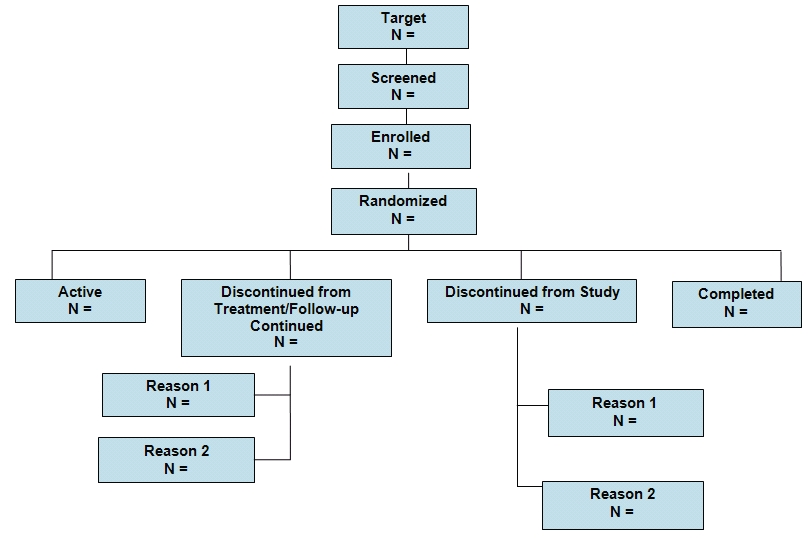


Data as of: July 16^th^ 2021

Recruitment Start Date:

### Figure 2: Enrollment: Actual vs. Expected

Data as of:_________

Date of report:_________

0

10

20

30

40

50

60

70

Mar-06

Apr-06

May-06

Jun-06

Jul-06

Aug-06

Sep-06

Oct-06

Nov-06

Dec-06

Jan-07

Feb-07

Mar-07

Apr-07

May-07

Jun-07

**Months**

**Participants**

Target Enrollment

Monthly Enrollment

## Table 1: Participant Enrollment Status

Data as of:_________

Date of report:_________

|  | **N** | **%** |
| --- | --- | --- |
| Enrolled |  | 100 |
| Active |  |  |
| Completed |  |  |
|  |  |  |
| **Discontinued Treatment/Follow-up Continued** |  | 100 |
| Personal Reason |  |  |
| Serious Adverse Event/ AE |  |  |
|  |  |  |
| **Discontinued from Study** |  | 100 |
| Lost to follow- up |  |  |
| SAE/AE |  |  |
| Withdrew Consent |  |  |

- *These are examples. Use categories relevant to protocol.*

### Table 2: Reasons for Screen Failures

Data as of:_________

Date of report:_________

| **Reason** | **Total N** | **Total %*** |
| --- | --- | --- |
|  |  |  |
|  |  |  |
|  |  |  |
| **Total Screened** |  |  |
| **Total Screen Failures** |  |  |

# * - % of the total number screened

### Table 3: Protocol Deviations

Data as of:_________

Date of report:_________

|  | **Protocol Deviation*** | **Total** | ***Since Last DSMB Report*** |
| --- | --- | --- | --- |
| 1 |  |  |  |
| 2 |  |  |  |
| 3 |  |  |  |
| 4 |  |  |  |
| 5 |  |  |  |
| 6 |  |  |  |
|  | **Total # of Deviations** |  |  |
|  | **Participants Enrolled** |  |  |
|  | **Deviations per Participant** |  |  |

**Possible deviations may include:*

- *Did not meet inclusion/exclusion criteria*
- *Visit noncompliance/incomplete visit*
- *Participant taking concomitant drugs which are not allowed*
- *Assessments outside protocol window*
- *Failure to obtain informed consent*

### Table 4: Demographic and Key Baseline Characteristics

Data as of:_________

Date of report:_________

| **Characteristics** | | **N (%)** |
| --- | --- | --- |
|
|  |
| **Total Enrolled:** | |  |
| **Gender** | Male |  |
| Female |  |
| **Ethnicity** | Hispanic or Latino |  |
| Not Hispanic or Latino |  |
| Unknown or not reported |  |
| **Race** | American Indian/Alaska Native |  |
| Asian |  |
| Black or African American |  |
| Native Hawaiian or Other Pacific Islander |  |
| White |  |
| More than one race |  |
| Unknown or not reported |  |
| **Clinical Features/**  **Stratification** | BMI ≥ 30* |  |
|  |  |
|  |  |  |
| **Age** | Mean |  |
| Median |  |
| Standard Deviation |  |
| Minimum |  |
| Maximum |  |

** This is an example, needs to be protocol specific.*

#

Safety Assessments for All Participants:

Tables and Listing

### Table 6: Incidence of Adverse Events by Body System and Preferred Term

Data as of:_________

Date of report:_________

| **Body System and Preferred Term** | **Total N=n*** | **Total N= (%)**** | **Total N=Events***** |
| --- | --- | --- | --- |
| **Overall** |  |  |  |
| **Cardiovascular** |  |  |  |
| Myocardial Infarction |  |  |  |
| Increased Blood Pressure |  |  |  |
| etc. |  |  |  |
|  |  |  |  |
| **Genitourinary** |  |  |  |
| Yeast Infection |  |  |  |
| Vaginal Bleeding |  |  |  |
| etc. |  |  |  |
|  |  |  |  |
| **Gastrointestinal** |  |  |  |
|  |  |  |  |
| **etc…** |  |  |  |

** Number of participants experiencing an adverse event (participant is to be counted only once for each adverse event)*

*** % of total number of participants in the study*

**** Number of events*

*This table can present overall incidence of adverse events as shown above; or adverse events related to the intervention as judged by the investigator; or treatment emergent events.*

### Table 7: Severity of Adverse Events by Preferred Term

Data as of:_________

Date of report:_________

| **Preferred Term*** | **Total N=Mild n** (%)***** | **Total N=Moderate n (%)** | **Total N=Severe n (%)** |
| --- | --- | --- | --- |
| Headache |  |  |  |
| Pain |  |  |  |
| etc. |  |  |  |

** For each preferred term, sort by most common event in descending order of incidence*

*** Number of participants experiencing a certain severity of an adverse event where each participant is counted only once at the highest level of severity for the event*

**** % of participants experiencing a certain severity of an adverse event*

*This table can present severity of all adverse events sorted by preferred term in descending order of incidence as shown above; or adverse events related to the intervention as judged by the investigator; or treatment emergent events.*

### Listing 1: Serious Adverse Events

Data as of:_________

Date of report:_________

| **Participant ID** | **Onset Date** | **Stop Date** | **Expected**  **(Y/N)** | **Relationship to Intervention***  **(Y/N)** | **Outcome**** | **Description of SAE** |
| --- | --- | --- | --- | --- | --- | --- |
|  |  |  |  |  |  |  |
|  |  |  |  |  |  |  |
|  |  |  |  |  |  |  |

** Definite, Possible, Not Related*

*** Outcome:*

*Recovered, without treatment*

*Recovered, with treatment*

*Still Present, no treatment*

*Still Present, being treated*

*Residual effect(s) present – no treatment*

*Residual effect(s) present- being treated*

*Subject died*

| **Participant ID** | **Date of Death** | **Cause of Death** | **Relationship to Intervention*** |
| --- | --- | --- | --- |
|  |  |  |  |
|  |  |  |  |
|  |  |  |  |
|  |  |  |  |
|  |  |  |  |
|  |  |  |  |
|  |  |  |  |
|  |  |  |  |
|  |  |  |  |
|  |  |  |  |

### Listing 2: Deaths

Data as of:_________

Date of report:_________

** Definite, Possible, Not Related*

### Listing 3: Adverse Events *

Data as of:_________

Date of report:_________

| **Participant ID** | **Days on Intervention** | **Preferred Term** | **Relationship to Intervention**** | **Severity** | **Serious (Y/N)** | **Outcome***** |
| --- | --- | --- | --- | --- | --- | --- |
|  |  |  |  |  |  |  |
|  |  |  |  |  |  |  |
|  |  |  |  |  |  |  |

** This listing could be provided in two ways – sorted by Preferred Term or sorted by Participant ID.*

*** Definite, Possible, Not Related*

**** Outcome:*

*Recovered, without treatment*

*Recovered, with treatment*

*Still Present, no treatment*

*Still Present, being treated*

*Residual effect(s) present – no treatment*

*Residual effect(s) present- being treated*

*Subject died*

### Table 8: Laboratory Test Results Summary*

Data as of:_________

Date of report:_________

| **Laboratory Test** | **Sample Study Visits** | **N** | **Mean** | **SD** | **Min** | **Median** | **Max** | **N** | **Mean** | **SD** | **Min** | **Median** | **Max** |
| --- | --- | --- | --- | --- | --- | --- | --- | --- | --- | --- | --- | --- | --- |
|  |  |  |  |  |  |  |  |  |  |  |  |  |  |
| **Test 1** | Screening |  |  |  |  |  |  |  |  |  |  |  |  |
|  | 6 Months |  |  |  |  |  |  |  |  |  |  |  |  |
|  | 12 Months |  |  |  |  |  |  |  |  |  |  |  |  |
|  | 24 Months |  |  |  |  |  |  |  |  |  |  |  |  |
|  | 36 Months |  |  |  |  |  |  |  |  |  |  |  |  |
|  |  |  |  |  |  |  |  |  |  |  |  |  |  |
|  |  |  |  |  |  |  |  |  |  |  |  |  |  |
| **Test 2** | Screening |  |  |  |  |  |  |  |  |  |  |  |  |
|  | 6 Months |  |  |  |  |  |  |  |  |  |  |  |  |
|  | 12 Months |  |  |  |  |  |  |  |  |  |  |  |  |
|  | 24 Months |  |  |  |  |  |  |  |  |  |  |  |  |
|  | 36 Months |  |  |  |  |  |  |  |  |  |  |  |  |
|  |  |  |  |  |  |  |  |  |  |  |  |  |  |
|  |  |  |  |  |  |  |  |  |  |  |  |  |  |
| **Etc…** | Screening |  |  |  |  |  |  |  |  |  |  |  |  |
|  | 6 Months |  |  |  |  |  |  |  |  |  |  |  |  |
|  | 12 Months |  |  |  |  |  |  |  |  |  |  |  |  |
|  | 24 Months |  |  |  |  |  |  |  |  |  |  |  |  |
|  | 36 Months |  |  |  |  |  |  |  |  |  |  |  |  |

| **Laboratory Test** |  | Pre-Screening | Screening/ Baseline | Gene Transfer  (inpatient) | | | | 24-Month Follow-up | | | | | | |
| --- | --- | --- | --- | --- | --- | --- | --- | --- | --- | --- | --- | --- | --- | --- |
|  | **Visit** | 0 | 1 | 2 | | | | 3 | 4* | 5* | 6 | | 7* | 8 |
|  | Days in study | Before -30 | -28 to -7 | -1 | 0 | 1 | 2 | 7 | 14 | 21 (±2) | | 30  (±2) | 44  (±2) | 60  (±2) |
|  |  |  |  |  |  |  |  |  |  |  | |  |  |  |
|  |  |  |  |  |  |  |  |  |  |  | |  |  |  |
|  |  |  |  |  |  |  |  |  |  |  | |  |  |  |
|  |  |  |  |  |  |  |  |  |  |  | |  |  |  |
|  |  |  |  |  |  |  |  |  |  |  | |  |  |  |
|  |  |  |  |  |  |  |  |  |  |  | |  |  |  |
|  |  |  |  |  |  |  |  |  |  |  | |  |  |  |
|  |  |  |  |  |  |  |  |  |  |  | |  |  |  |
|  |  |  |  |  |  |  |  |  |  |  | |  |  |  |
|  |  |  |  |  |  |  |  |  |  |  | |  |  |  |
|  |  |  |  |  |  |  |  |  |  |  | |  |  |  |
|  |  |  |  |  |  |  |  |  |  |  | |  |  |  |
|  |  |  |  |  |  |  |  |  |  |  | |  |  |  |
|  |  |  |  |  |  |  |  |  |  |  | |  |  |  |
|  |  |  |  |  |  |  |  |  |  |  | |  |  |  |
|  |  |  |  |  |  |  |  |  |  |  | |  |  |  |
|  |  |  |  |  |  |  |  |  |  |  | |  |  |  |
|  |  |  |  |  |  |  |  |  |  |  | |  |  |  |

** Table may include lab test results that are clinically significant, as defined by the protocol, or ALL lab test results*. *Final format is determined by the DSMB.*

### Listing 4: Clinically Significant Abnormal Lab Values

Data as of:_________

Date of report:_________

| **Participant ID** | **Visit** | **Age** | **Gender** | **Lab Panel** | **Lab Test** | **Result** |
| --- | --- | --- | --- | --- | --- | --- |
|  |  |  |  |  |  |  |
|  |  |  |  |  |  |  |
|  |  |  |  |  |  |  |

*Table 9: Sirolimus/Tacrolimus trough levels*

*Table 10: Elispot results*

*Table 11: Cardiac results*

*Table 12: NCS*

*Efficacy data*

*Futility data*
