# Supplementary material for: First-in-human high dose AAV9 intrathecal gene therapy for paediatric CLN7 disease: a phase 1, open-label, single ascending dose, non-randomised clinical trial
Source: eBioMedicine. 2025 Nov 27;123:106044. doi: 10.1016/j.ebiom.2025.106044 (PMC12703863; doi:10.1016/j.ebiom.2025.106044)
Supplement: Supplementary Material 5 [file mmc5.pdf]

Vigene Biosciences
Excellence in Gene Delivery

CERTIFICATE OF ANALYSIS

**Sample Name**	scAAV9/CLN7 Drug Substance and Drug Product	**Sample Type**	Drug Substance/Product
**Lot Number**	0072-20001	**Manufacturer**	Vigene Biosciences

**DRUG SUBSTANCE POOL, PRE-FILTRATION, POST-DILUTION**				
**Assay**	**Testing Lab / SOP Code**	**Specifications**	**Results**	**Disposition**
**Empty/Full Capsid Ratio:**<br>• **qPCR**<br>• **ELISA Capsid Titer**	Vigene / S4002<br>Vigene / S1053	> 1E14 vg/mL<br>> 34% Full	1.41E14 vg/mL<br>46% Full	Pass
**Bioburden (Spread Plate Count)**	Clongen / CB121	< 10 CFU/mL	<10 CFU/mL	Pass
**DRUG PRODUCT**				
**Assay**	**Testing Lab / SOP Code**	**Specifications**	**Results**	**Disposition**
**Residual Polyethylenimine (UHPLC-CAD)**	Bioreliance / 452019QUAL.BUK and 452019GMP.BUK	Report Results	Not Detected[1]	N/A
**Residual Plasmid DNA (qPCR; ITR, Ad helper, AAV helper plasmids)**	Wuxi AppTec / 33492.1	Report Results	Total encapsidated DNA: >1E8 copies/5 µL<br>Total residual DNA: >1E8 copies/5 µL	N/A
**Genomic ID by Sanger**	Microsynth / 19999, 20002, 20024, 09812, and 0979	Match	Match	Pass
**Biologics – Sterility Test- Immersion (21 CFR 610.12)**	Clongen / CB112a	Sterile	No growth<br>(3 vials tested)	Pass
**Bacterial Endotoxin (LAL/Chromogenic Method)**	Vigene / S3134	NMT ≤ 0.20 EU/ 1E14 vg	0.09 EU/1E14 vg	Pass
**DLS (Aggregation, > 20 nm)**	Wuxi AppTec / 21200.5 and 21200.F	<50% aggregated	5.90%	Pass
**In Vitro Potency**	UTSW	Report Results	0.0020 expression of CLN7/GAPDH per 1E5 vg/cell	N/A

Table Continued on Page 2

Vigene Biosciences
Excellence in Gene Delivery

CERTIFICATE OF ANALYSIS

Table Continued from Page 1

Assay	Testing Lab / SOP Code	Specifications	Results	Disposition
**Empty/Full Capsid Ratio:**<br>• **qPCR**<br>• **ELISA Capsid Titer**	Vigene / S4002<br>Vigene / S1053	>34% Full	42% Full	Pass
**Appearance (Visual Inspection)**	Vigene / S1032	Clear, colorless solution; no visible particles	Clear, colorless solution; no visible particles (100% of vials)	Pass
**Replication Competent AAV (Cell Culture/qPCR)**	Wuxi AppTec / C19754.2	Not Detected	Not Detected	Pass
**AAV9 Vector Identity (Western Blot)**	Vigene / S1034	Confirmed	Specific AAV VP1/VP2/VP3 confirmed	Pass
**Viral Genome Determination (qPCR)**	Vigene / S4002	1.2 – 2.0E14 vg/mL	1.41E14 vg/mL	Pass
**Residual Benzonase (ELISA)**	Vigene / S1041	< 1 pg/1E9 vg	< 0.001 pg/1E9 vg	Pass
**pH USP <791>**	Vigene / S1035	7.1 – 7.7	7.32	Pass
**Osmolality USP <785>**	Vigene / S1058	500 – 800 mOsm/kg of H_2O	695 mOsm/kg of H_2O	Pass
**Vector (rAAV9) Purity (SDS-PAGE)**	Vigene / S1033	VP1, VP2, and VP3 and no other apparent bands	VP1, VP2, and VP3 bands identified and no other apparent bands	Pass
**Residual Host Cell Protein (ELISA)**	Vigene / S1028	< 350 ng/mL	< 2 ng/mL	Pass
**Residual Host Cell DNA (qPCR)**	Vigene / S1027	< 1 µg/mL	0.34 µg/mL	Pass

Table Continued on Page 3

Vigene Biosciences
Excellence in Gene Delivery

CERTIFICATE OF ANALYSIS

Table Continued from Page 2

Assay	Testing Lab / SOP Code	Specifications	Results	Disposition
**Container-Closure Integrity Test, Helium Leak Detection Sample Analysis USP <1207>**	Amri / USP <1207>, ASTM F2391-05 (2016), and TP-22119-00-071320-C101, Rev. 0	Pass	No leak detected	Pass

1. *The detection limit and lower quantitation limit for the residual polyethylenimine test was 50 µg/mL and 100 µg/mL, respectively.*

Quality Control Lead Signature ____________________ **Date** 28 Dec 20

Quality Assurance Lead Signature ____________________ **Date** 28Dec20

Revision History

Rev. 01 – Updated residual PEI test result and added assay detection limit and lower quantitation limit information.

Rev. 00 – New document.
